# Enantioselective Synthesis of β-l-5-[(*E*)-2-Bromovinyl)-1-((2*S*,4*S*)-2-(hydroxymethyl)-1,3-(dioxolane-4-yl) Uracil)] (l-BHDU) *via* Chiral Pure l-Dioxolane

**DOI:** 10.1021/acs.joc.4c00399

**Published:** 2024-06-20

**Authors:** Yugandhar Kothapalli, Chung K. Chu, Uma S. Singh

**Affiliations:** Department of Pharmaceutical and Biomedical Sciences, College of Pharmacy, University of Georgia, Athens, Georgia 30602, United States

## Abstract

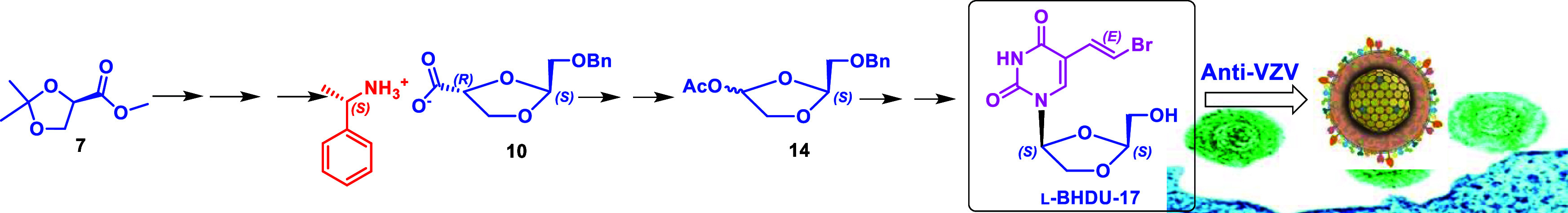

β-l-5-((*E*)-2-Bromovinyl)-1-((2*S*,4*S*)-2-(hydroxymethyl)-1,3-(dioxolane-4-yl) uracil (l-BHDU, **17**) is a potent and selective inhibitor of the varicella-zoster virus (VZV). l-BHDU (**17**) has demonstrated excellent *anti*-VZV activity and is a preclinical candidate to treat chickenpox, shingles (herpes zoster), and herpes simplex virus 1 (HSV-1) infections. Its monophosphate prodrug (POM-l-BHDU-MP, **24**) demonstrated an enhanced pharmacokinetic and antiviral profile. POM-l-BHDU-MP (**24**), *in vivo*, effectively reduced the VZV viral load and was effective for the topical treatment of VZV and HSV-1 infections. Therefore, a viable synthetic procedure for developing POM-l-BHDU-MP (**24**) is needed. In this article, an efficient approach for the synthesis of l-BHDU (**17**) from a readily available starting material is described in 7 steps. An efficient and practical methodology for both chiral pure l- & d-dioxolane **11** and **13** were developed *via* diastereomeric chiral amine salt formation. Neutralization of the amine carboxylate salt of l-dioxolane **10** provides enantiomerically pure l-dioxane **11** (ee ≥ 99%). Optically pure **11** was utilized to construct the final nucleoside l-BHDU (**17**) and its monophosphate ester prodrug (POM-l-BHDU-MP, **24**). Notably, the reported process eliminates expensive chiral chromatography for the synthesis of chiral pure l- & d-dioxolane, which offers avenues for the development and structure–activity relationship studies of l- & d-dioxolane-derived nucleosides.

## Introduction

Varicella-zoster virus (VZV) is a highly contagious alpha herpesvirus that causes chickenpox (varicella) and shingles (herpes zoster).^1^ The Centers for Disease Control and Prevention (CDC) has estimated that in the U.S., there are approximately one million cases of zoster annually.^2^ People over the age of 50, immunocompromised persons, HIV-infected people, and organ transplant patients are at a higher risk of VZV infections.^3,4^ The VZV infection induces major complications; it causes a painful skin vesicular rash. A significant problem of shingles is postherpetic neuralgia, which is long lasting pain that persist for months and years.^5^ Therefore, there is a need for new antivirals that are more effective and safer for the treatment and management of VZV infection than the currently available regiments. However, vaccination is available for both stages of VZV infections, but these can only be utilized in healthy persons.^6^

**Figure 1:**
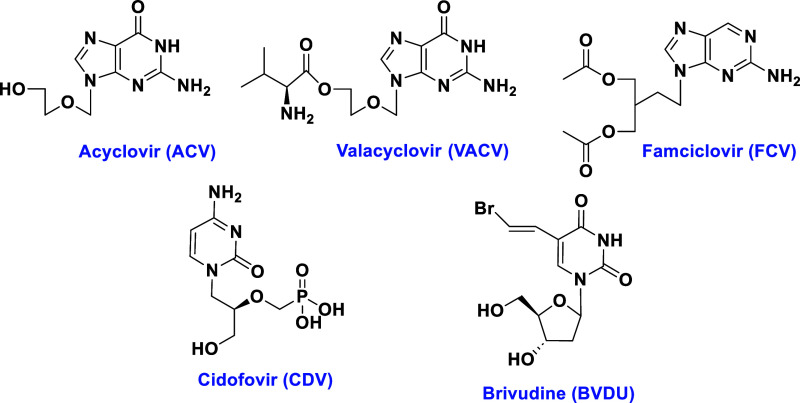
Structures of antiviral drugs for the treatment of VZV.

Acyclovir (ACV), valacyclovir (VACV), and famciclovir (FCV, Figure 1) are approved drugs for the treatment of VZV infection.^7^ However, these drugs are not highly effective against VZV, and large doses are required, which usually promotes drug resistance.^8^ Cidofovir (CDV), a broad-spectrum antiviral, is active against VZV, but it is associated with nephrotoxicity and lack of oral administration.^7^ Another antiviral drug, brivudine (BVDU), is approved to treat VZV infection in Europe.^9,10^ A major drawback associated with BVDU is that it metabolizes in the liver into bromovinyl uracil (BVU).^9^ BVU impedes the activity of dihydropyridine dehydrogenase (DPD), which catabolizes thymidine and uracil. Thus, cancer patients undergoing cotreatment with 5-fluorouracil (5-FU) and BVDU will have life threatening side effects.^11,12^ Due to the significant problems of currently approved drugs, there is an urgent need for new safe and effective antivirals to treat VZV infection and viral resistant strains.

**Figure 2:**
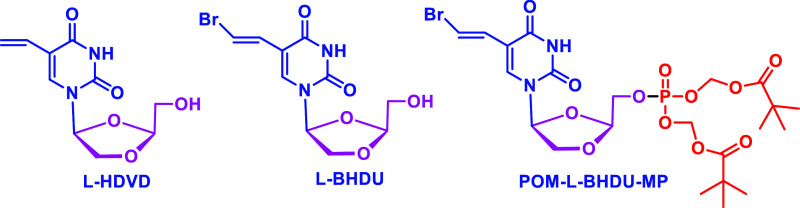
Structures of l-HDVD, l-BHDU, and POM-l-BHDU-MP.

During the past two decades, in search of effective antivirals against VZV, herpes simplex viruses 1 and 2 (HSV 1 & 2), and Epstein–Barr virus (EBV), our group has been involved in the discovery of modified l- and d-dioxolane-nucleos(t)ides. During these efforts, we have discovered 2-(hydroxymethyl)-1,3-dioxolan-4-yl]5-vinyl uracil (l-HDVD, Figure 2)^13^ and 2-bromovinyl-2-(hydroxymethyl)-1,3 dioxolan uracil (l-BHDU)^14,15^ with potent antiviral activity against EBV, VZV & HSV-1. l-HDVD is highly active against EBV (EC_50_ value of 0.01 μM) and Kaposi’s sarcoma-associated herpesvirus (KSHV) (EC_50_ = 0.09 μM).^13^l-BHDU has demonstrated an EC_50_ value of 0.25 μM without cytotoxicity in human foreskin fibroblasts up to 200 μM, with a selectivity index (SI) of >909.^14^ In *in vivo* studies, l-BHDU significantly reduces the viral load in comparison to the ACV and VACV. It is noteworthy that in the metabolic studies, it was found that l-BHDU does not inhibit dihydropyridine dehydrogenase (DPD), and it provided a better safety and antiviral profile than BVDU.^14^

Recently, to increase the bioavailability and uptake of l-BHDU with enhanced pharmacokinetic properties, our group has developed the l-BHDU monophosphate pivaloyloxymethyl ester (POM-l-BHDU-MP) prodrug (Figure 2).^16,17^ POM-l-BHDU-MP has expressed antiviral activity similar to that of l-BHDU in infected cells. In contrast, POM-l-BHDU-MP was superior to l-BHDU in a VZV mouse model.^18^ The pharmacokinetic studies revealed that POM-l-BHDU-MP had a better oral absorption profile compared to l-BHDU.^16^ Repeated assay in humanized mice showed that POM-l-BHDU-MP was effective when administered orally once per day at 11.3 mg/kg or higher, which indicates its potency and bioavailability in vivo.^18^ POM-l-BHDU-MP was evaluated as a topical treatment for VZV and HSV in a human skin explant model, which demonstrated highly effective antiviral activities against both viruses at 0.2% formulated in cocoa butter.^18^ Based on these findings, POM-l-BHDU-MP was selected as a preclinical candidate against VZV infection (shingles).

**Scheme 1:**
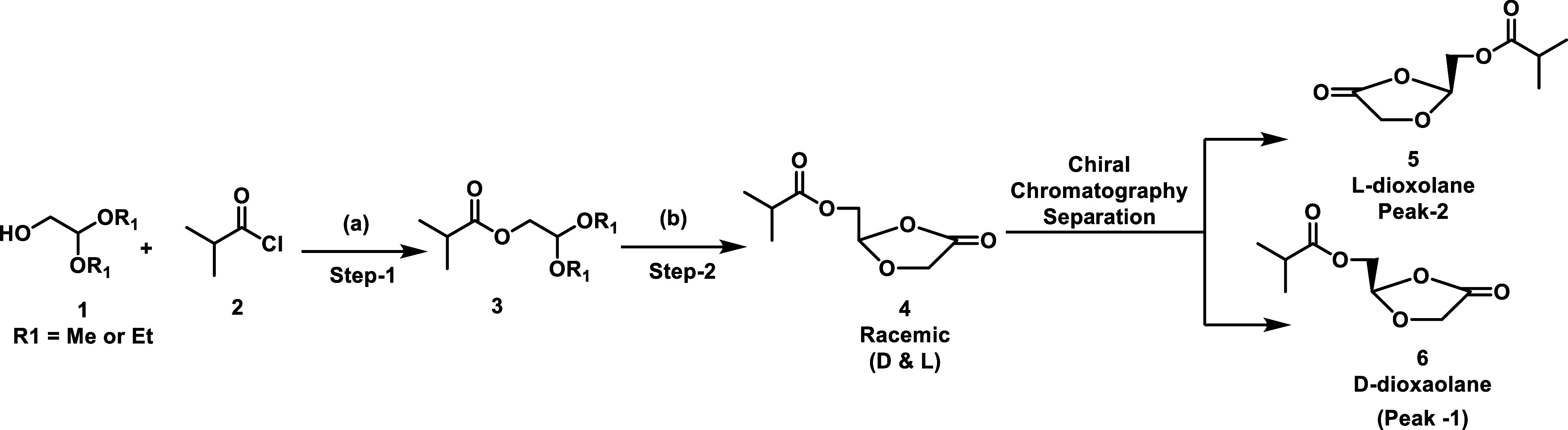
Synthesis of Chiral Pure l- & d-Dioxolane Via Chiral Separation of Racemic Dioxolane **4** Reagents and conditions: (a) DMAP, Et_3_N, *tert*-butylmethyl ether, rt; (b) BF_3_·Et_2_O, acetonitrile 0 °C-rt.

To develop POM-l-BHDU-MP as a preclinical candidate, extended biological, pharmacokinetic, and toxicological studies are required, which demand a significant amount of POM-l-BHDU-MP. Consequently, the development of a robust, practical, and cost-effective synthesis of POM-l-BHDU-MP was needed. Earlier synthesis of l-BHDU was carried out by the l-dioxolane key intermediate **5** (Scheme 1). We and several other research groups followed the procedure reported by Sznaidman et al.^19,20^ for the synthesis of l-dioxane **5**, which is a critical intermediate for the synthesis of l-modified nucleoside analogues.

However, this reported process involved the chiral chromatographic separation of racemic dioxolane **4**, which limits its scalable and commercial utility. Furthermore, during the large-scale synthesis, in the presence of excess boron trifluoride diethyl etherate (BF_3_·Et_2_O), the decomposition of intermediate **4** was observed. Chiral pure l- & d-dioxolane (**5** and **6**) also revealed stability issues during the chiral chromatographic separation, and undesired impurities were observed during the concentration or lyophilization of collected chiral pure fractions. These downsides (Scheme 1) restrict the process for the large-scale synthesis of chiral l- & d-dioxolane (**5** and **6**). To overcome these challenges of Scheme 1, herein we report a scalable, practical synthesis of l-BHDU (**17**) in 7 steps from the methyl (*R*)-2,2-dimethyl-1,3-dioxolane-4-carboxylate **7**. To accomplish the scalable synthesis of l-BHDU (**17**), we revisited synthesis of chiral l- & d-dioxolane, which was previously reported by Bera et al.^21^ Here, we report an improved process for synthesis of both chiral pure l-& d-dioxolane (**11** and **13**, enantiomeric excess ≥99%), which is devoid of chromatography separations and feasible for large-scale synthesis. All previously reported methods described chromatographic and chiral chromatographic separation for the synthesis of the chiral pure l- & d-dioxolane,^19,20^ which limits construction of diverse l- & d-dioxolane-derived nucleosides/nucleotides of pharmaceutical interest. Nevertheless, the present communication discloses a straightforward synthesis and separation of the chiral pure l- & d-dioxolane (**11** and **13**) *via* the formation of a diastereomeric chiral amine salt. The reported procedure is devoid of a chiral separation and reduces column chromatographic steps, which makes the process feasible for the scalable synthesis of the chiral l-dioxolane key intermediate (**11**) and POM-l-BHDU-MP (**24**).

## Results and Discussion

Numerous efforts by various groups to construct modified nucleosides of five-membered dioxolane often lead to a racemic mixture of l- & d-dioxolane and demand chiral SFC purification to obtain the chiral pure l- and d-dioxolane. To avoid the chiral SFC separation, Bera et al.^21^ reported the synthesis of chiral dioxolane *via* methyl-(*R*)-2,2-dimethyl-1,3-dioxalane-4-carboxylate. However, the reported procedure was ambiguous with no analytical data of chiral dioxolanes and required a close analytical technique to confirm the l- & d-enantiomers, which may result in undesired conformational final products. With the l-BHDU (**17**) authentic sample in hand from a previously synthesized method,^22^ we tried to compare the optical value of the newly synthesized l-BHDU (**17**) by the method of Bera et al. with that of our authentic sample without success. Thus, to accomplish the scalable synthesis of l-BHDU (**17**), a new protocol for the synthesis of the chiral pure l-dioxolane was required. The reported method in this communication is entirely focused on the synthesis of the chiral l- & d-dioxolane with an excellent enantiomeric excess (ee ≥ 99%). Notably, this procedure may also be suitable for the large-scale synthesis of the other dioxolane nucleosides such as troxacitabine^23^ and l-HDVD.^13^ It may also expedite the extended structure–activity relationship studies of d- & l-dioxane-derived molecules of therapeutic interest, which have yet to be explored.

**Scheme 2:**
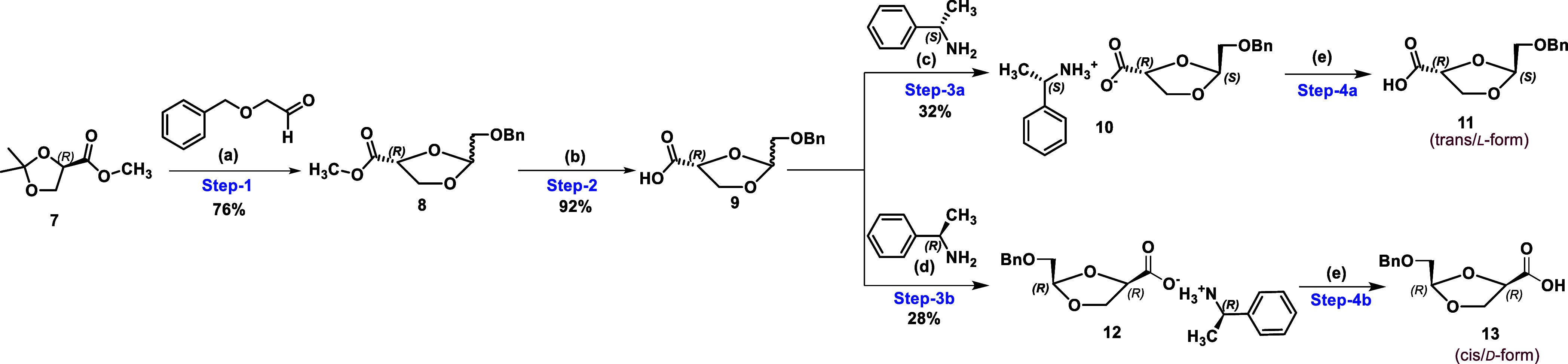
Synthesis of Chiral Pure l- & d-Dioxolane (**11** and **13**) Via Diastereomeric Salt Formation Reagents and conditions:(a) Dowex 50W-X8, toluene 80 °C; (b) 1 M aqueous LiOH, THF, 0 °C-rt; (c) (i) (*S*)-phenylethylamine, acetonitrile 75 °C-rt; (ii) EtOAc/IPA (3:1), rt; (d) (i) (*R*)-phenylethylamine, acetonitrile 75 °C-rt; (ii) EtOAc/IPA (3:1), rt; (e) 1 N aqueous HCl, rt.

The synthesis of chiral pure l-dioxolane key intermediate **11** was initiated by condensation of commercially available methyl (*R*)-2,2-dimethyl-1,3-dioxolane-4-carboxylate **7** with 2-benzyloxyacetaldehyde *via* transketalization in the presence of Dowex 50W X8 to give the racemic 1,3-dioxolane ester **8** in a 1:1 diastereomeric ratio (Scheme 2) in 76% yield. In the earlier reported process, *p*-TSA was utilized to convert **7** to **8**, which reveals hurdles in scale-up and requires a chromatography purification of the product.^21^ Next, hydrolysis of methyl ester **8** was carried out by a 1 M aqueous solution of LiOH in water/THF to afford a diastereomeric mixture of acids **9** in 92% yield.

Next, our goal was to obtain pure l-dioxolane acid **11** with a high enantiomeric purity (ee ≥ 99%) by avoiding the chromatographic separation of racemic acid **9**. However, the selective separation of l-dioxolane was also challenging. One approach was to insert a chiral auxiliary *via* esterification of carboxylic acid **9**, followed by fractional crystallization to give enantiomerically pure l-dioxolane **11**. However, this strategy unnecessarily increases the two-step process of esterification and hydrolysis, which is not appropriate for the large-scale synthesis of l-dioxolane. To reduce the additional steps, it was thought to perform a resolution of racemic **9***via* a diastereomeric salt formation with a chiral amine. It is noteworthy that compound **9** is oily in nature, and other fractional crystallization techniques may not be applicable for the resolution of chiral pure l-dioxolane **11** as well as d-dioxolane **13**. Singh et al. reported chiral resolution of racemic amines *via* chiral pure l-(+)-tartaric acid.^24^ Taking the lead from the reported procedure by Singh et al., we thought to adopt a reverse strategy, which was chiral resolution of racemic acid **9***via* salt formation with a chiral pure amine.

To achieve a diastereomerically pure salt formation of racemic **9**, several (*R*) or (*S*) chiral amines were utilized (Table 1) in various solvent systems. In the investigational resolution of racemic acid **9**, diastereomeric salt formation of **9** with (*S*)-phenylethylamine produced pure chiral dioxolane salt **10** with a diastereomeric excess (de) of 99.37% (entry 4 in Table 1). Racemic acid **9** was taken in acetonitrile and treated with 0.8 eq. of (*S*)-phenylethylamine, which gives a white salt of the acid amine after precipitation as l-dioxolane salt **10**. Our next goal was to determine the chiral purity of the precipitated salt as well as the stereo conformation of the diastereomeric salt. The precipitated diastereomeric salt **10** was treated with the 1 N aq. HCl to afford free diastereomeric l-dioxolane **11**. The chiral purity of the obtained free l-dioxolane **11** was determined by chiral HPLC, which indicates that the optical purity of the precipitated isomers is 85% with a 15% presence of another isomer. The obtained results of diastereomeric salt formation *via* resolution of racemic **9** were not encouraging because, for the construction of optically pure l-BHDU (**17**), there was a need for l-dioxolane **11** with an optical purity of more than 99% (ee ≥ 99%).

**Table 1:** Diastereomeric Salt Formation Results from Racemic Compound **9** with Various Chiral Amines

entry	chiral amine	solvent	temp.	time h	result	yield
1.	(*R*)-phenylethylamine	EtOAc/IPA (2:0.5)	rt	4	salt contains both d & l isomers in 1:1(de ∼ 50%)	60%
2.	(*R*)-phenylethylamine	EtOAc/IPA (2:1)	45 °C to rt	4	salt contains both d & l isomers in 1:0.5 (de ∼ 60%)	70%
3.	(*R*)-phenylethylamine	acetonitrile	70 °C to rt	4	salt contains both d (82%) & l isomers (18%) in 1:1(de ∼ 64%)	65%
4.	(*S*)-phenylethylamine	acetonitrile (Step-1)	70 °C to rt	4	salt contains both d- (15%) & l-isomers (85%) (de ≥ 70%)	32% after both step 1 and 2
		obtained salt from step-1 was resuspended in EtOAc/IPA(3:1) (Step-2)	rt	4	yielded salt contains l-isomer (de ≥ 99%)	
5.	(*R*)-phenylethylamine	acetonitrile(Step-1)	70 °C to rt	4	salt contains both d- (83%) & l-isomer (17%) (de ≥ 66%)	28% after both step 1 and 2
		obtained salt from step-1 was resuspended in EtOAc/IPA (3:1) (Step-2)	rt	4	yielded salt contains d-isomer (de ≥ 99%)	
6.	(*S*)-phenylethylamine	EtOAc/IPA (1:2)	60 °C to rt	4	salt formation was observed but was moisture sensitive	NA
7.	(*S*)-phenylethylamine	EtOAc/IPA (1:1)	60 °C to rt	4	salt formation was observed but was moisture sensitive	NA
8.	(*S*)-phenylethylamine	EtOA/IPA 3:1	rt	6	yielded salt contains l-isomer (de ≥ 90.0%)	55%
9.	(*S*)-(+)-1-(1-naphthyl) ethylamine	acetonitrile	70 °C to rt	78	no salt formation	NA
10.	*(R*)-(+)-1-(1-naphthyl) ethylamine	acetonitrile	70 °C to rt	16	no salt formation	NA
11.	(*S*)-(−)-*N*-benzyl-1-phenylethylamine	acetonitrile	70 °C to rt	32	no salt formation	NA

**Figure 3:**
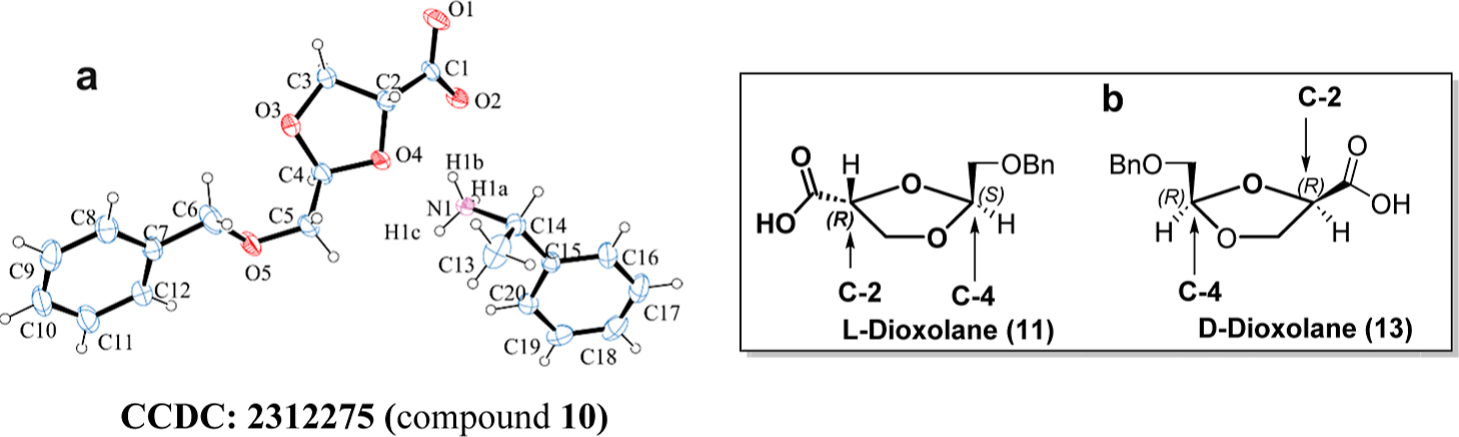
(**3a**) ORTEP diagram of compound **10** confirms the relative stereochemistry of l-dioxolane; (**3b**) Stereo structure of l- and d-dioxolane; in the case of l-dioxolane, C-2 and C-4 hydrogen atoms are trans (*RS*), whereas in d-dioxolane, C-2 and C-4 hydrogen are cis (*RR*) to each other.

Hence, the process of the diastereomeric salt formation of racemic compound **9** was revisited. To enrich the diastereomeric excess (de) of the precipitated salt (*via* acetonitrile), the obtained salt **10** was resuspended in EtOAc/isopropanol (IPA) [3:1] and stirred for 4 h at room temperature, then filtered (filtrate contains a racemic mixture of major d- and minor l-dioxolane) and neutralized by 1 N aqueous HCl. The afforded l-dioxolane **11** was re-examined for chiral purity *via* chiral HPLC, which demonstrated an ee of more than 99% (ee = 99.18%) in 32% yield. Therefore, it was concluded that treatment of a double solvent system, first in acetonitrile followed by EtOAc/IPA (3:1), enhances the diastereomeric excess (de) of the salt precipitate (**10**, de ≥ 99.3%). The subsequent objective was the stereo conformation determination of the precipitated salt, either the d- or l-form of dioxolane. The X-ray crystal of the precipitated salt was developed, and it confirmed that the formed diastereomeric salt matches with the stereo conformation of l-dioxolane **10**. The X-ray structure reveals that hydrogen atoms present at carbon-2 (C-2) and C-4 are trans (Figure 3b) to each other, which confirms l-dioxolane salt precipitation.

However, a single solvent salt formation approach was also attempted with various chiral amines mentioned in Table 1, but in each case, the results of de were unsatisfactory. To improve the yield of intermediate **11**, altered ratios of EtOAc & IPA were used, but in all efforts, either less yield or less chiral purity of intermediate **11** was achieved.

Encouraged by the above finding, it was worth examining the d-dioxolane salt **12** precipitation *via* racemic **9**. It was predicted that by application of chiral (*R*)-phenylethyl amine [inverse conformation of (*S*)-phenylamine)] it may provide the diastereomeric salt of d-dioxolane. Similarly, racemic acid **9** was taken in acetonitrile and treated with 0.8 eq. of (*R*)-phenylethylamine, which gives a white salt of the acid amine after precipitation as d-dioxolane salt **12**. Furthermore, d-dioxolane salt **12** was resuspended in EtOAc/IPA (3:1) and stirred for 4 h at room temperature, and then it was filtered and neutralized by 1 N aqueous HCl solution to afford d-dioxolane **13** in 28% yield with ee ≥ 99.3% (entry 5, Table 1). The direct precipitation of chiral salts of l- & d-dioxolane by the racemic **9** is a crucial finding, and the described method may expedite the synthesis of numerous novel derivatives of chiral pure l- and d-dioxolane-derived small molecules.

**Scheme 3:**
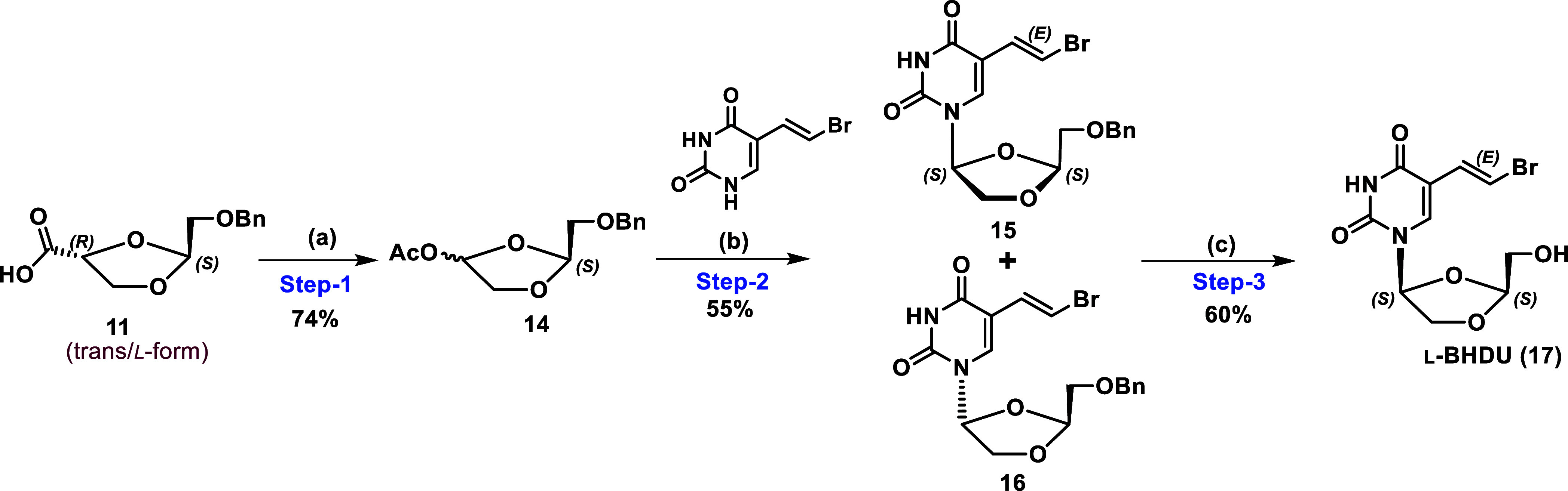
Synthesis of l-BHDU Via l-Dioxolane Intermediate (**11**) (a) Pb(OAc)_4_, pyridine, acetonitrile, 0 °C–rt; (b) BVU, TBDMSOTf, TMSI, DCM, rt; (c) BCl_3_, DCM, −78 °C.

To achieve the synthesis of chiral l-BHDU, compound **11** served as the key intermediate. Treatment of **11** with lead(IV) acetate [Pb(OAc)_4_], in the presence of pyridine in acetonitrile, converts the 2-carboxyl group of **11** to 2-acetoxy intermediate **14** in an α/β ratio of 2:1 (racemic) in 74% yield. Furthermore, glycosylation of **14** with bromovinyl-uracil (BVU) was carried out under the Vorbrüggen coupling condition (Scheme 3).^25^

The bromouracil base was treated with trimethylsilyl trifluoromethanesulfonate (TMSOTf) followed by (iodotrimethylsilane) TMSI, which in situ generates silylated BVU and condenses with acetate intermediate **14** (α/β ratio 2:1) to give coupled β and α isomer of **15** and **16** in ratio of 3:1. Separation of isomers by column chromatography afforded the desired β-isomer **15** in 55% yield and α-isomer **16** in 18% yield. Finally, deprotection of 5′-benzyl of coupled product **15** was performed using a 1 M solution of boron trichloride (BCl_3_) in DCM to generate the targeted compound l-BHDU (**17**, ee ≥ 99%) in 60% yield.

**Scheme 4:**
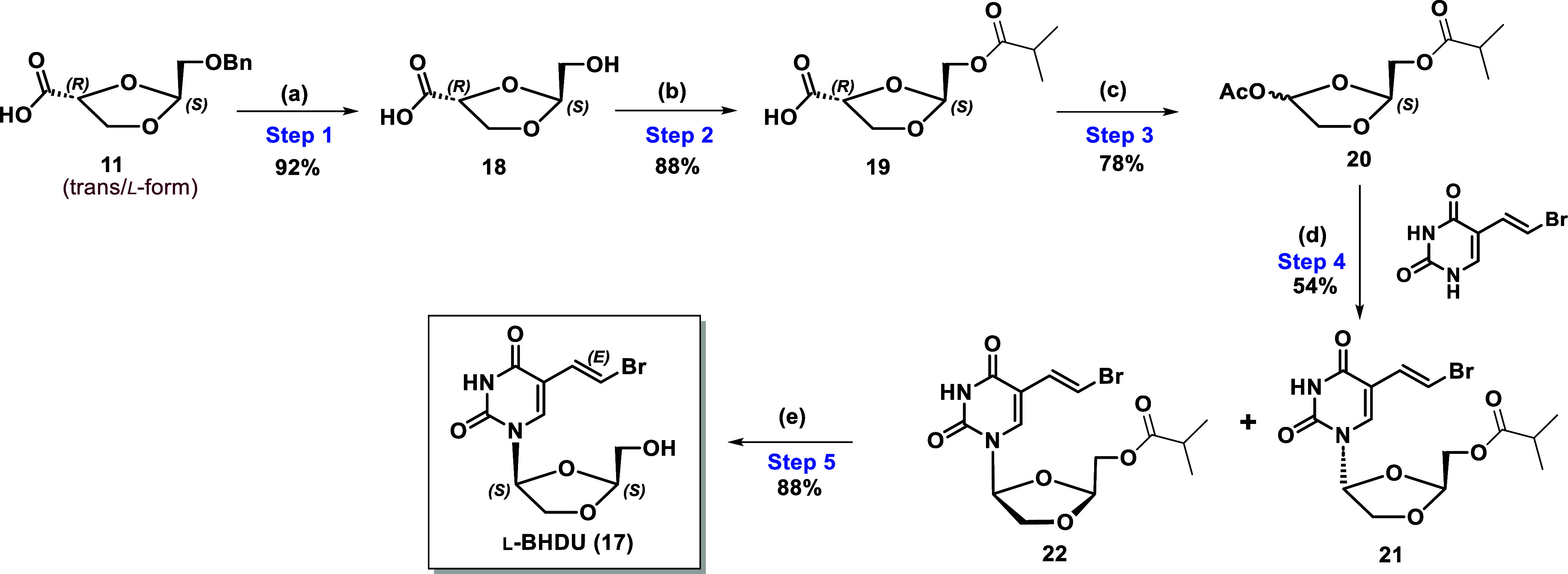
Revised Synthesis of l-BHDU (**17**) Via Intermediate **11** Reagents and conditions: (a) 5% Pd/C, H_2_ gas, MeOH, r.t.; (b) Isobutyric anhydride, pyridine, DMAP, DCM, 0 °C–rt; (c) Pb(OAc)_4_, pyridine, acetonitrile, 0 °C-rt.; (d) BVU, TBDMSOTf, TMSI, DCM, rt.; (e) 7 N NH_3_ solution in methanol, rt.

However, the exothermic final deprotection of the benzyl group by using BCl_3_ was not suitable for large-scale synthesis. To avoid the use of BCl_3_, a protection group replacement strategy was adopted, and 5-*O*-benzyl of intermediate **11** was replaced with an isobutyric ester. The debenzylation of **11** was carried out with 5% Pd/C under hydrogenation conditions to afford intermediate **18** in 91% yield. Intermediate **18** was treated with isobutyric anhydride in the presence of pyridine to produce **19** in 88% yield. The acylation of compound **19** followed by the coupling with BVU afforded coupled desired (β) nucleoside **22** (54% yield) and undesired (α) nucleoside **21** (yield 22%) in an approximately 2:1 ratio (Scheme 4).

Deprotection of the isobutyl ester of **22** was executed by a 7 N NH_3_ solution in MeOH to give l-BHDU (**17**) in 88% yield. Optical rotation and other analytical data of **17** were consistent with previously synthesized authentic l-BHDU.^22^

**Scheme 5:**
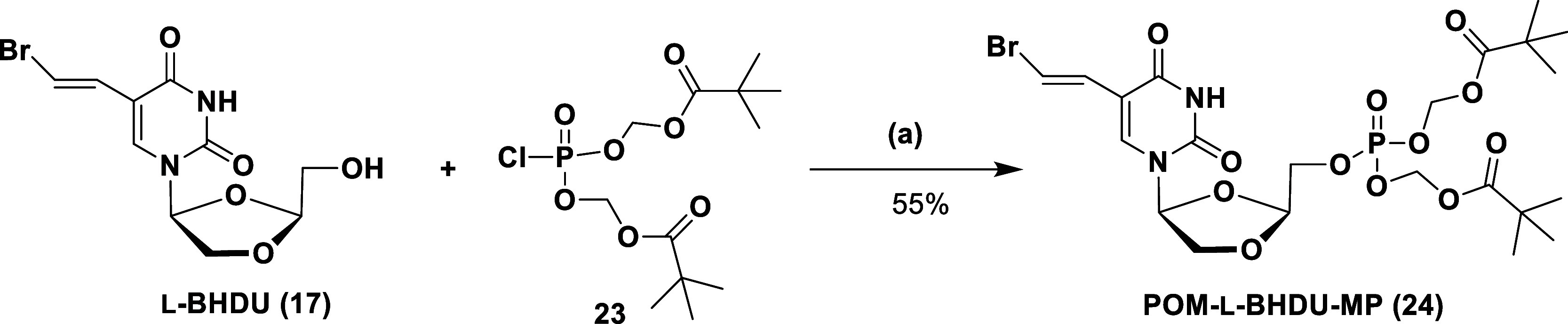
Synthesis of the POM-l-BHDU-MP Prodrug Via l-BHDU (**17**) Reagents and conditions: (a) NMI, THF, 0 °C to rt, 3 h.

To synthesize the POM-l-BHDU-MP prodrug, first synthesis of bis(POM)phosphorochloridate (**23**) was carried out according to the reported protocol by Hawang and Cole.^26^ Next, coupling of l-BHDU (**17**) was performed with POM chloride (**23**) in the presence of *N*-methyl imidazole in THF to furnish POM-l-BHDU-MP (**24**) in 55% yield (Scheme 5).^16^

## Conclusions

To determine the full biological profile of POM-l-BHDU-MP (**24**), an efficient and scalable synthetic method of l-BHDU (**17**) has been developed *via* commercially available methyl (*R*)-2,2-dimethyl-1,3-dioxolane-4-carboxylate, **7**. The selective diastereomeric salt formation of racemic **9***via* a chiral (*S*)-phenyl ethylamine followed by neutralization of **10** gave optically pure l-dioxolane, **11** (ee ≥ 99%). Compound **11** was converted to acetate intermediate **14**, which was utilized for Vorbrüggen coupling with BVU followed by the benzyl deprotection of coupled nucleoside to afford the target compound **17** (l-BHDU) in 7 steps with approximately 4.9% overall yield. This process removes the expensive chiral separation of racemic l- & d-dioxolane and is more efficient than the previously reported method for the synthesis of l-BHDU (**17**). Further coupling of bis(POM)phosphorochloridate **23** with l-BHDU (**17**) produced l-BHDU-monophosphate ester prodrug **24** (POM-l-BHDU-MP). The removal of chiral separation and reduction of column chromatography in synthetic steps and the use of economical reagents make the reported methodology amenable for large-scale preparation of POM-l-BHDU-MP (**24**). Additionally, the reported synthesis of chiral pure l- & d-dioxolane (**11** and **13**) opens new paths for the synthesis of diverse nucleoside/nucleotide analogues of pharmaceutical interest.

## Experimental Section

### General Analytical Methods

Reagents and anhydrous solvents were purchased from commercial sources and used without further purification. Moisture-sensitive reactions were performed by using oven-dried glassware under a nitrogen or argon atmosphere. Reactions were monitored by thin-layer chromatography plates (TLC silica gel GF 250 μm) that were visualized using a Spectroline UV lamp (254 nm) and developed with 15% solution of sulfuric acid in methanol. Column chromatography was performed on silica gel 60 Å, 40–63 μM (230 × 400 mesh, Sorbent Technologies). Preparative normal phase chromatography was performed on a CombiFlash Rf 150 (Teledyne Isco) with prepacked RediSep Rf silica gel cartridges or on RediSep gold C18 reverse phase columns. Melting points were recorded on a Mel-temp II laboratory device and are uncorrected. Nuclear magnetic spectra were recorded on Varian Inova 500 spectrometer at 500 MHz for ^1^H NMR, 202 MHz for ^31^P NMR, and 125 MHz for ^13^C NMR with tetramethylsilane as an internal standard. Chemical shifts (δ) are quoted as s (singlet), bs (broad singlet), d (doublet), t (triplet), q (quartet), m (multiplet), dd (double doublet), and dt (double triplet). Optical rotations were measured on a JASCO DIP-370 digital polarimeter. Structural assignments were determined using additional information from gCOSY, gHSQC, and gHMBC experiments. High-resolution mass spectroscopy (HRMS) spectra were recorded on a Bruker Ultrahigh resolution QTOF MS Impact II spectrometer. Samples were infused at 3 μL/min, and spectra were obtained in the positive or negative ionization mode with a typical resolution of 20,000 or greater. Optical purity of chiral intermediates and final chiral compounds were determined by the chiral HPLC. Chiral HPLC/UV were performed with a Waters HPLC coupled to a photodiode array. Ten microliters of samples (0.5 mg/mL in methanol) were injected using a CHIRALCEL OX-H, 5 μmm (4.6 × 250 mm) column at 30 °C with a flow rate of 3.0 mL/min.

#### Methyl (4*R*)-2-((Benzyloxy)methyl)-1,3-dioxolane-4-carboxylate (**8**)

To a solution of 2-(benzyloxy)acetaldehyde (100 g, 665.91 mmol) and methyl (*R*)-2,2-dimethyl-1,3-dioxolane-4-carboxylate **7** (106.65 g, 665.91 mmol) in toluene (700 mL), Dowex 50W X8 (2.8 g) was added, and the mixture was stirred at 80 °C in an oil bath for 2 h. The reaction mixture was cooled to room temperature, filtered, and the volatiles were evaporated under reduced pressure. The crude mixture was purified by flash silica gel column chromatography with 12–18% EtOAc/hexane as eluent to obtain an inseparable diastereomeric mixture of carboxylate **8** as light-yellow oil. Yield (128.0 g, 76%); ^1^H NMR (500 MHz, CDCl_3_): δ 7.37–7.32 (m, 11H), 7.30–7.27 (m, 2H), 5.33 (t, *J* = 4.0 Hz, 1H), 5.22 (t, *J* = 4.5 Hz, 1H), 4.68–4.58 (m, 7H), 4.31 (dd, *J* = 8.2 and 7.3 Hz, 1H), 4.24 (dd, *J* = 8.6 and 3.9 Hz, 1H), 4.13–4.09 (m, 1H), 3.99 (dd, *J* = 8.3 and 5.4 Hz, 1H), 3.78 (s, 3H), 3.75 (s, 3H), 3.71 (d, *J* = 3.8 Hz, 1H), 3.67–3.61 (m, 2H), 3.59 (d, *J* = 4.1 Hz, 1H); ^13^C{^1^H}NMR (126 MHz, CDCl_3_): δ 171.3, 171.1, 138.0, 137.9, 128.5, 128.4, 127.9, 127.83, 127.81, 127.8, 104.9, 104.3, 74.1, 73.9, 73.8, 73.7, 70.9, 70.3, 68.7, 68.3, 52.5; HRMS-ESI (*m*/*z*): [M + Na]^+^ calcd for [C_13_H_16_O_5_Na]^+^, 275.0895; found, 275.0883.

#### (4*R*)-2-((Benzyloxy)methyl)-1,3-dioxolane-4-carboxylic Acid (**9**)

To a solution of **8** (60 g, 237.84 mmol) in THF (250 mL) at 0 °C was dropwise added 1 M aqueous solution of LiOH (198 mL), and the mixture was slowly warmed to room temperature and stirred for 36 h. THF was evaporated under reduced pressure, and mixture was washed with EtOAc (3 × 75 mL). The separated aqueous layer was cooled to 0 °C and acidified with 28% aq. HCl to pH ∼ 3 and extracted with DCM (3 × 125 mL). The combined organic layer was washed with brine, dried over Na_2_SO_4_, filtered, and concentrated under reduced pressure to obtain racemic acid **9** as a light brown thick liquid. Yield (52.5 g, 92%); ^1^H NMR (500 MHz, CDCl_3_): δ 7.40–7.32 (m, 10H), 7.31–7.27 (m, 1H), 5.34 (t, *J* = 3.6 Hz, 1H), 5.20 (s, 1H), 4.74 (d, *J* = 12.1 Hz, 1H), 4.70–4.64 (m, 3H), 4.61 (s, 2H), 4.42–4.34 (m, 2H), 4.15–4.09 (m, 2H), 4.04 (dd, *J* = 8.4 and 5.4 Hz, 1H), 3.80 (dd, *J* = 11.4 and 1.7 Hz, 1H), 3.76 (d, *J* = 11.4 Hz, 1H), 3.61 (dd, *J* = 5.9 and 3.6 Hz, 2H); ^13^C{^1^H} NMR (126 MHz, CDCl_3_): δ 174.8, 173.6, 137.68, 136.0, 128.8, 128.5, 128.3, 127.9, 127.8, 104.4, 104.2, 74.3, 73.8, 73.6, 70.5, 70.2, 68.3, 67.5; HRMS-ESI (*m*/*z*): [M + Na]^+^ calcd for [C_12_H_14_O_5_Na]^+^, 261.0739; found, 261.0728.

#### (2*S*,4*R*)-2-((Benzyloxy)methyl)-1,3-dioxolane-4-carboxylate-(*S*)-1-phenylethan-1-aminium (**10**)

A solution of racemic acid **9** (25 g, 105.0 mmol) in ACN (50 mL) was heated to 70 °C in an oil bath for 20 min. After that, (*S*)-(−)-1-phenylethylamine (10.80 g, 89.25 mmol) was added dropwise. The mixture was stirred at 70 °C in an oil bath for 2 h and then slowly cooled to room temperature and stirred for 3 h to give a white solid precipitate (contains major l-dioxolane), and then it was filtered. To remove the undesired d-dioxolane salt (15%) from the precipitate, the filtered solid was resuspended in a mixture of EtOAc/isopropanol (50 mL, 3:1) and stirred for 4 h at rt and filtered. The diastereomeric purity of **10** was monitored by NMR, and the process was repeated 3 times (the undesired isomer d-dioxolane salt was completely removed from the solid precipitate by washing with mixture of EtOAc/isopropanol, 3:1) to obtain compound **10** as a white amorphous solid. Yield (12.1 g, 32%); ^1^H NMR (500 MHz, CDCl_3_): δ 8.37 (bs, 3H), 7.35–7.25 (m, 10H), 5.03 (t, *J* = 3.9 Hz, 1H), 4.57–4.50 (m, 2H), 4.27–4.21 (m, 2H), 4.04 (t, *J* = 7.7 Hz, 1H), 3.63 (t, *J* = 7.1 Hz, 1H), 3.46–3.43 (m, 2H), 1.49 (d, *J* = 6.8 Hz, 3H); ^13^C{^1^H} NMR (126 MHz, CDCl_3_): δ 176.7, 139.3, 137.8, 128.9, 128.5, 128.4, 127.9, 127.86, 126.6, 103.0, 75.7, 73.4, 70.8, 68.7, 50.9, 21.1.

#### (2*S*,4*R*)-2-((Benzyloxy)methyl)-1,3-dioxolane-4-carboxylic Acid (**11**)

Compound **10** (11.2 g, 31.18 mmol) was dissolved in DCM (200 mL) and sequentially washed with cold 1 N aqueous HCl (20 mL), water (100 mL), and finally with brine (100 mL). The organic layer was dried over Na_2_SO_4_, filtered, and concentrated under reduced pressure to give acid **11** as a thick, colorless liquid. Yield (7.1 g, 95%, ee = 99.18%); [α]_D_^25^ = +20.82 (c 0.5, CHCl_3_); ^1^H NMR (500 MHz, CDCl_3_): δ 7.61 (bs, 1H), 7.36–7.31 (m, 4H), 7.30–7.25 (m, 1H), 5.32 (t, *J* = 3.5 Hz, 1H), 4.69–4.66 (m, 1H), 4.60 (s, 2H), 4.34 (t, *J* = 7.9 Hz, 1H), 4.02 (dd, *J* = 8.4 and 5.4 Hz, 1H), 3.60 (dd, *J* = 5.9 and 3.6 Hz, 2H); ^13^C{^1^H} NMR (126 MHz, CDCl_3_): δ 175.5, 137.7, 128.5, 128.0, 127.9, 104.5, 73.9, 73.7, 70.2, 68.3; HRMS-ESI (*m*/*z*): [M + Na]^+^ calcd for [C_12_H_14_O_5_Na]^+^, 261.0739; found, 261.0729.

#### (2*R*,4*R*)-2-((Benzyloxy)methyl)-1,3-dioxolane-4-carboxylate-(*R*)-1-phenylethan-1-aminium (**12**)

A solution of racemic **9** (5 g, 20.98 mmol) in ACN (8 mL) was heated to 70 °C in an oil bath for 20 min. After that, (*R*)-(−)-1-phenylethylamine (2.16 g, 17.83 mmol) was added dropwise. The mixture was stirred at 70 °C in an oil bath for 2 h, then slowly cooled to room temperature, and stirred for 3 h to give a white solid precipitate. The obtained precipitate was filtered to remove the undesired l-dioxolane salt (∼20%) from the precipitate, and the filtered solid was resuspended in a mixture of EtOAc/isopropanol (15 mL, 3:1) and stirred for 2 h at rt and filtered. The diastereomeric purity of **12** was monitored by NMR, and the process was repeated 3 times (the undesired isomer l-dioxolane salt was completely removed from the solid precipitate by washing with mixture of EtOAc/isopropanol, 3:1) to obtain compound **12** as a white amorphous solid. Yield (2.1 g, 28%); ^1^H NMR (500 MHz, CDCl_3_): δ 7.36 (d, *J* = 7.0 Hz, 2H), 7.33–7.28 (m, 5H), 7.24 (s, 1H), 5.06 (t, *J* = 3.7 Hz, 1H), 4.46 (s, 2H), 4.26–4.4.17 (m, 2H), 3.95 (q, *J* = 6.1 and 10.7 Hz, 1H), 3.80 (q, *J* = 5.4 and 6.6 Hz, 1H), 3.52–3.46 (m, 2H), 1.50 (d, *J* = 6.8 Hz, 3H); ^13^C{^1^H} NMR (126 MHz, CDCl_3_): δ 176.7, 139.3, 137.8, 128.9, 128.5, 128.4, 127.9, 127.9, 126.6, 103.0, 75.7, 73.4, 70.8, 68.7, 50.9, 21.1.

#### (2*R*,4*R*)-2-((Benzyloxy)methyl)-1,3-dioxolane-4-carboxylic Acid (**13**)

Compound **12** (2.0 g, 5.56 mmol) was dissolved in DCM (15 mL) and sequentially washed with cold 1 N aqueous HCl (10 mL), water (10 mL), and finally brine (10 mL). The organic layer was dried over Na_2_SO_4_, filtered, and concentrated under reduced pressure to give **13** as a thick, colorless liquid. Yield (1.2 g, 96%, ee = 99.34); [α]_D_^25^ = −17.25 (c 0.5, CHCl_3_); ^1^H NMR (500 MHz, CDCl_3_): δ 7.39–7.32 (m, 5H), 5.20 (s, 1H), 4.74 (d, *J* = 12.1 Hz, 1H), 4.69–4.63 (m, 2H), 4.39 (d, *J* = 9.3 Hz, 1H), 4.13–4.08 (m, 1H), 3.81 (d, *J* = 11.5 Hz, 1H), 3.76 (d, *J* = 11.5 Hz, 1H); ^13^C{^1^H} NMR (126 MHz, CDCl_3_): δ 173.2. 136.1, 128.8, 128.5, 128.3, 104.2, 74.3, 70.5, 67.5; HRMS-ESI (*m*/*z*): [M + Na]^+^ calcd for [C_12_H_14_O_5_Na]^+^, 261.0739; found, 261.0742.

#### (2*S*)-2-((Benzyloxy)methyl)-1,3-dioxolan-4-yl Acetate (**14**)

To a stirred solution of compound **11** (2.0 g, 8.39 mmol) and anhydrous pyridine (0.95 mL, 12.08 mmol) in acetonitrile (15 mL) was added Pb(OAc)_4_ (4.46 g, 1.48 mmol) portionwise at 0 °C and stirred for 16 h at rt. The reaction mixture was filtered through a Celite bed. The obtained filtrate was diluted with EtOAc (60 mL) and washed sequentially with saturated aqueous solution of NaHCO_3_ (25 mL), water (25 mL), and finally with brine (30 mL). The organic layer was dried over Na_2_SO_4_, filtered, and concentrated under reduced pressure. The crude was purified by flash silica gel column chromatography using 15–18% EtOAc/Hexane as eluent to obtain a diastereomeric mixture of **14** as a colorless liquid. Yield (1.35 g, 74%); ^1^H NMR (500 MHz, CDCl_3_): δ 7.37–7.27 (m, 8H), 6.39 (dd, *J* = 4.4 and 2.1 Hz, 1H), 6.33 (d, *J* = 3.7 Hz, 0.6H), 5.38–5.36 (m, 1H), 5.30–5.27 (m, 0.7H), 4.64–4.59 (m, 3H), 4.23 (dd, *J* = 9.5 and 4.7 Hz, 1H), 4.18 (d, *J* = 9.6 Hz, 0.6H), 3.99–3.93 (m, 2H), 3.67–3.57 (m, 4H), 2.09 (s, 3H), 2.03 (s, 2H); ^13^C{^1^H} NMR (126 MHz, CDCl_3_): δ 170.4, 137.8, 128.5, 127.8, 105.3, 103.8, 94.6, 94.1, 73.8, 73.8, 73.7, 71.4, 71.1, 70.9, 70.2, 21.2; HRMS-ESI (*m*/*z*): [M + Na]^+^ calcd for [C_13_H_16_O_5_Na]^+^, 275.0895; found, 275.0884.

#### 1-((2*S*,4*S*)-2-((Benzyloxy)methyl)-1,3-dioxolan-4-yl)-5-((*E*)-2-bromovinyl)pyrimidine-2,4(1*H*,3*H*)-dione (**15**)

To a suspension of (*E*)-5-bromovinyluracil (500 mg, 2.30 mmol) in dry DCM (15 mL) were added TBDMSOTf (1.37 mL, 5.99 mmol) and 2,4,6-collidine (0.97 mL, 5.99 mmol) at rt, and the mixture was stirred for 30 min. To the resulting solution was added slowly a solution of **14** (580 mg, 2.30 mmol) in DCM (20 mL) dropwise, followed by the addition of TMSI (0.36 mL, 2.53 mmol). The mixture was stirred at room temperature for 3 h and quenched with a saturated aqueous solution of Na_2_S_2_O_3_ (20 mL). The organic layer was washed with brine (15 mL), dried over Na_2_SO_4_, filtered, and concentrated under reduced pressure. The crude product was purified by silica gel column chromatography (1:4 EtOAc/Hexane as eluent) to give undesired α-isomer **16** (185 mg, 18%) and desired β -isomer **15** (525 mg, 55%) as a white solid. ^1^H NMR (500 MHz, CDCl_3_): δ 8.27 (bs, 1H), 7.91 (s,1H), 7.40–7.29 (m, 6H), 6.37–6.31 (m, 2H), 5.12 (s, 1H), 4.69–4.61 (m, 2H), 4.23 (d, *J* = 10.1 Hz, 1H), 4.15 (dd, *J* = 10.2 and 5.3 Hz, 1H), 3.84 (dd, *J* = 5.6 and 1.8 Hz, 2H); ^13^C{^1^H} NMR (126 MHz, CDCl_3_): δ 160.8, 149.3, 138.0, 136.9, 128.8, 128.5, 128.1, 127.9, 111.6, 110.0, 104.9, 81.4, 74.4, 71.9, 67.9; HRMS-ESI (*m*/*z*): [M + Na]^+^ calcd for [C_17_H_17_BrN_2_O_5_Na]^+^, 431.0219; found, 431.0207.

#### 5-((*E*)-2-Bromovinyl)-1-((2*S*,4*S*)-2-(hydroxymethyl)-1,3-dioxolan-4-yl)pyrimidine-2,4(1*H*,3*H*)-dione (17)

To a stirred solution of compound **15** (500 mg, 1.22 mmol) in DCM (2 mL) at −78 °C, 1 M BCl_3_ in DCM (1.34 mL, 1.34 mmol) was added slowly dropwise and stirred at the same temperature for 30 min. After that, the reaction mixture was quenched with MeOH (1 mL) and warmed to room temperature, and the inorganic salts were filtered. The organic layer was concentrated under reduced pressure. The crude product was purified *via* silica gel column chromatography (DCM/MeOH, 9:1) to give L-BHDU (**17**, 235 mg, 60%) as a white solid. [α]_D_^23^ = −6.2 (c 0.2.7, MeOH); ^1^H NMR (500 MHz, DMSO-*d*_6_): δ 11.61 (bs, 1H), 8.16 (s, 1H), 7.22 (d, *J* = 13.6 Hz, 1H), 6.82 (d, *J* = 13.6 Hz, 1H), 6.21 (d, *J* = 5.0 Hz, 1H), 5.31 (t, *J* = 6.0 Hz, 1H), 4.97–4.94 (m, 1H), 4.31 (d, *J* = 9.9 Hz, 1H), 4.09 (dd, *J* = 9.9 and 5.6 Hz, 1H), 3.74–3.67 (m, 2H); ^13^C{^1^H} NMR (126 MHz, DMSO-*d*_6_): δ 162.2, 150.1, 140.1, 130.3, 110.2, 107.0, 105.8, 81.1, 71.1, 60.4; HRMS-ESI (*m*/*z*): [M + Na]^+^ calcd for [C_10_H_11_BrN_2_O_5_Na]^+^, 340.9749; found, 340.9738.

#### (2*S*)-2-((Benzyloxy)methyl)-1,3-dioxolan-4-yl Acetate (**18**)

A suspension of compound **11** (3.0 g, 12.60 mmol) and 5% Pd/C (150 mg) in MeOH (20 mL) at ambient temperature was treated with hydrogen at 8 psi in a Parr hydrogenator for 3 h. The mixture was diluted with methanol (20 mL) and passed through a Celite bed. The filtrate was concentrated under reduced pressure to obtain **18** as a colorless thick liquid. Yield (1.72 g, 92%); [α]_D_^26^ = +16.21 (c 0.5, MeOH); ^1^H NMR (500 MHz, CD_3_OD): δ 5.08 (t, *J* = 3.2 Hz, 1H), 4.60 (dd, *J* = 5.5 and 7.0 Hz, 1H), 4.24 (t, *J* = 7.8 Hz, 1H), 3.90 (dd, *J* = 5.3 and 8.2 Hz, 1H), 3.54 (d, *J* = 3.0 Hz, 2H); ^13^C{^1^H} NMR (126 MHz, CD_3_OD): δ 173.3, 104.9, 73.9, 68.0, 62.0; HRMS-ESI (*m*/*z*): [M – H]^−^ calcd for [C_5_H_7_O_5_]^−^, 147.0293; found, 147.0301.

#### (2*S*,4*R*)-2-((Isobutyryloxy)methyl)-1,3-dioxolane-4-carboxylic Acid (**19**)

To a solution of compound **18** (2.0 g, 13.50 mmol) in dry pyridine (15 mL) at 0 °C was added isobutyric anhydride (3.2 mL, 20.26 mmol) followed by DMAP (8 mg), and the mixture was stirred at rt for 16 h. The reaction mixture was diluted with DCM (60 mL) and washed with water (2 × 25 mL) and finally with brine (25 mL). The organic layer was dried over Na_2_SO_4_, filtered, and concentrated under reduced pressure. The crude was purified by flash silica gel column chromatography using 60–65% EtOAc/Hexane as the eluent to give **19** as a colorless liquid. Yield (2.60 g, 88%); [α]_D_^26^ = +23.26 (c 0.5, CHCl_3_); ^1^H NMR (500 MHz, CDCl_3_): δ 5.38 (t, *J* = 3.7 Hz, 1H), 4.67 (dd, *J* = 5.1 and 7.1 Hz, 1H), 4.30–4.25 (m, 1H), 4.18 (t, *J* = 3.6 Hz, 2H), 4.02 (dd, *J* = 5.1 and 8.4 Hz, 1H), 2.60 (p, *J* = 7.0 Hz, 1H), 1.18 (d, *J* = 7.0 Hz, 6H); ^13^C{^1^H} NMR (126 MHz, CDCl_3_): δ 170.8, 176.7, 103.4, 74.1, 68.7, 64.0, 33.9, 19.0; HRMS-ESI (*m*/*z*): [M + Na]^+^ calcd for [C_9_H_14_O_6_Na], 241.0688; found, 241.0673.

#### (2*S*)-2-((Benzyloxy)methyl)-1,3-dioxolan-4-yl Acetate (**20**)

To a stirred solution of compound **19** (1.5 g, 6.87 mmol) and dry pyridine (0.78 mL, 9.90 mmol) in acetonitrile, Pb(OAc)_4_ (3.80 g, 8.58 mmol) was added portionwise at 0 °C and stirred for 16 h at rt. After that, the reaction mixture was passed through a Celite bed. The filtrate was diluted with EtOAc (100 mL) and washed with water (50 mL) and finally with brine (50 mL). The organic layer was dried over Na_2_SO_4_, filtered, and concentrated under reduced pressure. The crude product was purified by flash silica gel column chromatography (12–16% EtOAc/Hexane) to give **20** as a colorless liquid. Yield (1.23 g, 78%); ^1^H NMR (500 MHz, CDCl_3_) (mix of diastereomers, 2:1): δ 6.34–6.28 (m, 1H), 5.25 (t, *J* = 4.0 Hz, 1H), 4.33–3.90 (m, 4H), 2.58–2.51 (m, 1H), 2.04–2.02 (m, 3H), 1.13–1.11 (m, 6H); HRMS-ESI (*m*/*z*): [M + Na]^+^ calcd for [C_10_H_16_O_6_Na]^+^, 255.0845; found, 255.0834.

#### 1-((2*S*,4*S*)-2-((Benzyloxy)methyl)-1,3-dioxolan-4-yl)-5-((*E*)-2-bromovinyl)pyrimidine-2,4(1*H*,3*H*)-dione (**22**)

To a stirred solution of (*E*)-5-bromovinyluracil (350 mg, 1.62 mmol) in dry DCM (12 mL) were added TBDMSOTf (0.96 mL, 4.21 mmol) and 2,4,6-collidine (0.55 mL, 4.21 mmol) at room temperature. The reaction mixture was stirred for 30 min. After that, a solution of **20** (375 mg, 1.78 mmol) in DCM (10 mL) was added dropwise followed by addition of TMSI (0.25 mL, 1.78 mmol). The mixture was stirred at room temperature for 3 h and quenched with a saturated aqueous solution of Na_2_S_2_O_3_. The organic layer was washed with brine, dried over Na_2_SO_4_, filtered, and concentrated under reduced pressure. The crude was purified by silica gel column chromatography (15–18% EtOAc/Hexane) as an eluent to give undesired α-isomer **21** (140 mg, 22%) as a white solid and desired β -isomer **22** (340 mg, 54%) as a white solid. ^1^H NMR (500 MHz, CDCl_3_): δ 8.68 (bs, 1H), 7.62 (s, 1H), 7.45 (d, *J* = 13.6 Hz, 1H), 6.71 (d, *J* = 13.6 Hz, 1H), 6.33 (d, *J* = 5.0 Hz, 1H), 5.18 (t, *J* = 2.9 Hz, 1H), 4.48 (dd, *J* = 3.1 and 12.6 Hz, 1H), 4.35–4.30 (m, 1H), 4.25 (d, *J* = 10.5 Hz, 1H), 4.18 (dd, *J* = 5.5 and 10.4 Hz, 1H), 2.59 (hep, *J* = 7.0 Hz, 1H), 1.20 (t, *J* = 7.5 Hz, 6H); ^13^C{^1^H} NMR (126 MHz, CDCl_3_): δ 176.6, 161.3, 149.6, 137.1, 128.1, 112.1, 110.8, 103.3, 81.5, 71.6, 61.9, 33.9, 19.1, 19.0; HRMS-ESI (*m*/*z*): [M + Na]^+^ calcd for [C_14_H_17_BrN_2_O_6_Na]^+^, 411.0168; found, 411.0163.

#### 5-((*E*)-2-Bromovinyl)-1-((2*S*,4*S*)-2-(hydroxymethyl)-1,3-dioxolan-4-yl)pyrimidine-2,4(1*H*,3*H*)-dione (**17**)

To a stirred solution of compound **22** (250 mg, 0.64 mmol) in MeOH (5 mL) in a sealed tube at 0 °C, a 7 N solution of NH_3_ in MeOH was added, and the mixture was stirred at rt for 16 h. The volatiles were removed under reduced pressure, and the obtained residue was triturated with diethyl ether (2 × 5 mL) to give l-BHDU, **17** (180 mg, 88%) as an off-white solid. [α]_D_^23^ = −6.1 (c 0.2.7, MeOH); ^1^H NMR (500 MHz, DMSO-*d*_6_): δ 8.15 (s, 1H), 7.21 (d, *J* = 13.5 Hz, 1H), 6.82 (d, *J* = 13.5 Hz, 1H), 6.21 (d, *J* = 5.5 Hz, 1H), 5.31 (t, *J* = 5.5 Hz, 1H), 4.97–4.94 (m, 1H), 4.31 (d, *J* = 10 Hz, 1H), 4.09 (dd, *J* = 10.0 and 5.5 Hz, 1H), 3.74–3.67 (m, 2H); ^13^C{^1^H} NMR (126 MHz, DMSO-*d*_6_): δ 162.2, 150.1, 140.1, 130.3, 110.2, 107.0, 105.8, 81.2, 71.1, 60.3; HRMS-ESI (*m*/*z*): [M + Na]^+^ calcd for [C_10_H_11_BrN_2_O_5_Na]^+^, 340.9749; found, 340.9741.

#### Bis(POM) Prodrug of l-BHDU-MP (POM-l-BHDU-MP, **24**)

To a stirred solution of l-BHDU (**17**, 120 mg, 0.37 mmol) and *N*-methylimidazole (0.24 mL, 2.9 mmol) in dry THF (5 mL) was added freshly prepared bis(POM) phosphorochloridate^26^**23** (750 mg, 2.17 mmol) dissolved in dry THF (3 mL) at 0 °C and stirred for 15 min. After that, the reaction mixture was warmed to rt and stirred for 3 h. The mixture was quenched with methanol, and solvents were removed under reduced pressure. The crude was purified by silica gel column chromatography (0.5% MeOH/DCM) to give **24** as a colorless sticky oil, which was crystallized in DCM/pentane to give a white solid. Yield: (130 mg, 55%). mp: 80–85 °C; ^1^H NMR (500 MHz, CDCl_3_): δ 8.47 (bs, 1H), 7.71 (s, 1H), 7.45 (d, *J* = 13.6 Hz, 1H), 6.79 (d, *J* = 13.6 Hz, 1H), 6.35 (d, *J* = 4.5 Hz, 1H), 5.72–5.65 (m, 4H), 5.15 (s, 1H), 4.43–4.39 (m, 1H), 4.35–4.31 (m, 1H), 4.26–4.21 (m, 1H), 4.20–4.17 (m, 1H), 1.23 (s, 18H); ^31^P NMR (202 MHz, CDCl_3_): δ −3.02; ^13^C{^1^H} NMR (126 MHz, CDCl_3_): δ 176.7, 161.4, 149.7, 137.4, 128.4, 112.2, 110.5, 102.9, 83.1, 81.1, 71.5, 65.2, 38.8, 26.9; HRMS-ESI (*m*/*z*): [M + Na]^+^ calcd for [C_22_H_32_BrN_2_O_12_NaP]^+^, 649.0774; found, *m*/*z* 649.0756.

## Data Availability

The data underlying this study are available in the published article and its online Supporting Information.

## Supporting Information Available

X-ray crystallographic data of compound **10** and copies of ^1^H, ^13^C, and ^31^P NMR spectra of all intermediates and final compounds **8**–**24** (PDF)
