# Supplementary material for: Enantioselective Synthesis of β-l-5-[(E)-2-Bromovinyl)-1-((2S,4S)-2-(hydroxymethyl)-1,3-(dioxolane-4-yl) Uracil)] (l-BHDU) via Chiral Pure l-Dioxolane
Source: J Org Chem. 2024 Jun 20;89(13):9313–21. doi: 10.1021/acs.joc.4c00399 (PMC11232004; doi:10.1021/acs.joc.4c00399)
Supplement: Supplementary file 1 — jo4c00399_si_001.pdf [file jo4c00399_si_001.pdf]

Supporting Information

Enantioselective Synthesis of *β*-*L*-5-[(*E*)-2-bromovinyl)-1-((2*S*,4*S*)-2-(hydroxymethyl)-1,3-(dioxolane-4-yl) uracil)] (L-BHDU) *via* Chiral Pure L-Dioxolane

Yugandhar Kothapalli, Chung K. Chu, and Uma S. Singh,*

Affiliations:

Department of Pharmaceutical and Biomedical Sciences, College of Pharmacy, University of Georgia, Athens, Georgia 30602, United States.

Corresponding author: Uma S. Singh, ussingh@uga.edu

Table of Contents

S. No.	Analytical Data	Page No.
1.	Analytical data for compounds **8 - 24**	S3 - S27
2.	X-Ray crystallographic information for compound **10**	S28 -S40
3.	Reference	S41

General Analytical Methods.

All reactions were conducted in round-bottomed flasks sealed with rubber septa under a nitrogen atmosphere unless otherwise stated. Reagents and anhydrous solvents were purchased and used without further purification. Reactions were monitored by thin-layer chromatography plates (TLC silica gel GF 250 microns) that were visualized using a UV lamp (254 nm) and developed with 15% sulfuric acid solution in methanol. Melting points were recorded on a digital melting point apparatus and were uncorrected. Nuclear magnetic spectra were recorded on 500 MHz for ^{1}H NMR and 125 MHz for ^{13}C NMR with tetramethylsilane as an internal standard. The chemical shifts (δ) are calculated with respect to the residual solvent peak and are given in ppm. Multiplicities are abbreviated as s (singlet), bs (broad singlet), d (doublet), t (triplet), q (quartet), m (multiplet), dd (double doublet) and dt (double triplet). Structural assignments were determined with additional information from gCOSY, gHSQC, and gHMBC experiments. Optical rotations were measured on a digital polarimeter. Optical rotations were measured on a JASCO DIP-370 digital polarimeter. High-resolution mass spectra were obtained on a ThermoFisher Scientific Orbitrap Q-Exactive instrument using electrospray ionization (ESI). Optical purity of chiral intermediates and final chiral compounds were determined by the chiral HPLC. Chiral HPLC/UV were determined with a Waters HPLC coupled to a photodiode array. 10 μL of samples 0.5 mg/mL in methanol, were injected, using an CHIRALCEL OX-H, 5 µm (4.6 X 250mm) column at 30 °C with flow rate 3.0 mL/min. The crystal structure was determined using an X-ray crystallography equipment called the Bruker D8 Quest PHOTON 100 CMOS X-ray diffractometer system with Incoatec Microfocus Source (IμS) monochromated Mo Kα radiation (λ=0.71073 Å, sealed tube) using phi and omega-scan technique. Deposition Number(s) CCDC number: 2312275 (for compound **10**, sample name **YK-02-69-P-3**), contain(s) the supplementary crystallographic data for this paper.

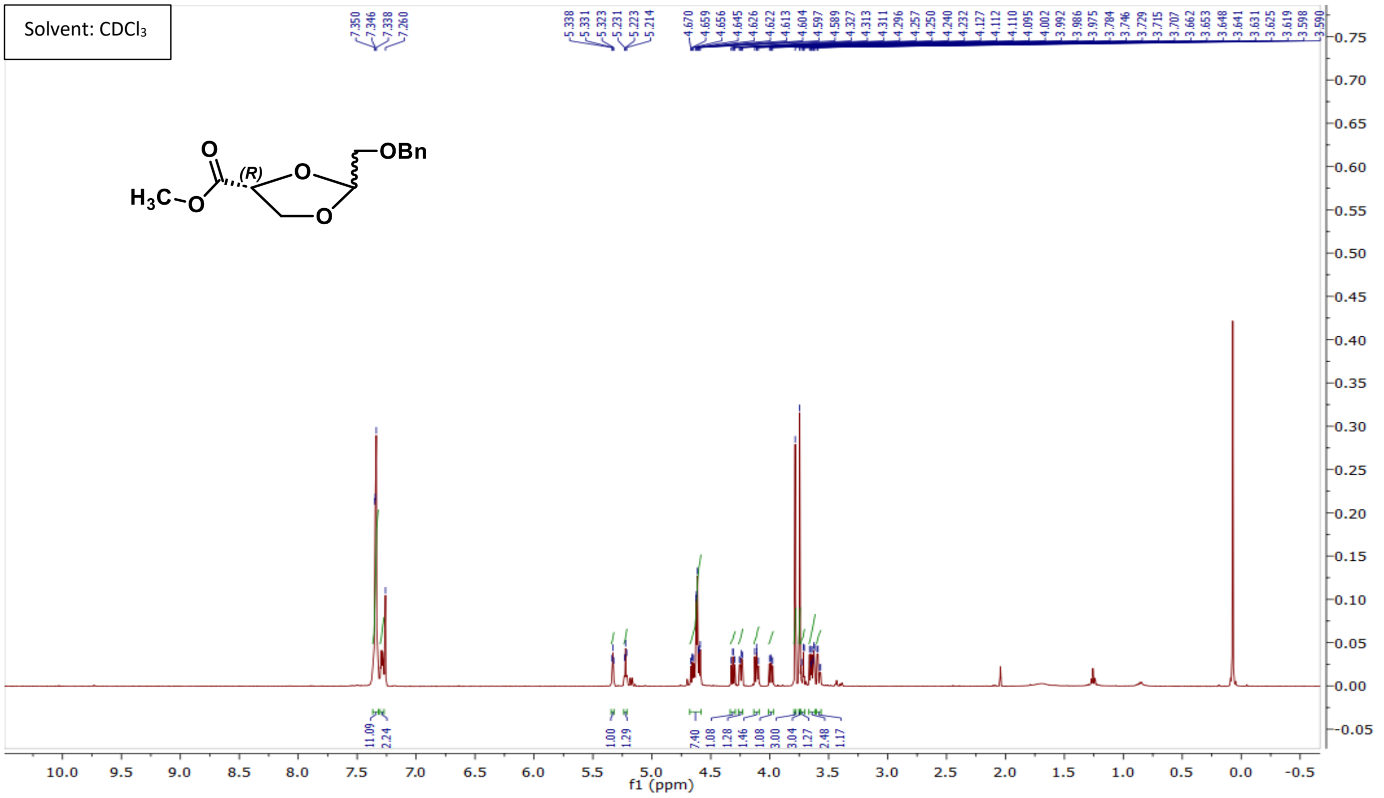

Figure S1. ^{1}H NMR (500 MHz) of compound **8**

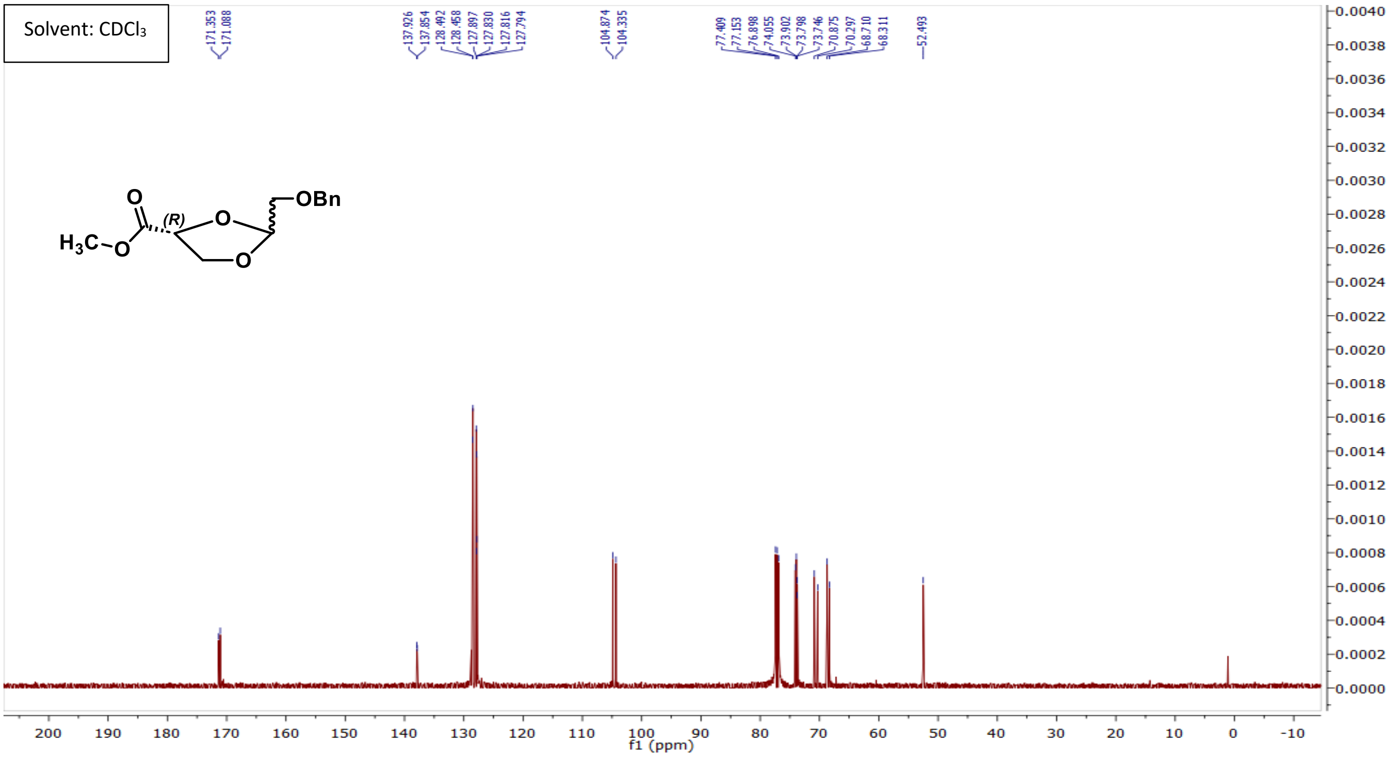

Figure S2. ^{13}C{^{1}H} (126 MHz) NMR spectrum of compound **8**

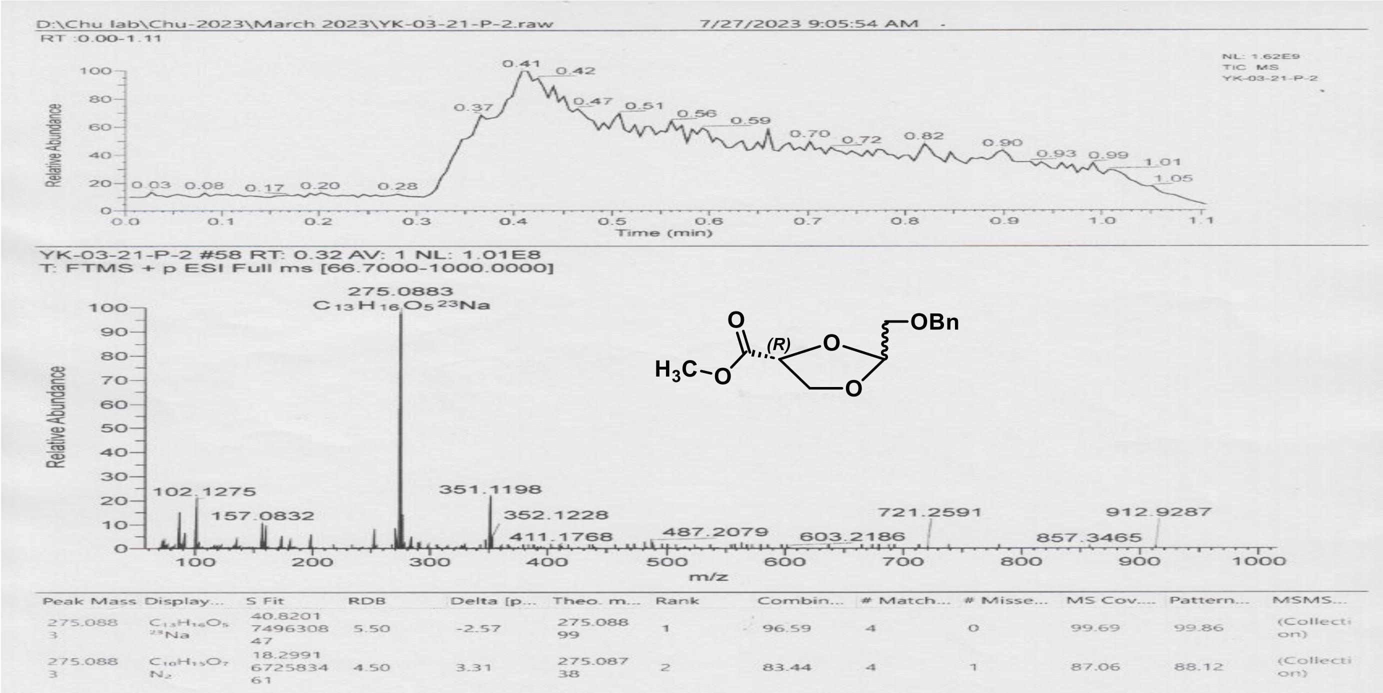

Figure S3. HRMS data of compound **8**

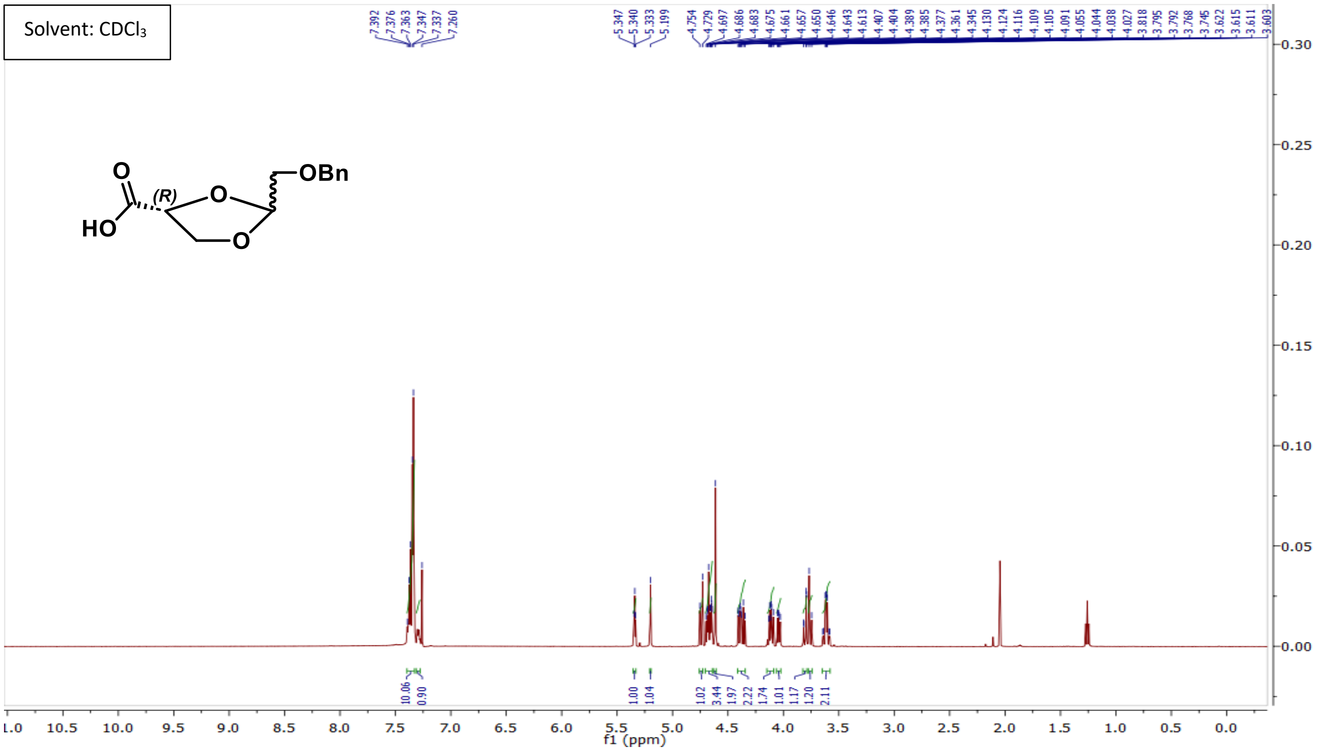

Figure S4. ^{1}H NMR (500 MHz) spectrum of compound **9**

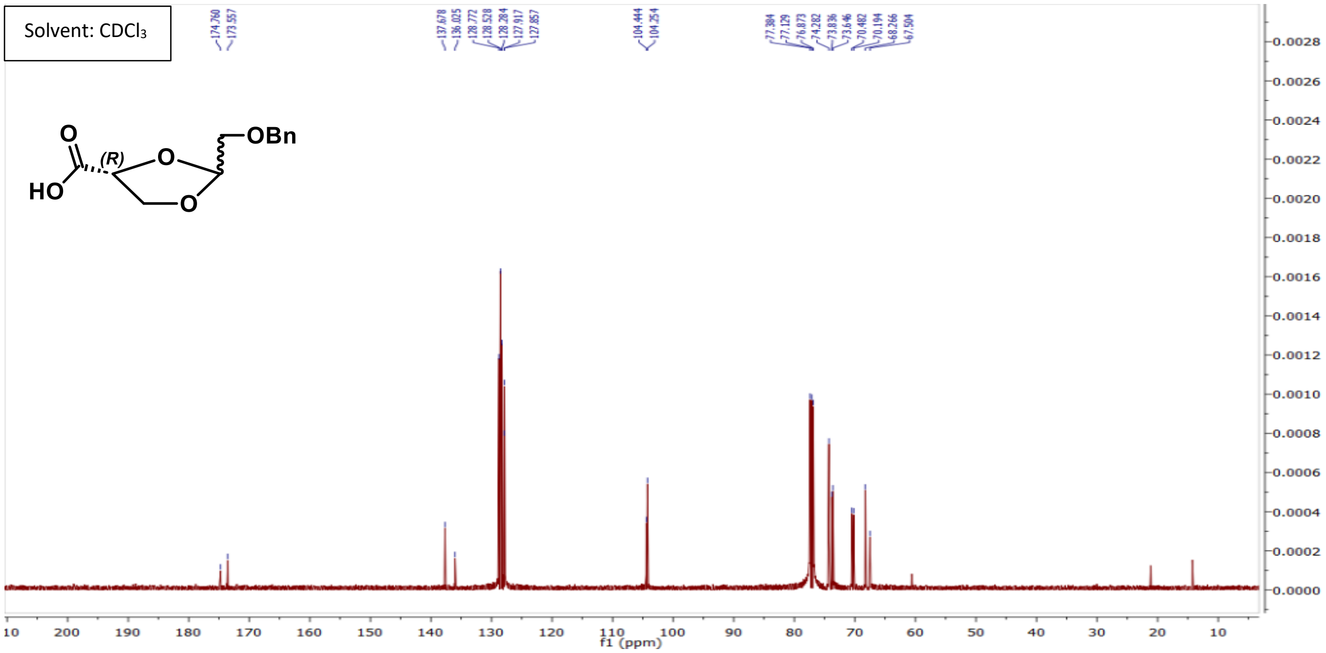

Figure S5. $^{13}C\{^{1}H\}$ (126MHz) NMR spectrum of compound **9**

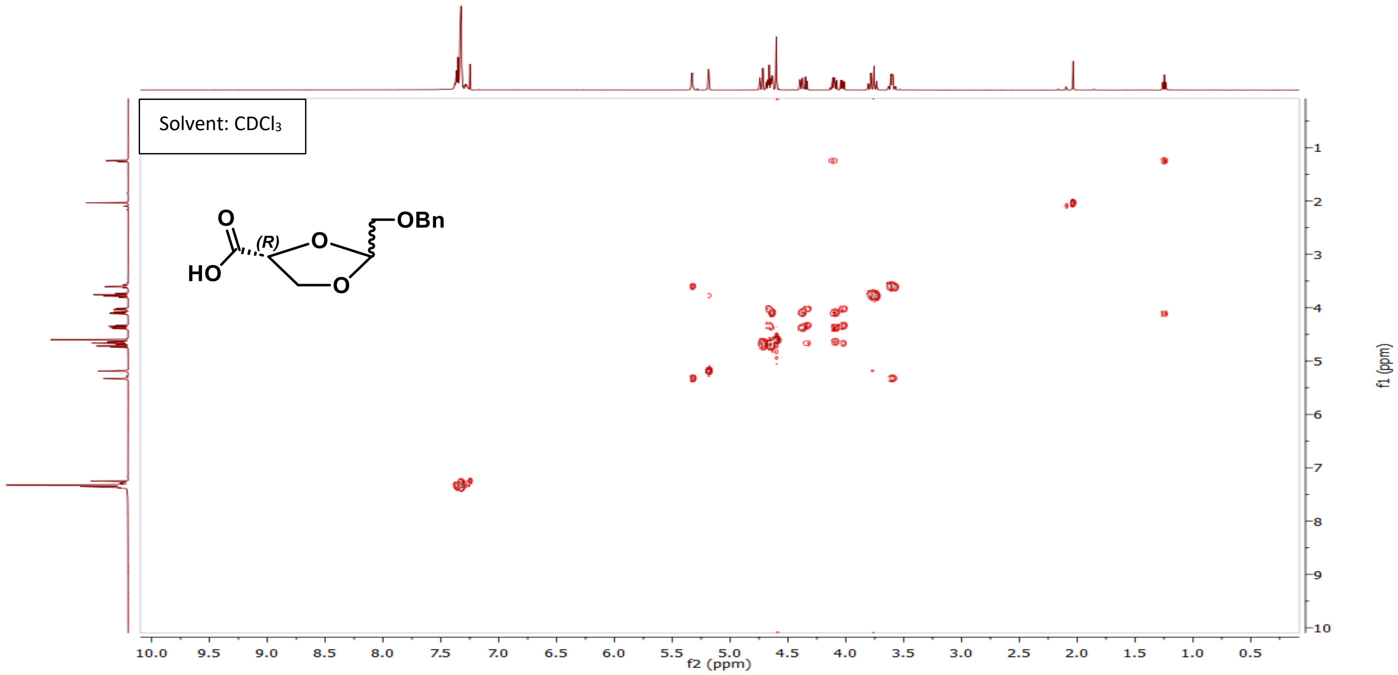

Figure S6. COSY NMR (500 MHz) spectrum of compound **9**

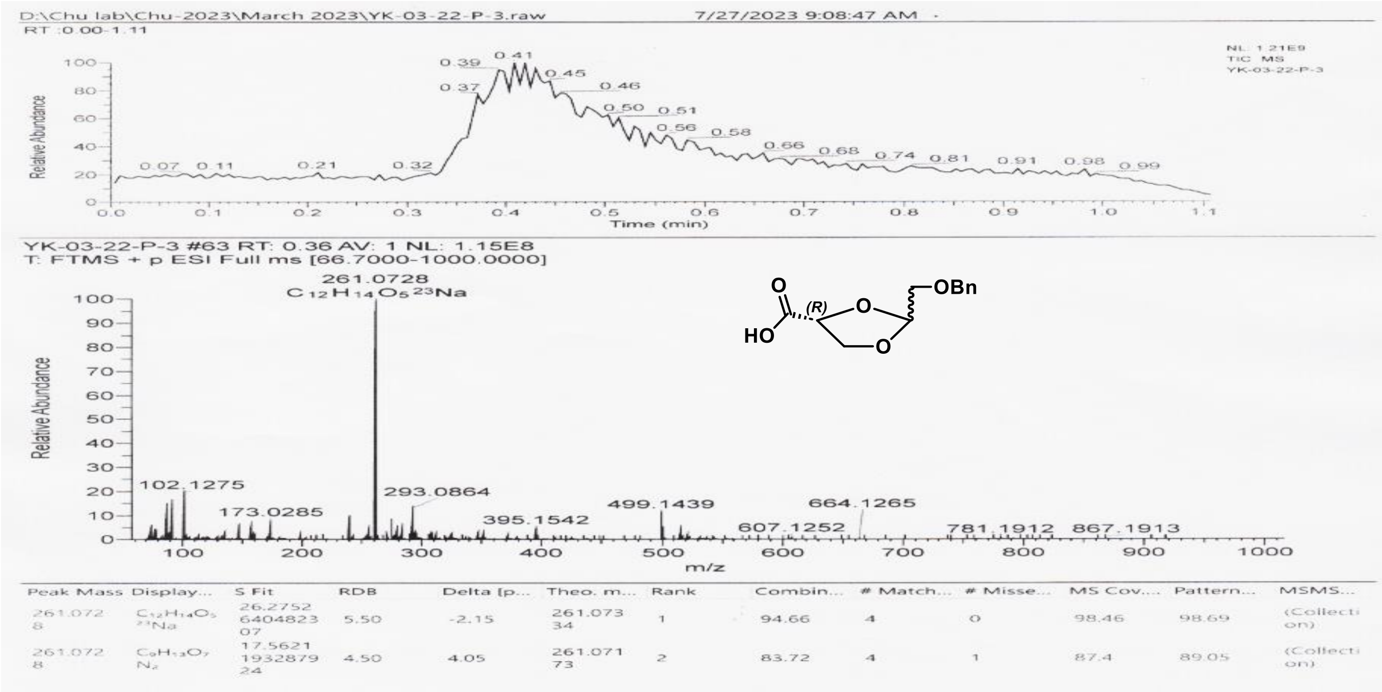

Figure S7. HRMS data of compound **9**

Chiral HPLC Method Conditions			
Instrument Method	3g-20_100bar	Sample	YK-02-18-P (RACEMIC)
Column	CHIRALCEL OX-H (4.6*250mm)5μ	Vial	1,1, B
Co-Solvent	0.5% DEA in Methanol	Injection Volume	10.00 μL
Col. Temp. (C)	30	Flow(ml/min)	3.00
		Co-Solvent% Values	20.0
		Back Pressure	99 Bar

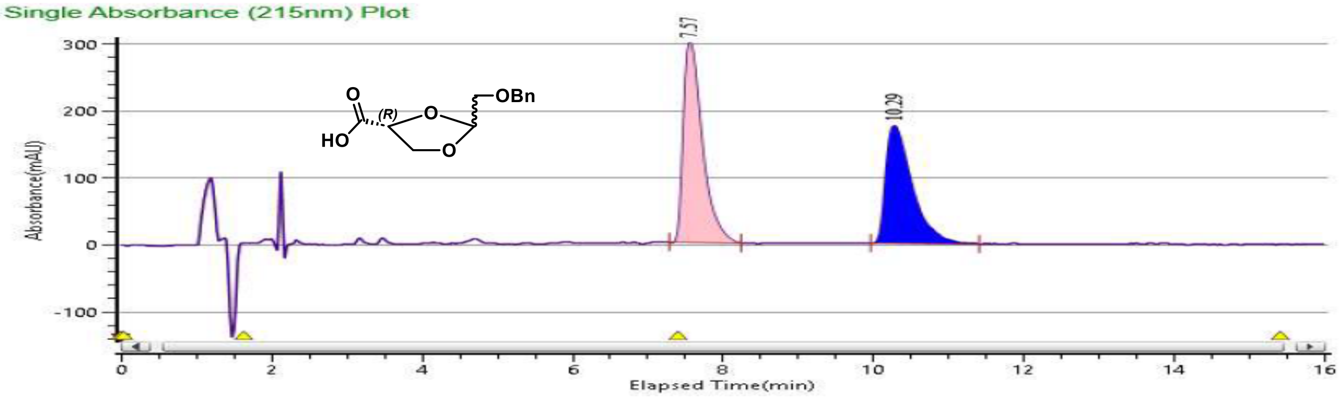

Peak Info[(W2998)SingleAbsorbance215]

Peak #	Ret. Time	Area %	Area
1	7.57 min	54.2366	4959.8412
2	10.29 min	45.7634	4184.9896

Figure S8. Chiral HPLC data of compound **9**

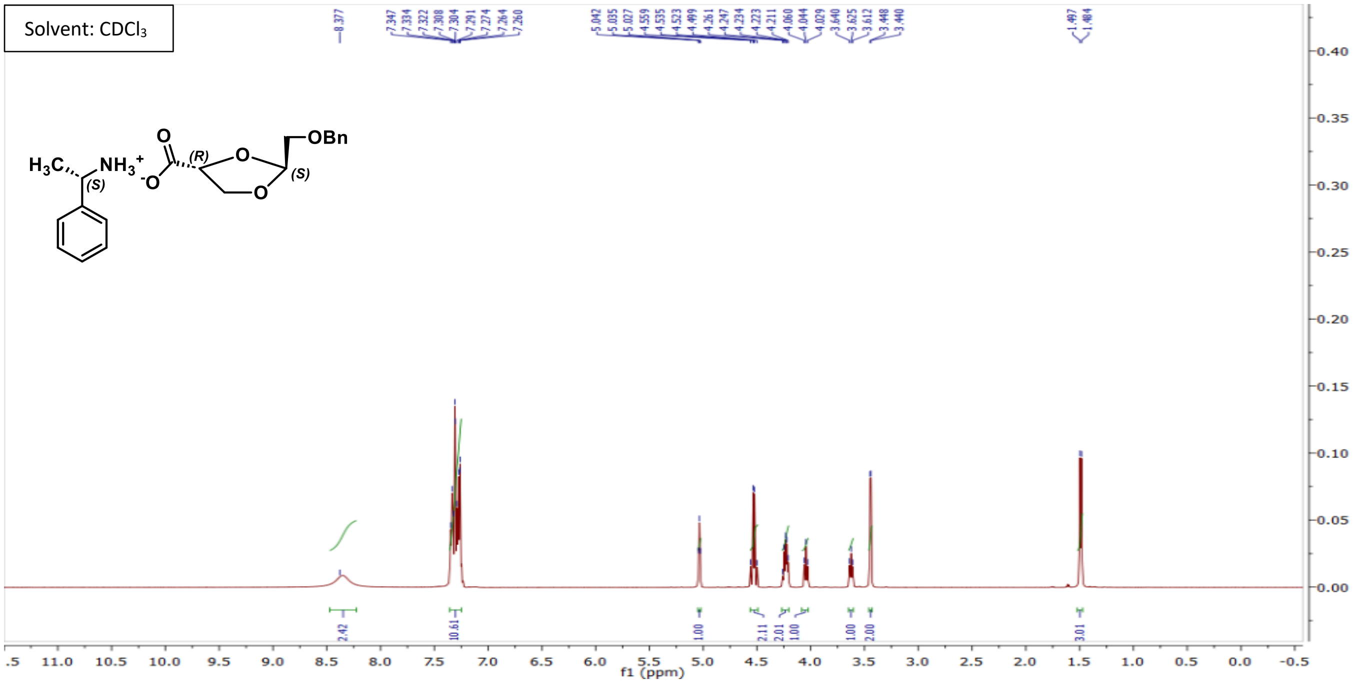

Figure S9. ^{1}H NMR (500 MHz) spectrum of compound **10**

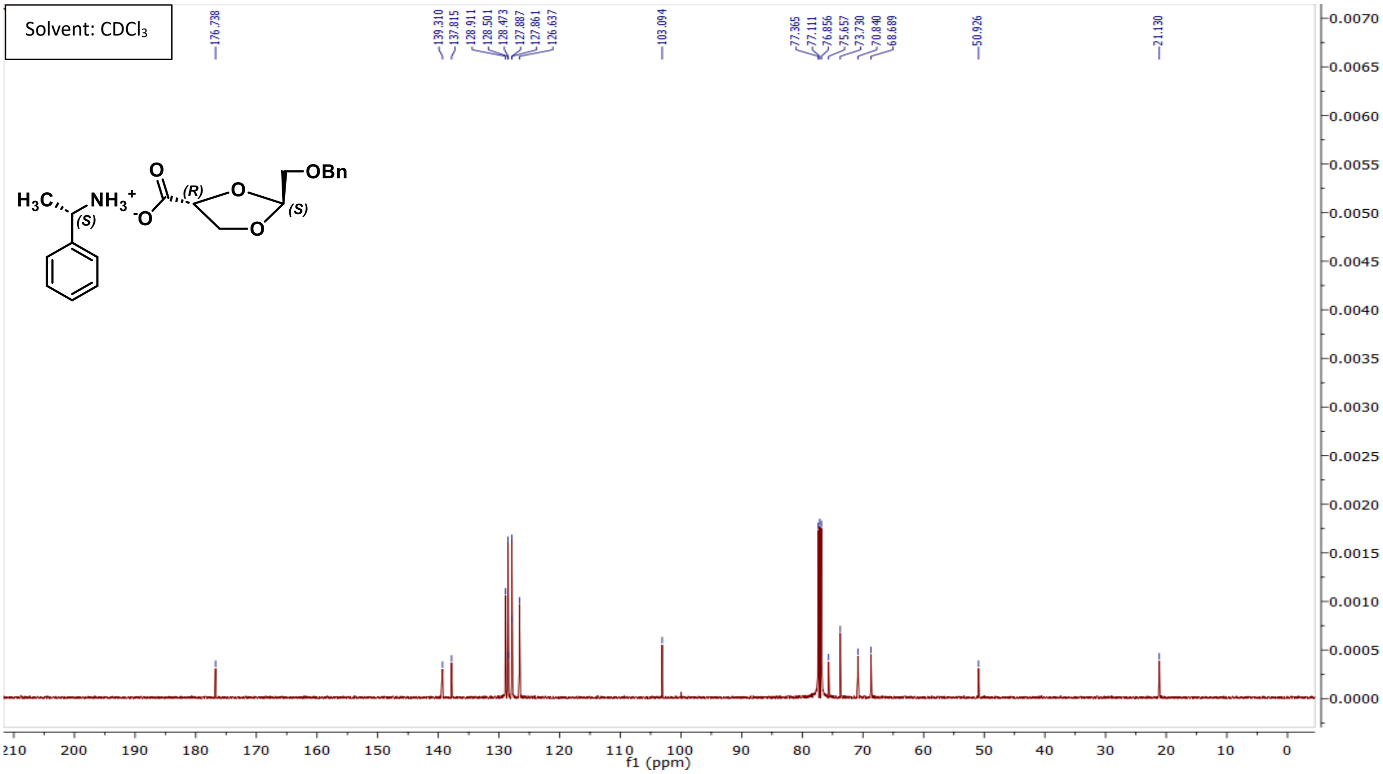

Figure S10. ^{13}C{^{1}H} (126 MHz) NMR spectrum of compound **10**

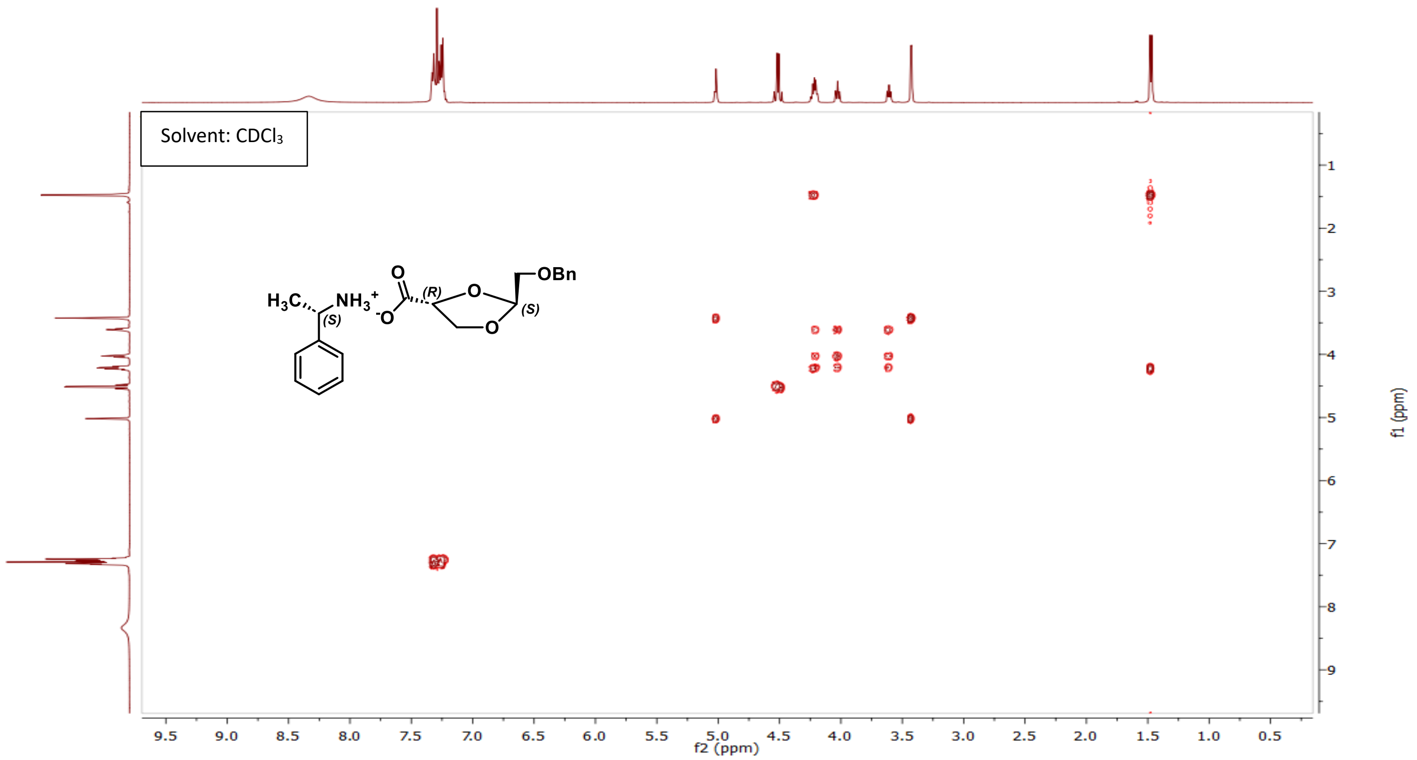

Figure S11. COSY NMR (500 MHz) spectrum of compound **10**

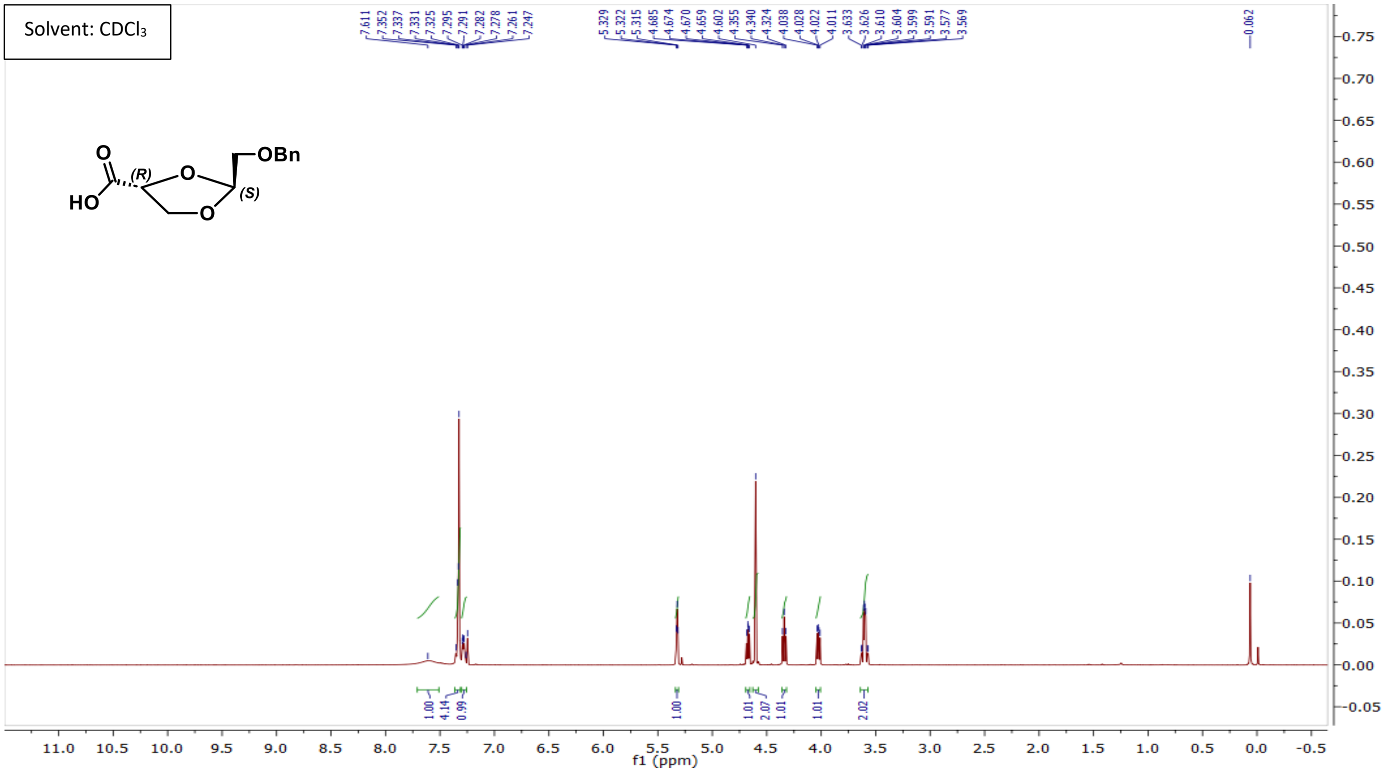

Figure S12. ^{1}H NMR (126 MHz) spectrum of compound **11**

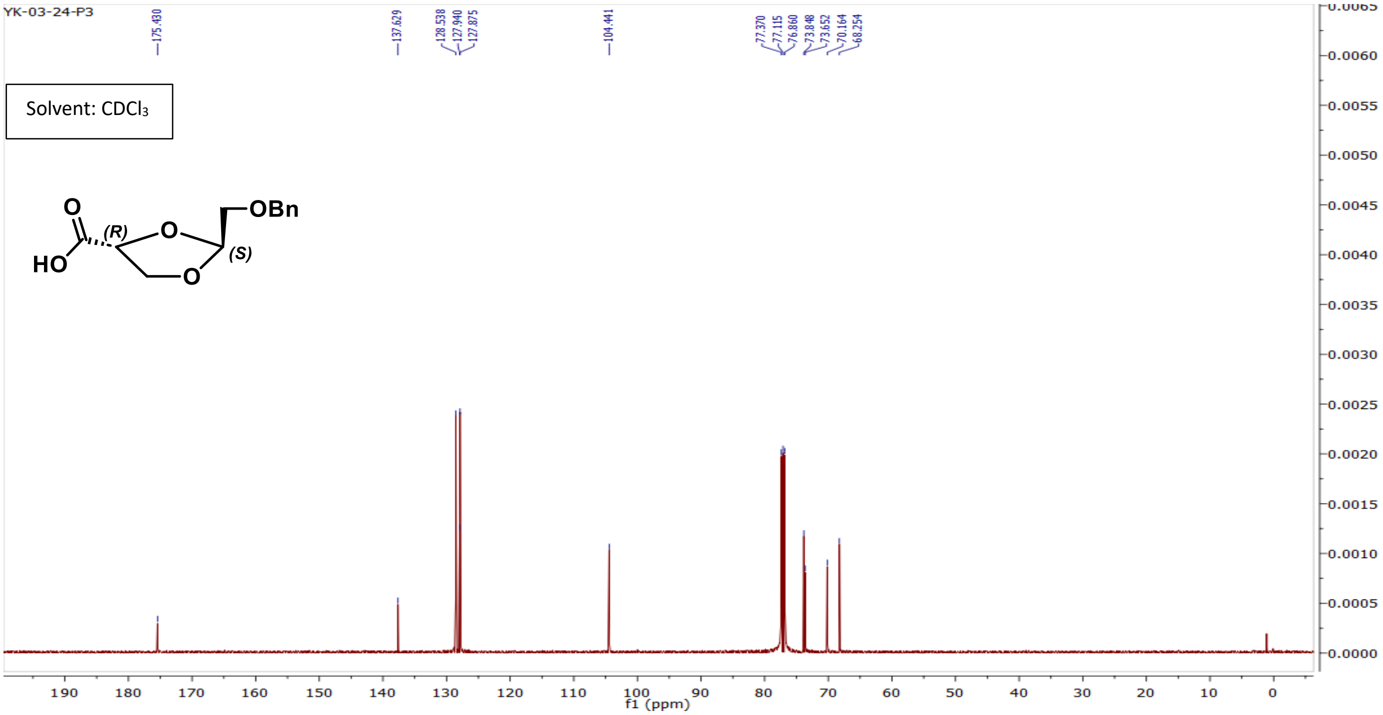

Figure S13. $^{13}C\{^{1}H\}$ (126 MHz) NMR spectrum of compound **11**

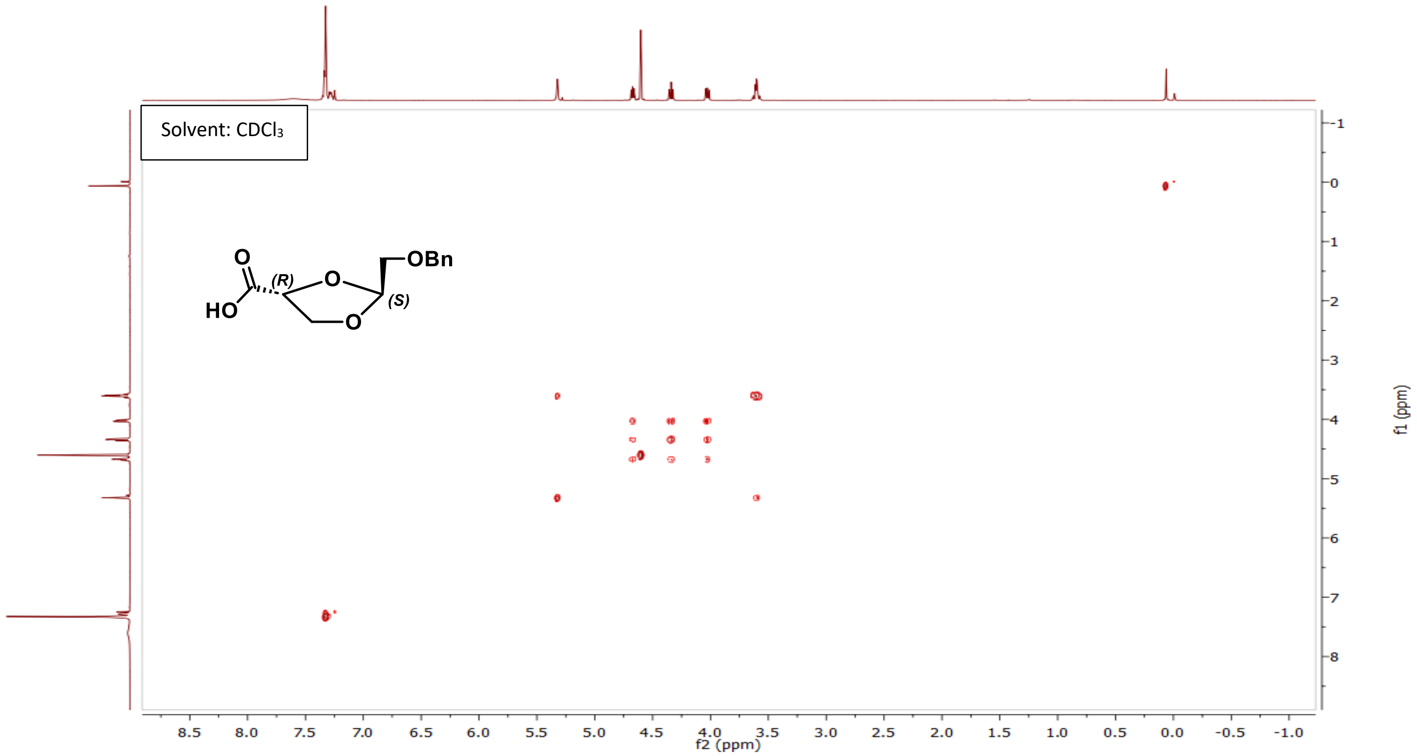

Figure S14. COSY NMR (500 MHz) spectrum of compound **11**

Chiral HPLC Method Conditions			
Instrument Method	3g-20_100bar	Sample	YK-02-24-P-3
Column	CHIRALCEL OX-H (4.6*250mm)5μ	Vial	2,1, A
Co-Solvent	0.5% DEA in Methanol	Injection Volume	10.00 μL
Col. Temp. (C)	30	Flow(ml/min)	3.00
		Co-Solvent% Values	20.0
		Back Pressure	101 Bar

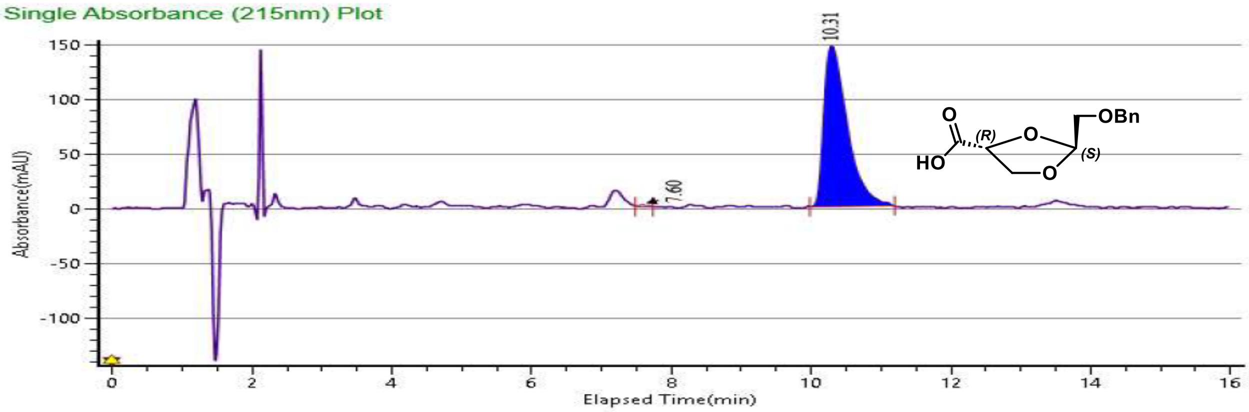

Peak Info[(W2998)SingleAbsorbance215]

Peak #	Ret. Time	Area %	Area
1	7.60 min	0.4084	13.5633
2	10.31 min	99.5916	3307.3364

Figure S15. Chiral HPLC data of compound **11** (L-dioxolane)

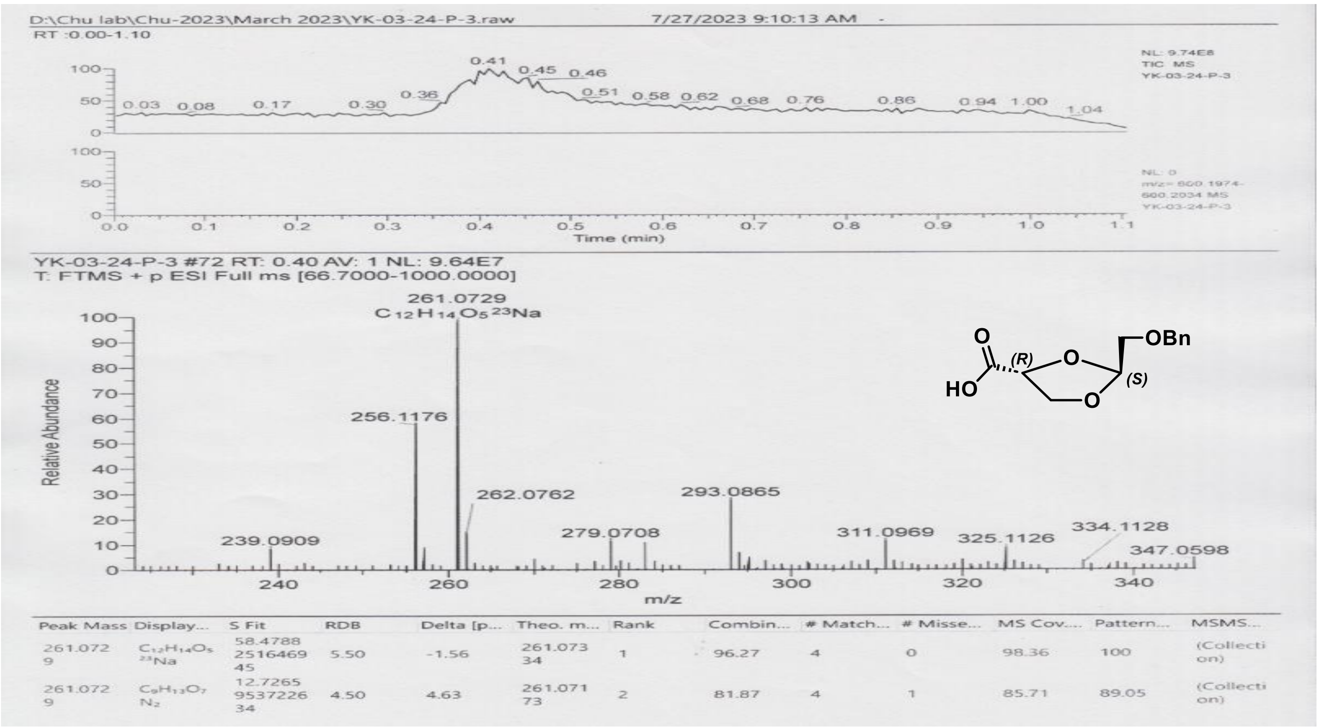

Figure S16. HRMS data of compound **11**

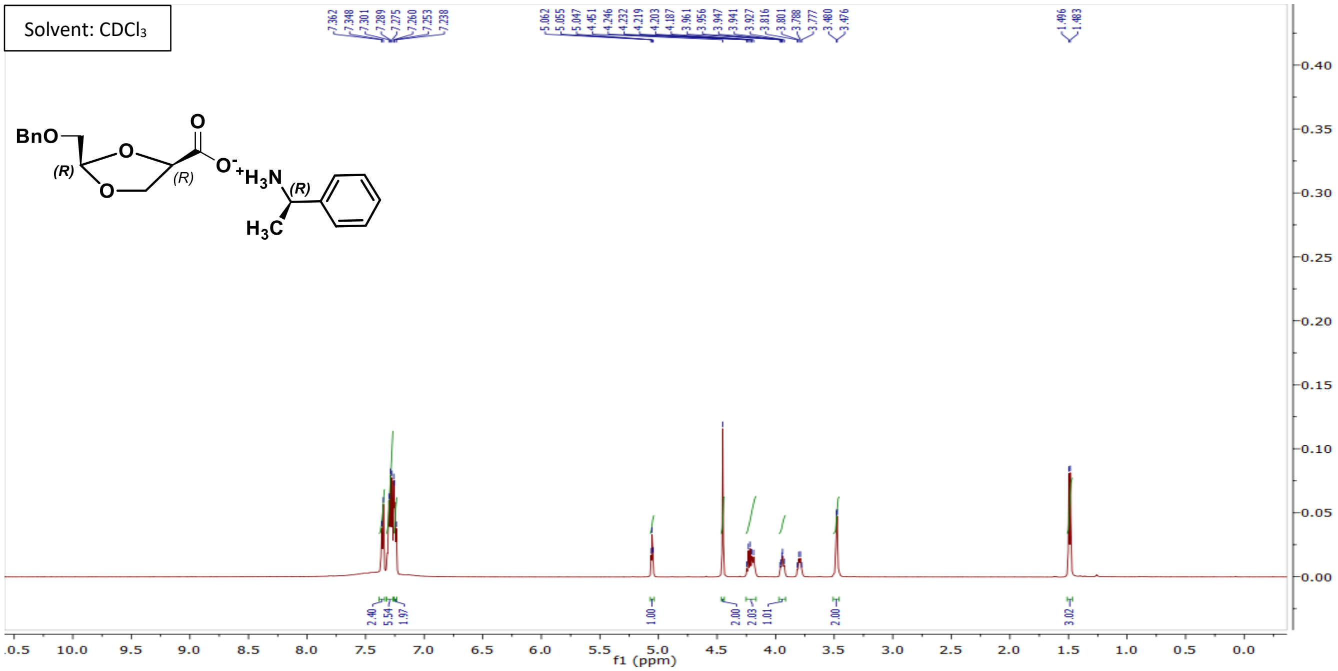

Figure S17. ^{1}H NMR (500 MHz) spectrum of compound **12**

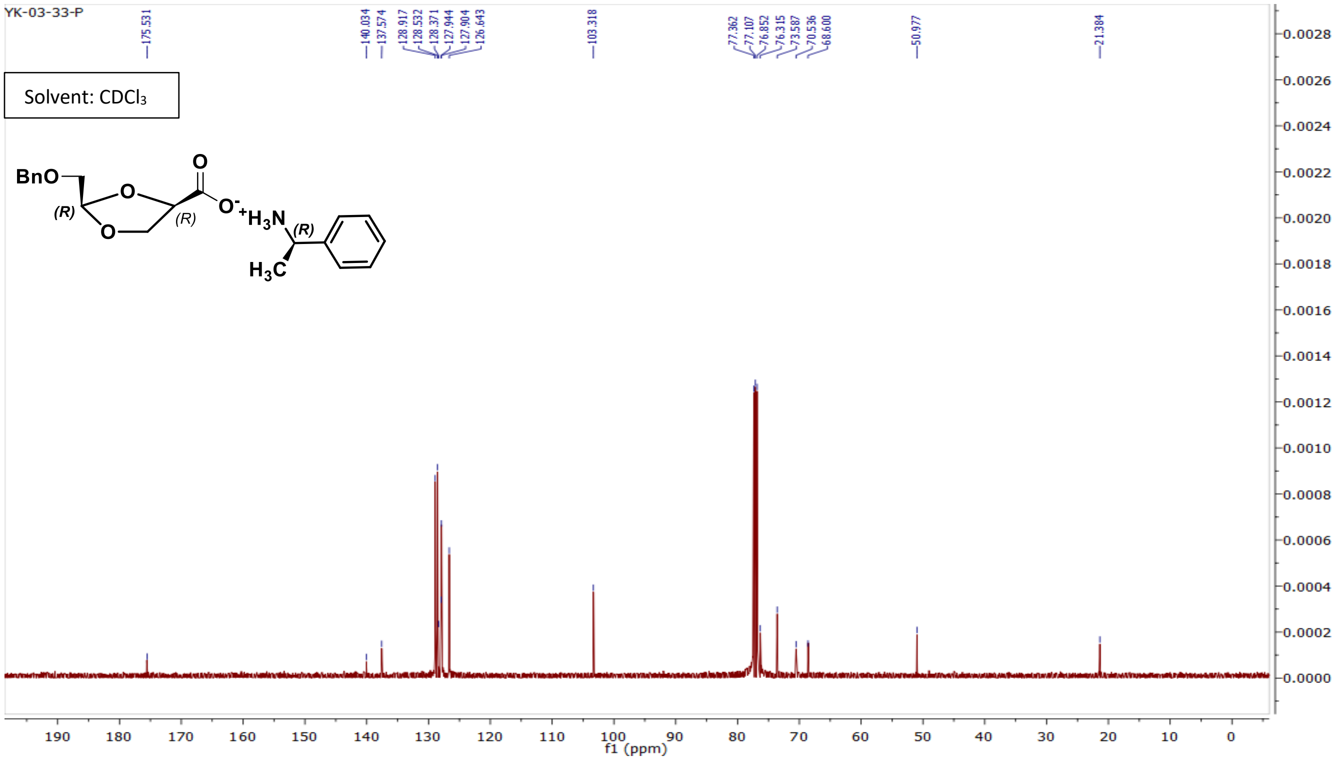

Figure S18. ^{13}C{^{1}H} (126 MHz) NMR spectrum of compound **12**

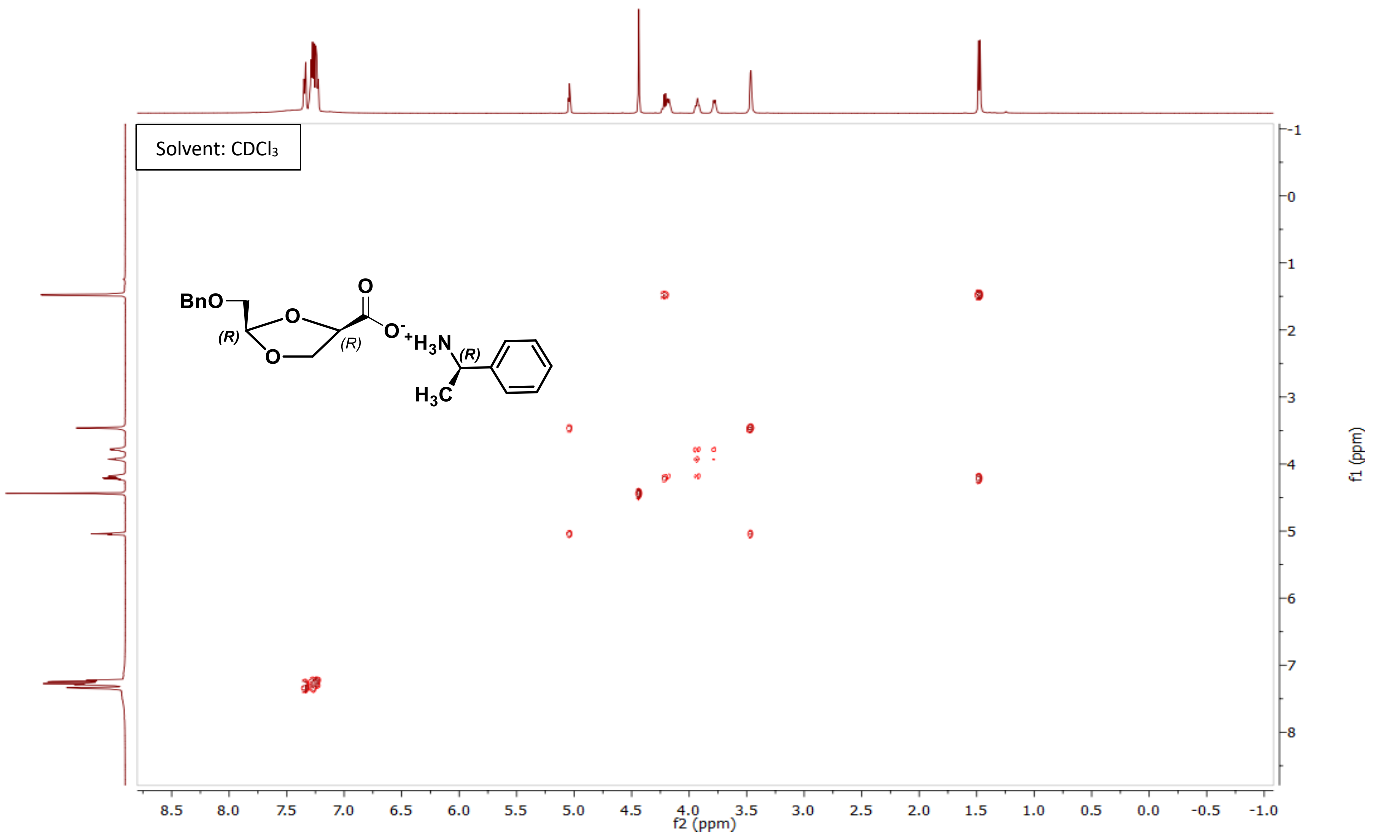

Figure S19. COSY NMR (500 MHz) spectrum of compound **12**

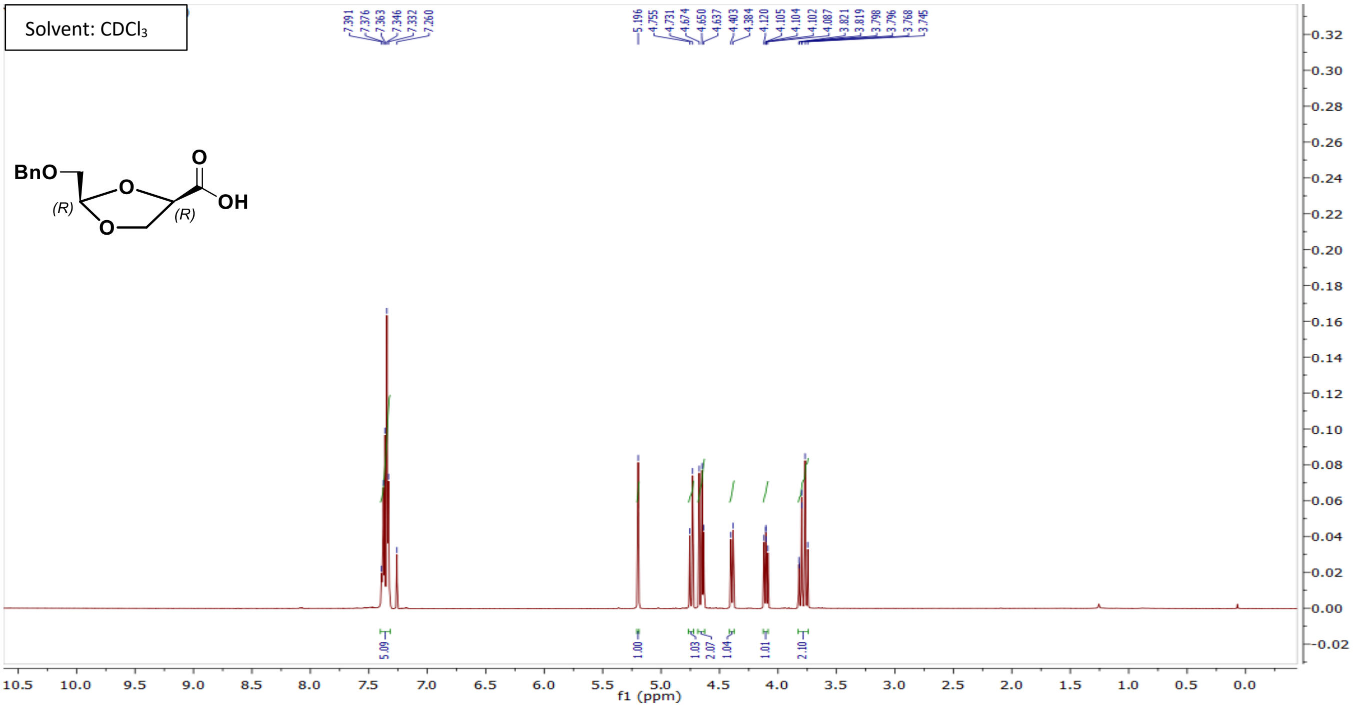

Figure S20. ^{1}H NMR (500 MHz) spectrum of compound **13**

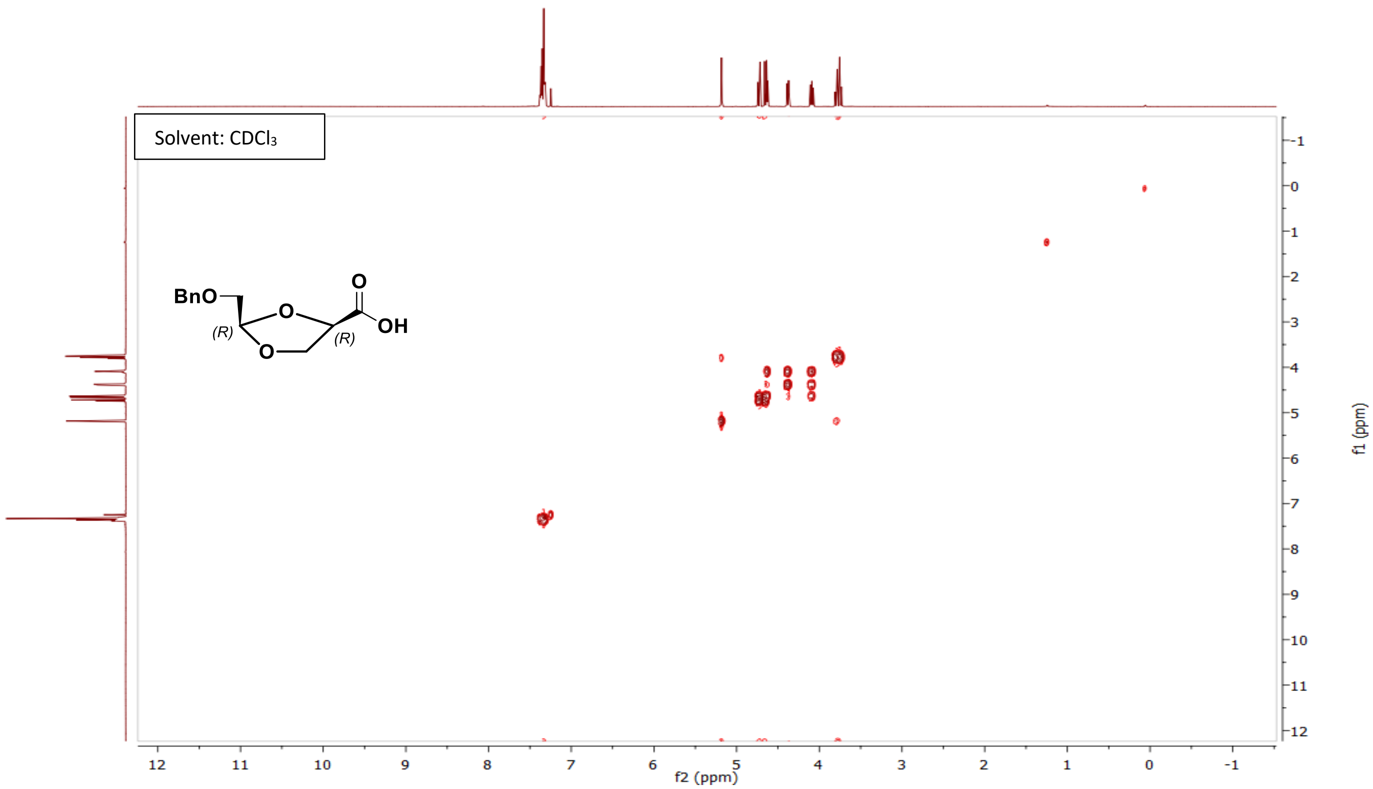

Figure S21. COSY NMR (500 MHz) spectrum of compound **13**

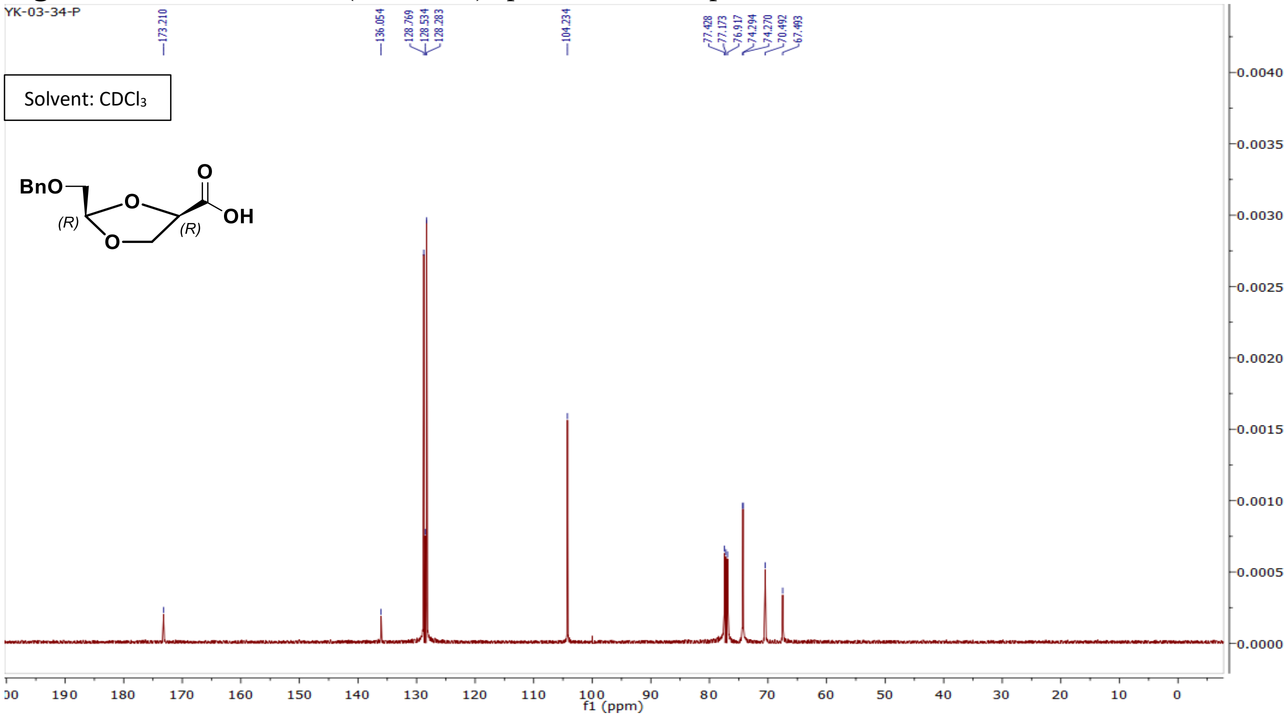

Figure S22. $^{13}C\{^{1}H\}$ (126 MHz) NMR spectrum of compound **13**

Chiral HPLC Method Conditions			
Instrument Method	3g-20_100bar	Sample	YK-02-18-P (NON-POLAR SPOT)
Column	CHIRALCEL OX-H (4.6*250mm)5µ	Vial	1,2,B
Co-Solvent	0.5% DEA in Methanol	Injection Volume	10.00 µL
Col. Temp. (C)	30	Flow(ml/min)	3.00
		Co-Solvent% Values	20.0
		Back Pressure	99 Bar

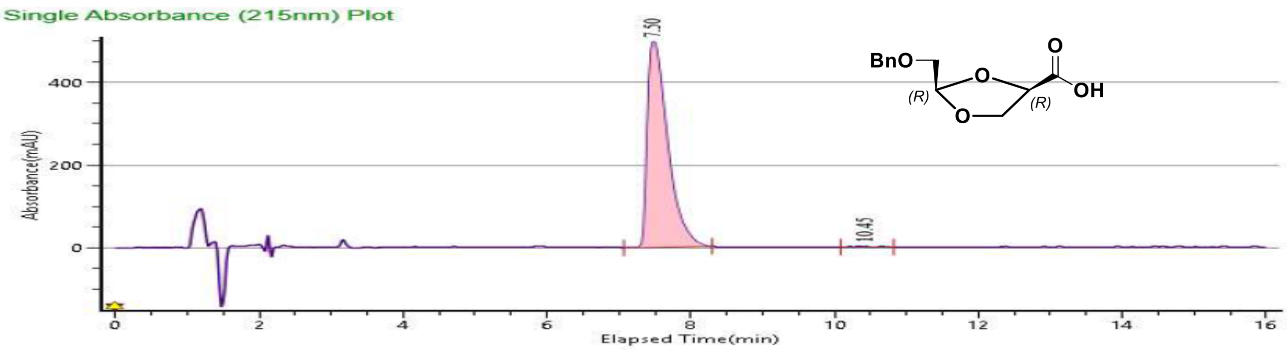

Peak Info[(W2998)SingleAbsorbance215]

Peak #	Ret. Time	Area %	Area
1	7.50 min	99.6729	9339.8586
2	10.45 min	0.3271	30.6472

Figure S23. Chiral HPLC data of compound **13** (D-dioxolane)

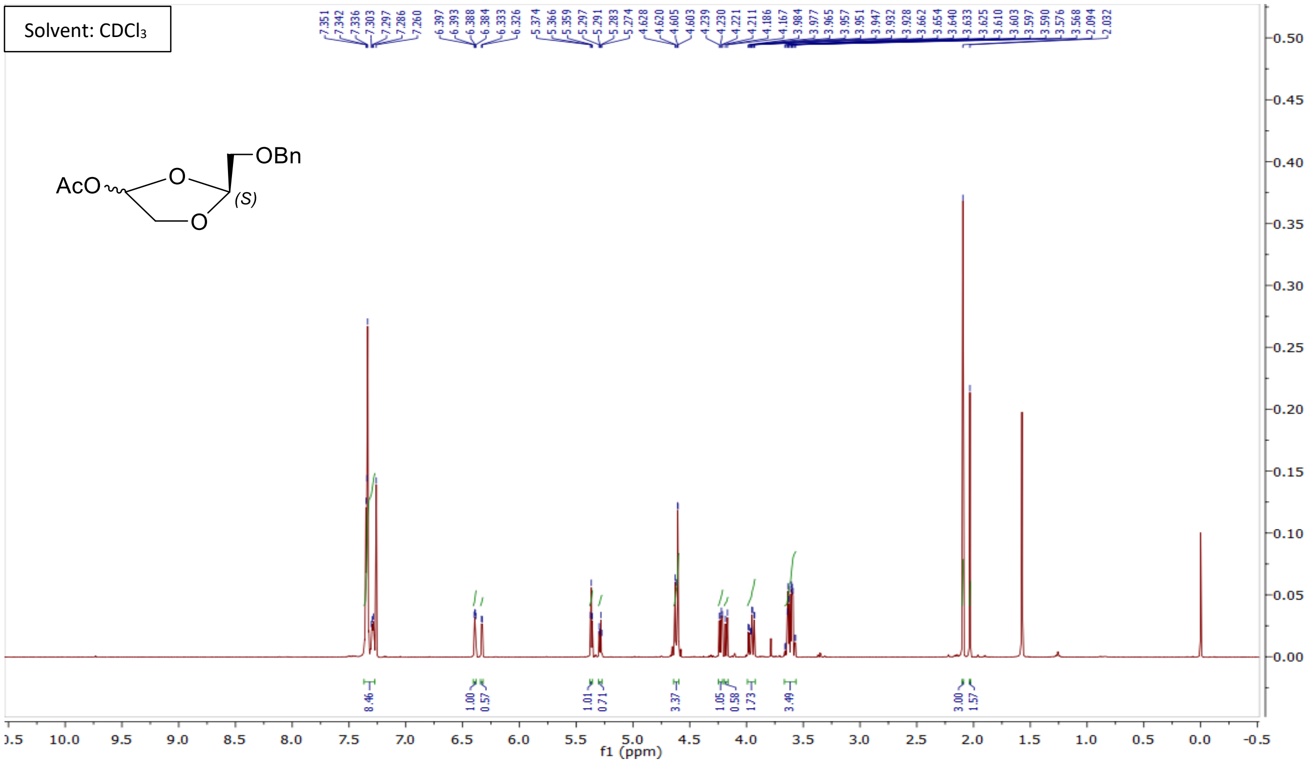

Figure S24. ^{1}H NMR (500 MHz) spectrum of compound **14**

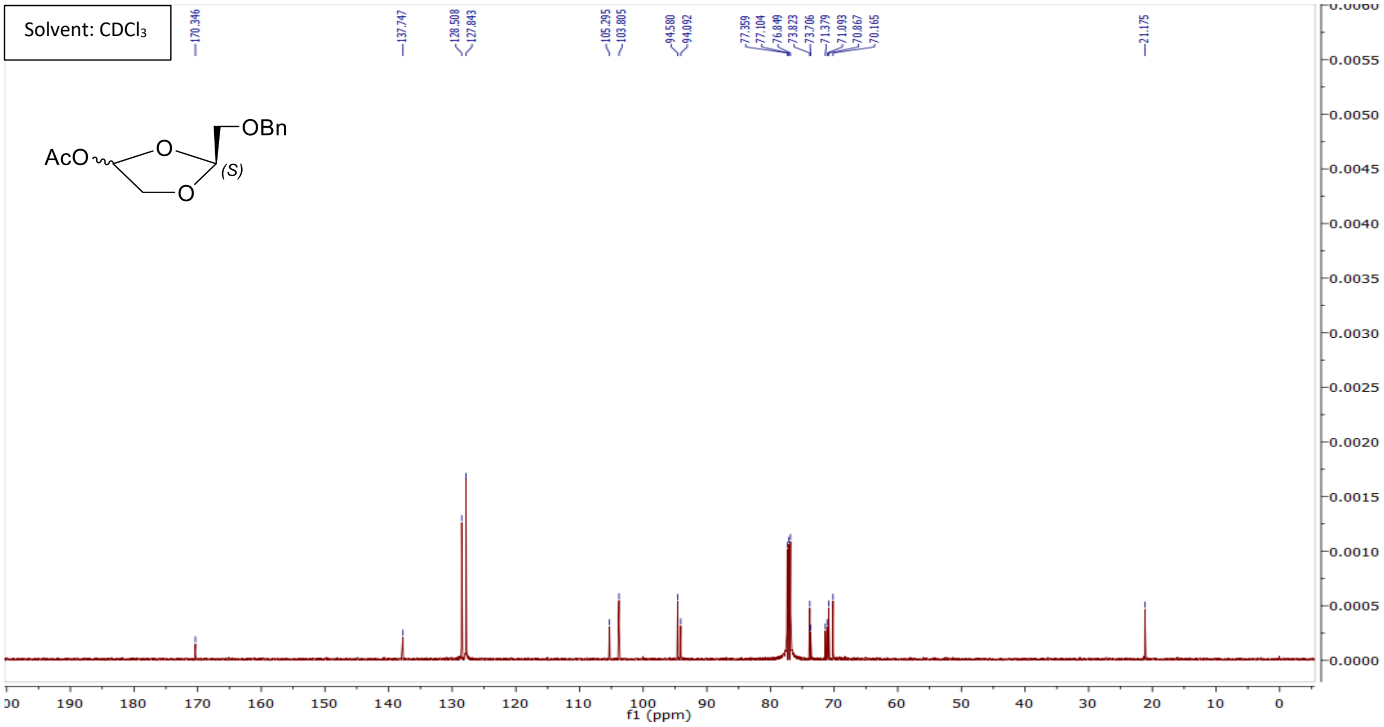

Figure S25. $^{13}C\{^{1}H\}$ (126 MHz) NMR spectrum of compound **14**

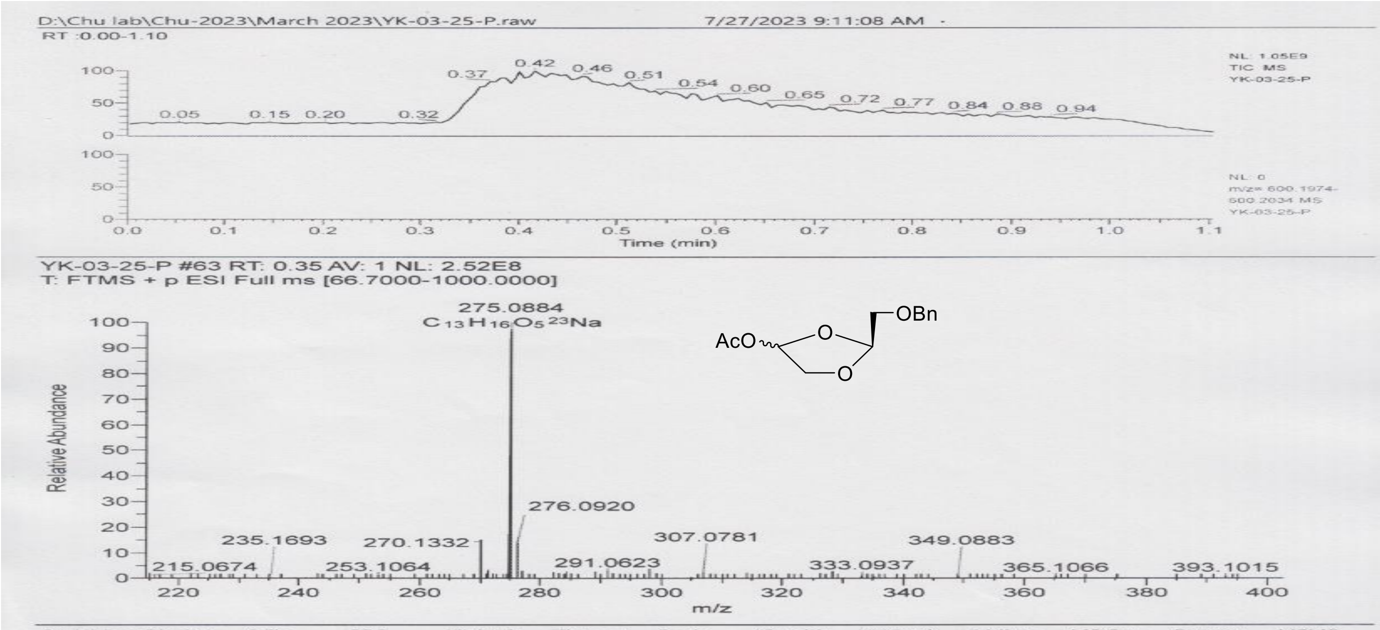

Peak Mass	Display...	S Fit	RDB	Delta [p...	Theo. m...	Rank	Combin...	# Match...	# Misse...	MS Cov....	Pattern...	MSMS...
275.088 4	$C_{13}H_{16}O_5$ ^{23}Na	52.3890 3490600 67	5.50	-2.24	275.088 99	1	97.32	4	0	99.82	100	(Collecti on)
275.088 4	$C_{10}H_{15}O_7$ N_2	18.1042 2652931 8	4.50	3.64	275.087 38	2	83.34	4	1	86.96	88.12	(Collecti on)

Figure S26. HRMS data of compound **14**

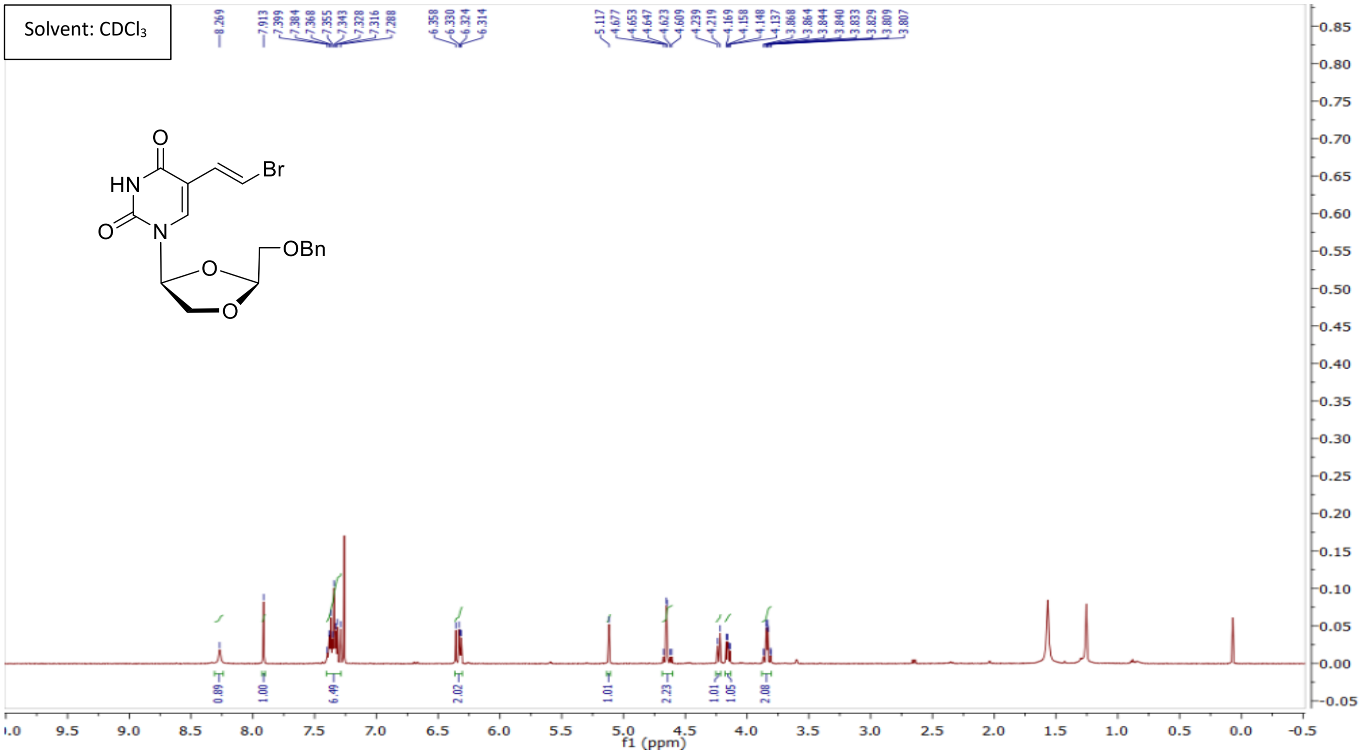

Figure S27. ^{1}H NMR (500 MHz) spectrum of compound **15**

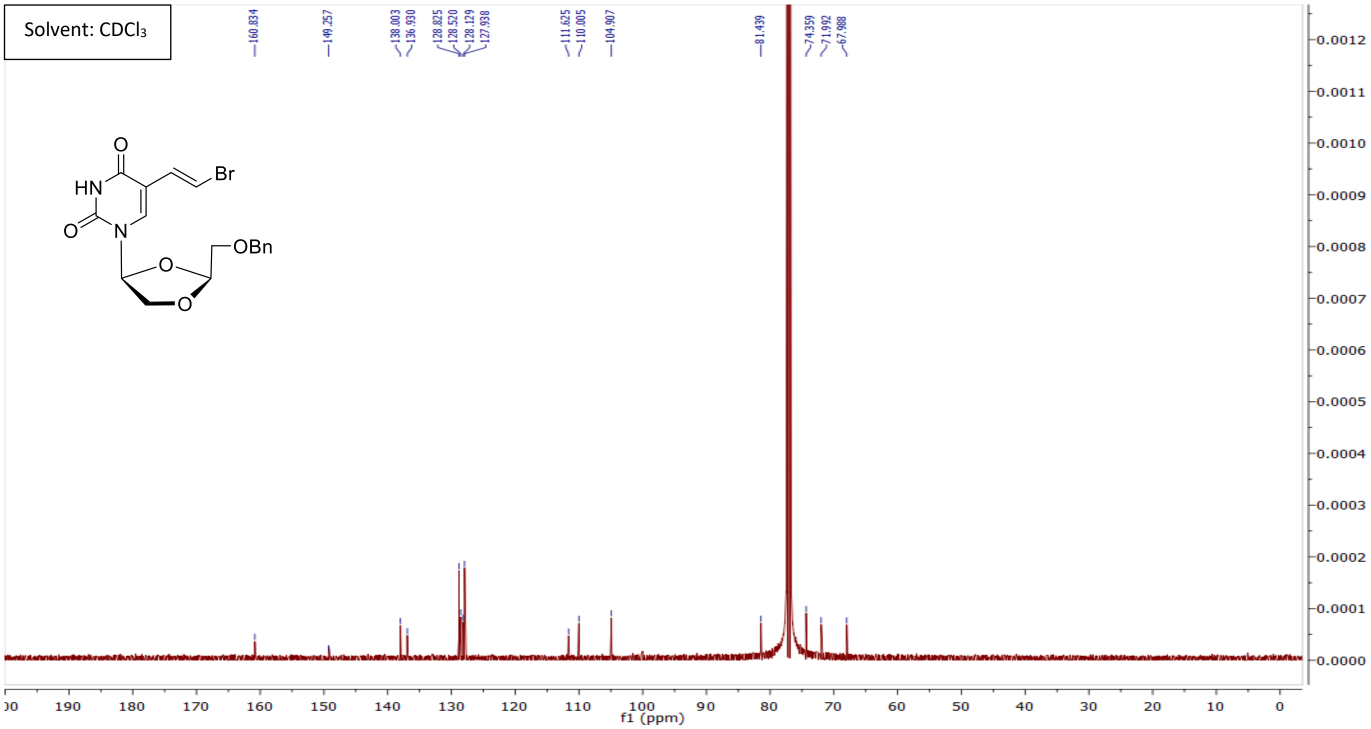

Figure S28. $^{13}C\{^{1}H\}$ (126 MHz) NMR spectrum of compound **15**

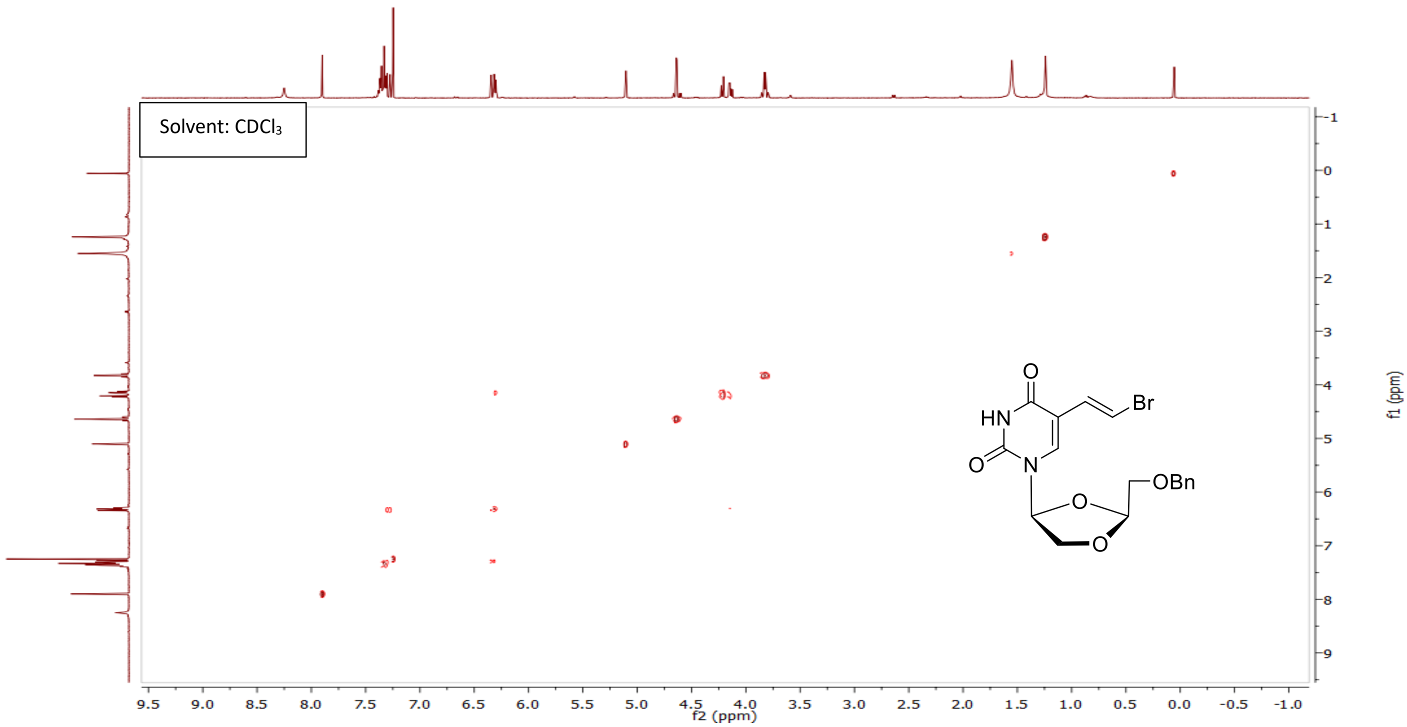

Figure S29. COSY NMR (500 MHz) spectrum of compound **15**

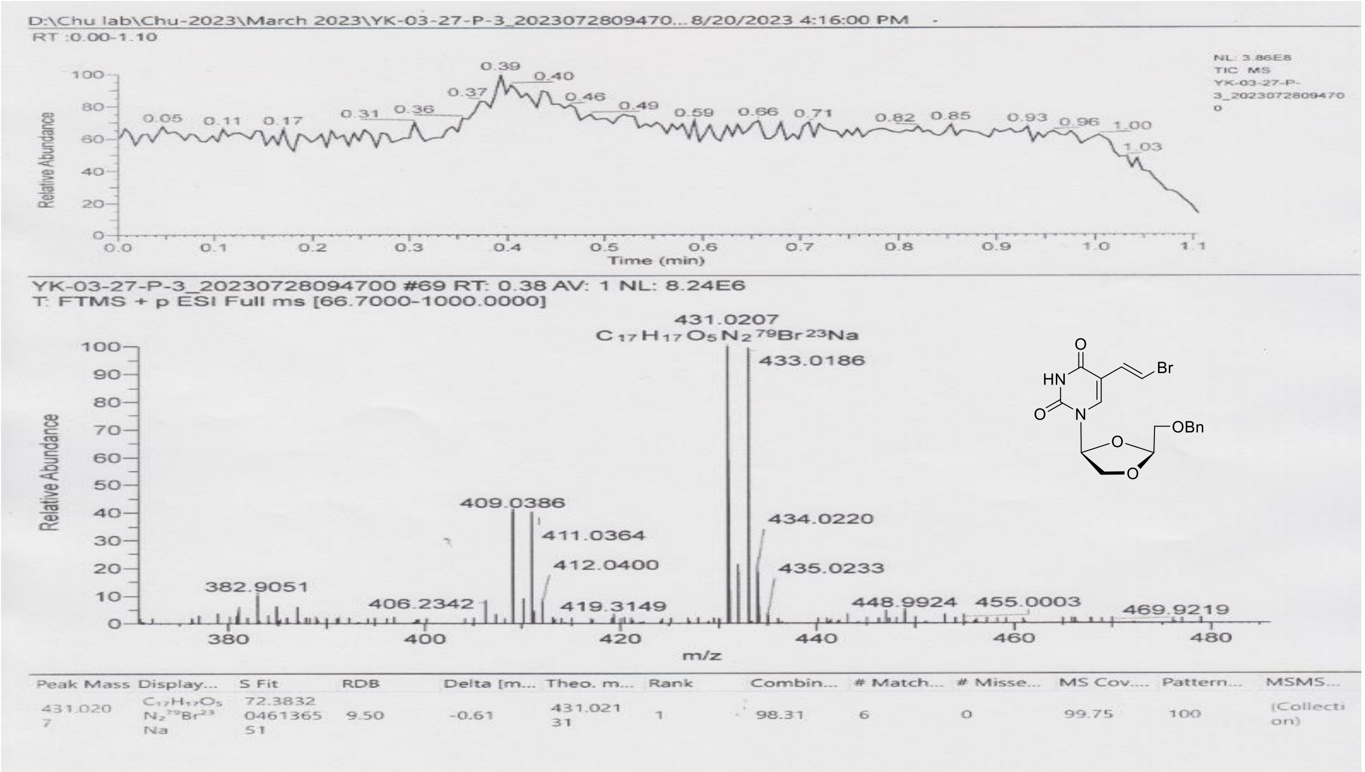

Figure S30. HRMS data of compound **15**

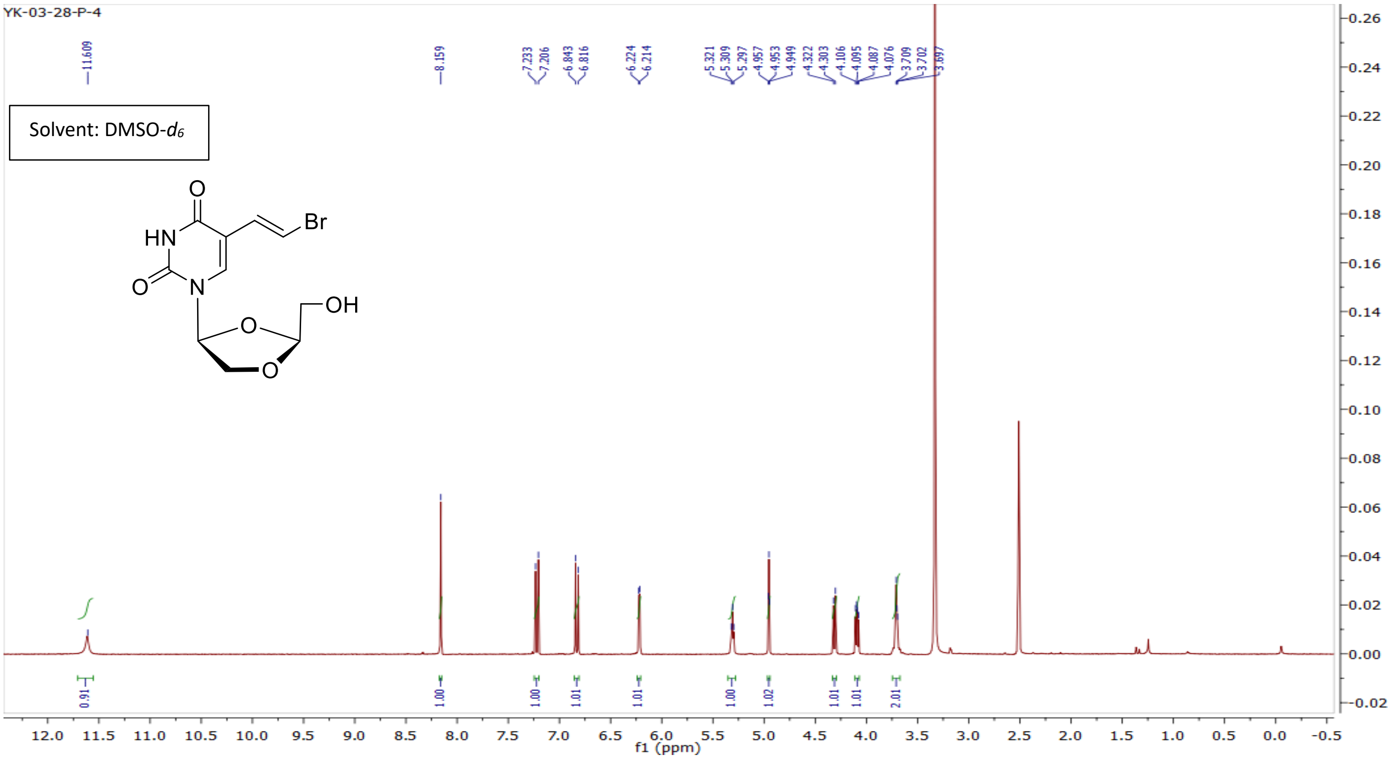

Figure S31. ^{1}H NMR (500 MHz) spectrum of L-BHDU **17**

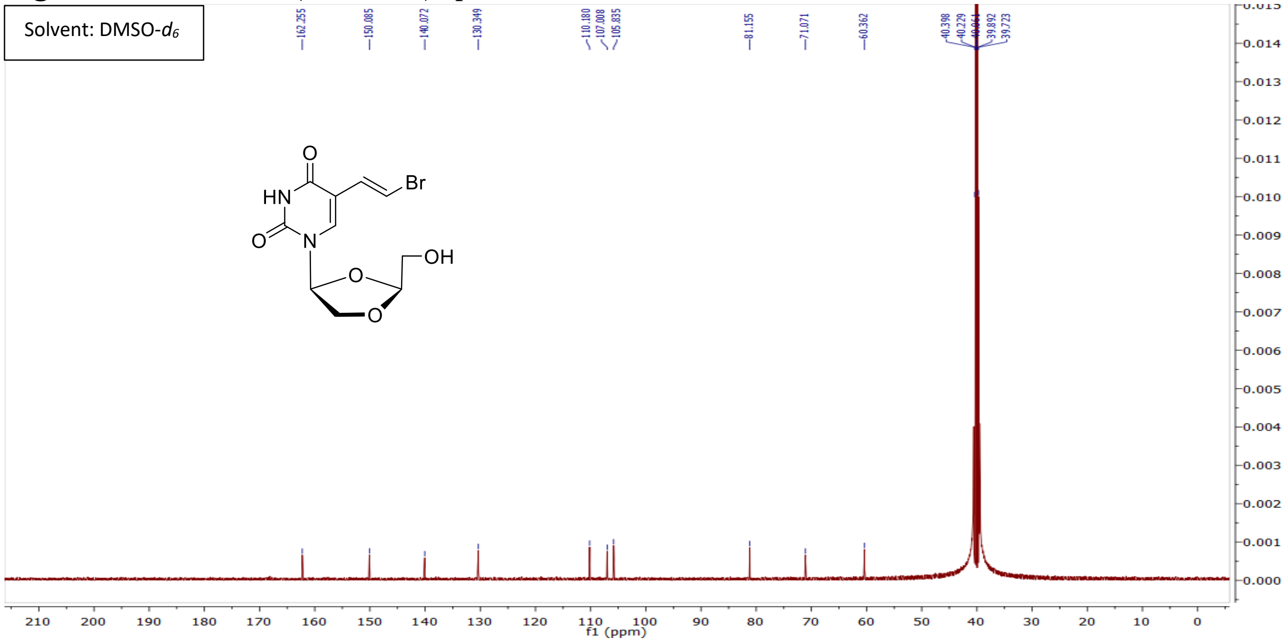

Figure S32. $^{13}C\{^{1}H\}$ (126 MHz) NMR spectrum of L-BHDU **17**

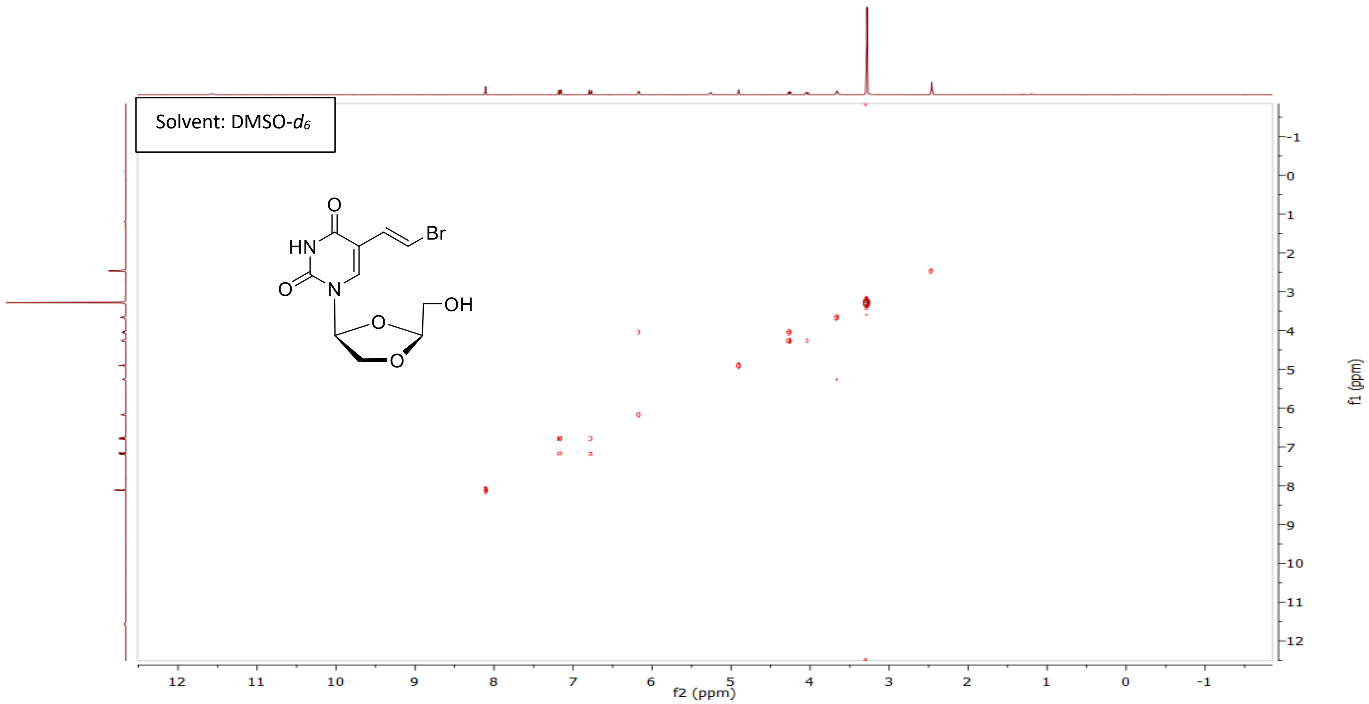

Figure S33. COSY NMR (500 MHz) spectrum of L-BHDU **17**

Chiral HPLC Method Conditions			
Column	CHIRALPAK AS-3 (4.6*150MM)3µM	Sample	LBHDU- Authentic Sample
Co-solvent	0.5% DEA in METHANOL	Vial	1:E,2
Total Flow	3 mL/min	Injection Volume	10.00 µL
% of CO2	85	Run Time	20 minutes
% of Co-Solvent	15	Date Acquired	22-Jun-2023
ABPR	1500psi	Acq. Method Set:	CM2_C1_SolveB1_3g_15
Column Temperature	30 ℃		

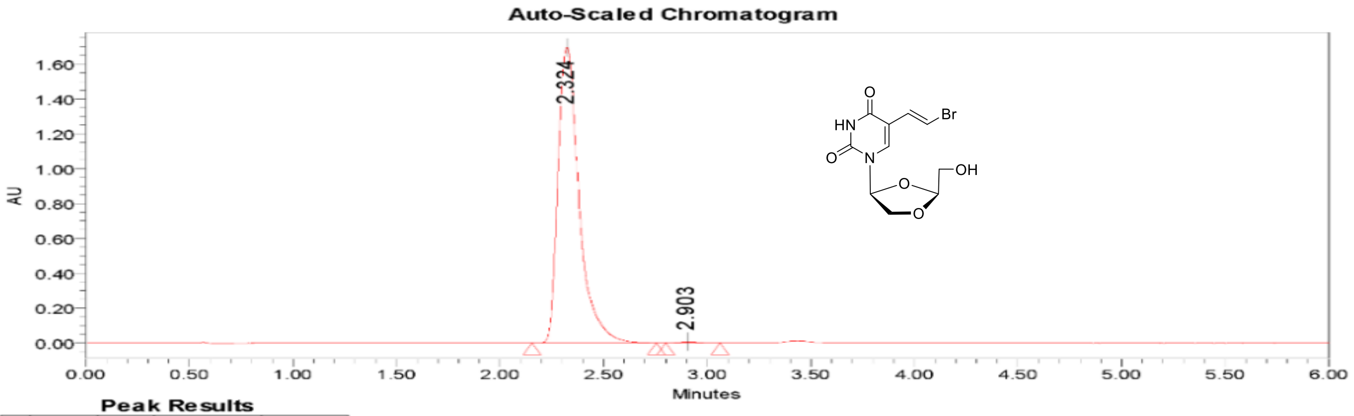

Peak Results

	Name	RT	Area	% Area
1		2.32	12025246	99.75
2		2.90	30235	0.25

Figure S34. Chiral HPLC spectrum of authentic **L-BHDU.**[1]

Chiral HPLC Method Conditions			
Column	CHIRALPAK AS-3 (4.6*150MM)3µM	Sample	YK-02-28-P-4
Co-solvent	0.5% DEA in METHANOL	Vial	1:C,5
Total Flow	3 mL/min	Injection Volume	10.00 µL
% of CO2	85	Run Time	20 minutes
% of Co-Solvent	15	Date Acquired	26-July-2023
ABPR	1500psi	Acq. Method Set:	CM2_C1_SolveB1_3g_15
Column Temperature	30 ℃		

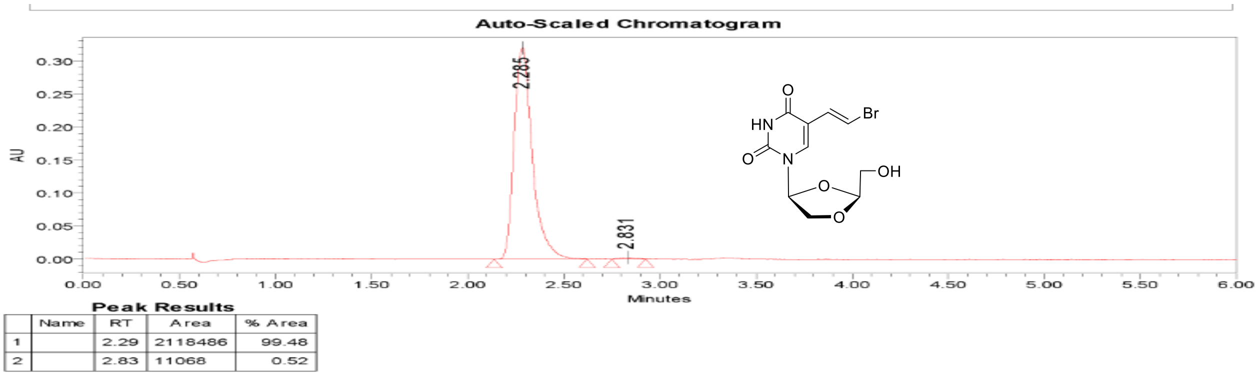

Figure S35. Chiral HPLC spectrum of L-BHDU **17** *via* reported synthetic route.

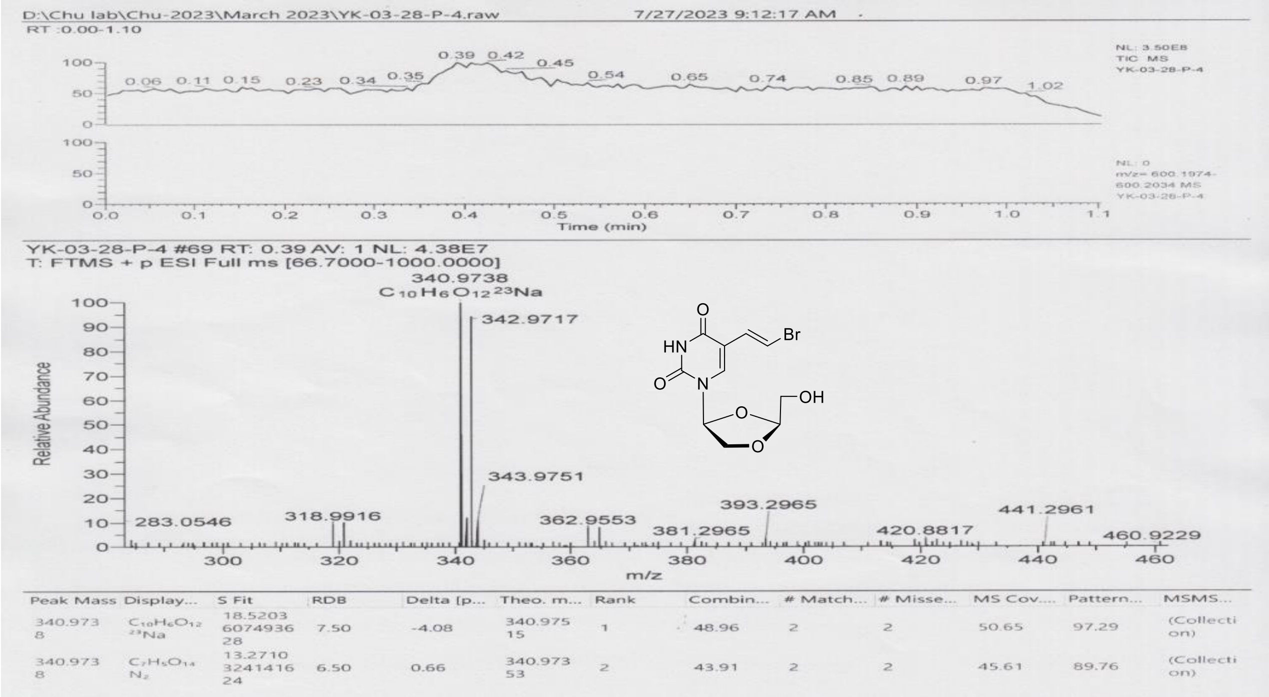

Figure S36. HRMS data of compound L-BHDU **17**

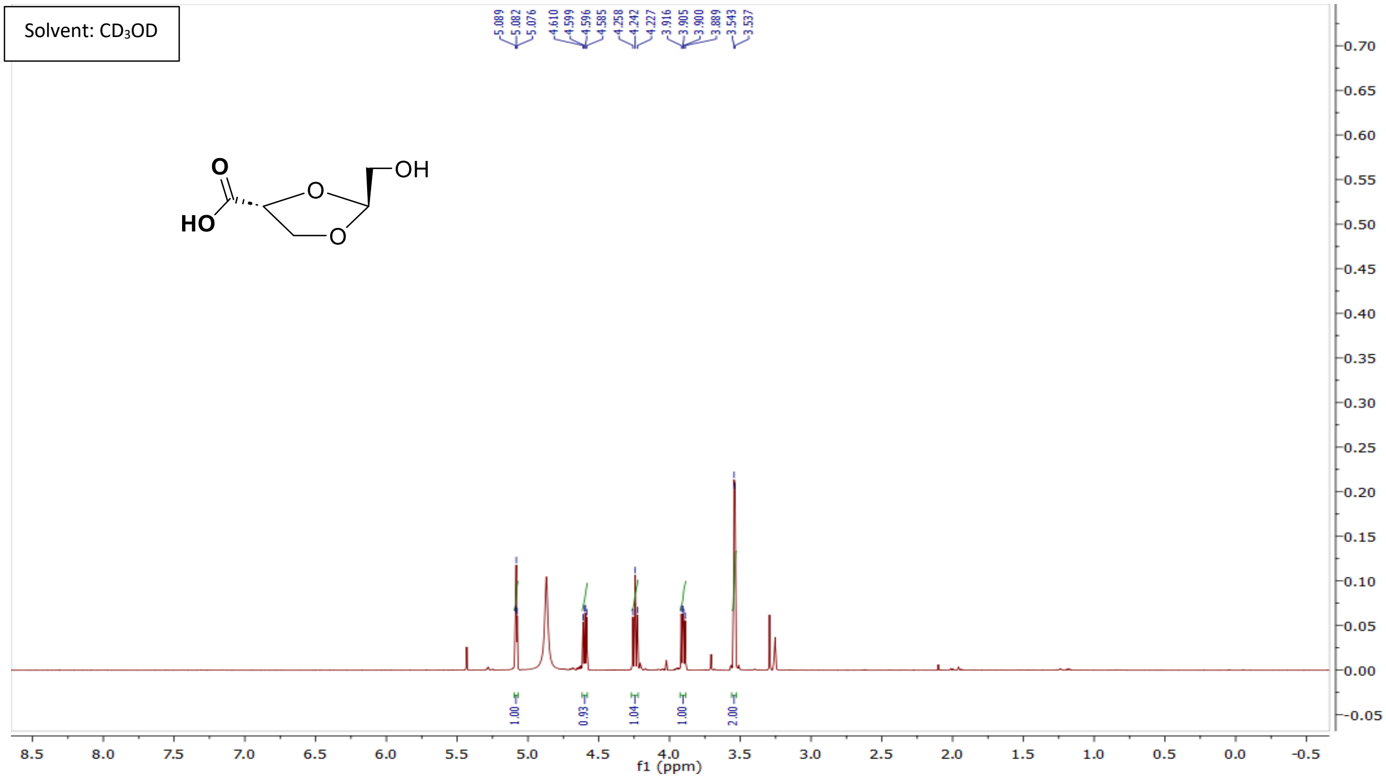

Figure S37. ^{1}H NMR (500 MHz) spectrum of compound **18**

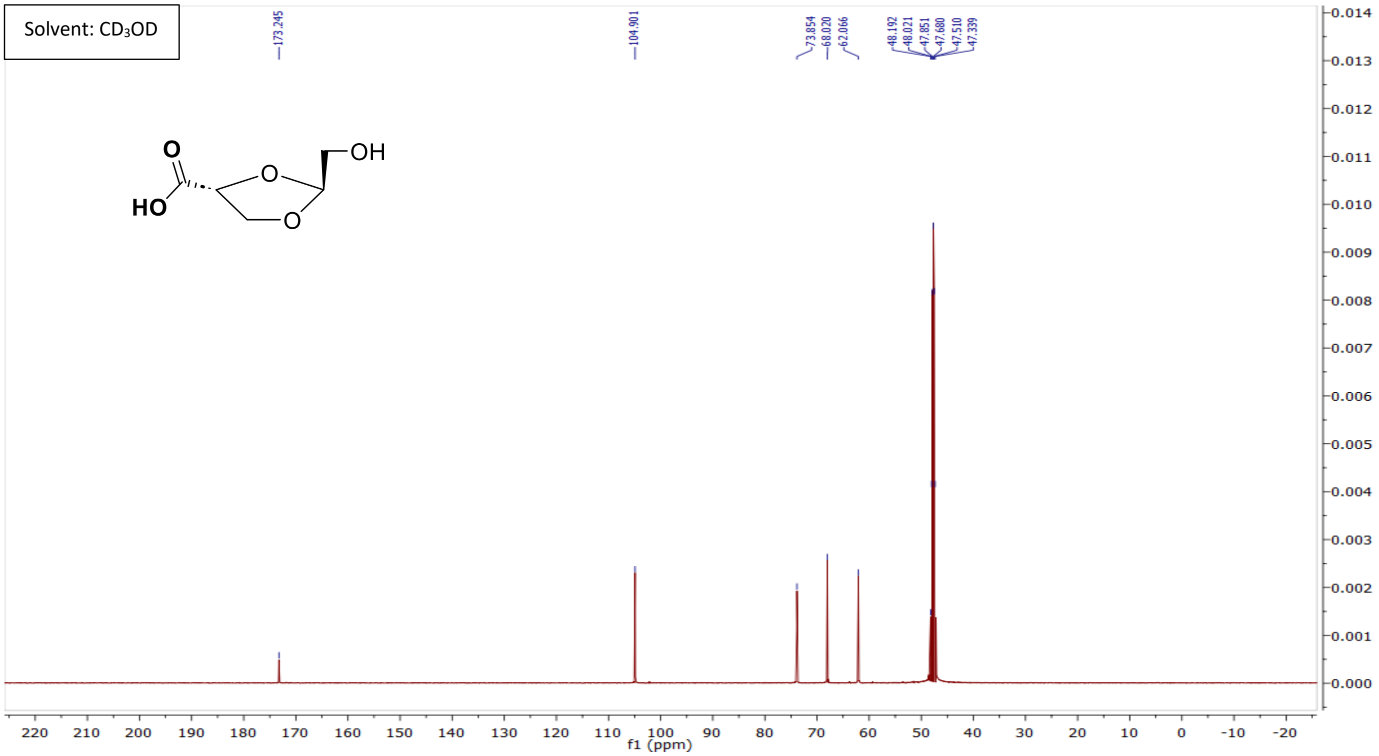

Figure S38. ^{13}C{^{1}H} (126 MHz) NMR spectrum of compound **18**

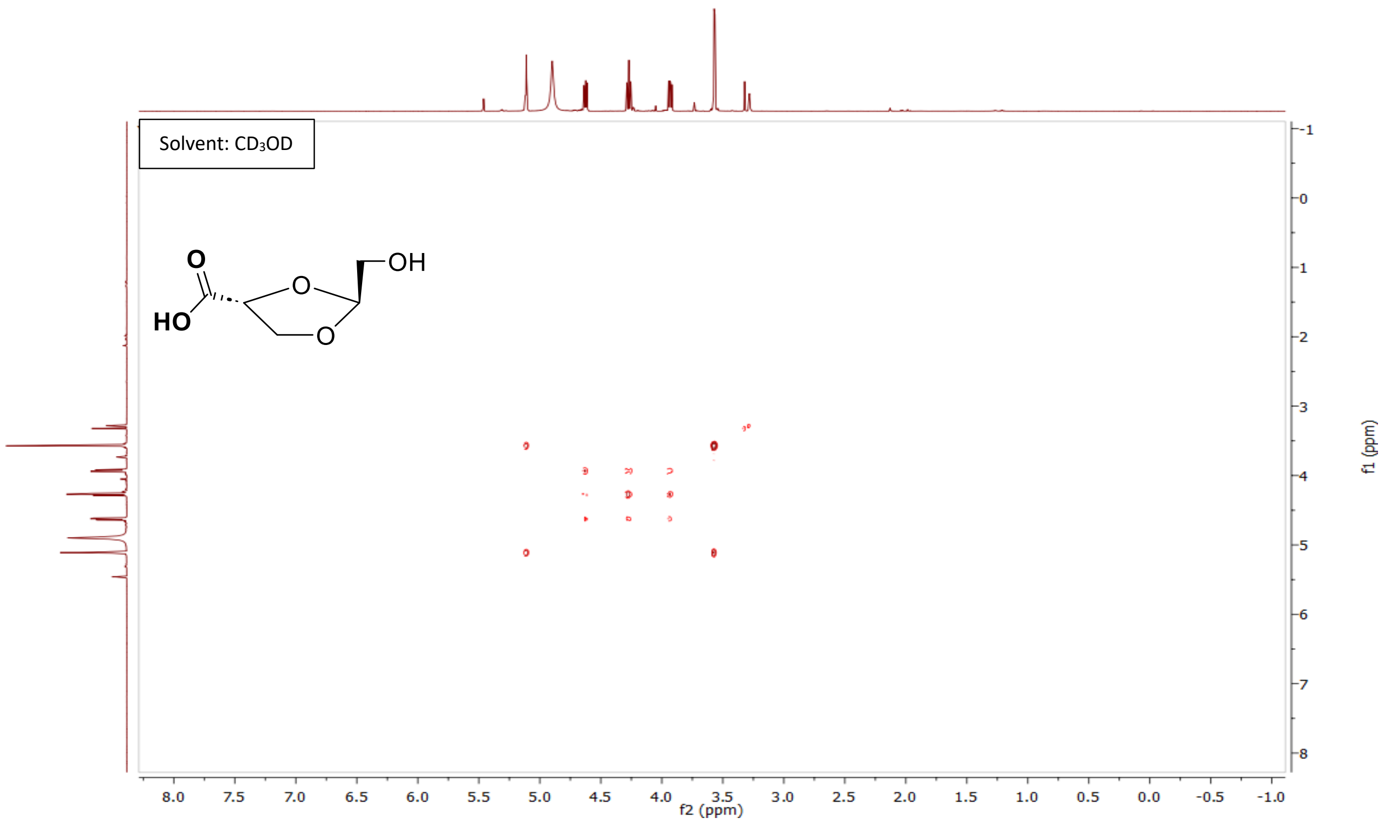

Figure S39. COSY NMR (500 MHz) spectrum of compound **18**

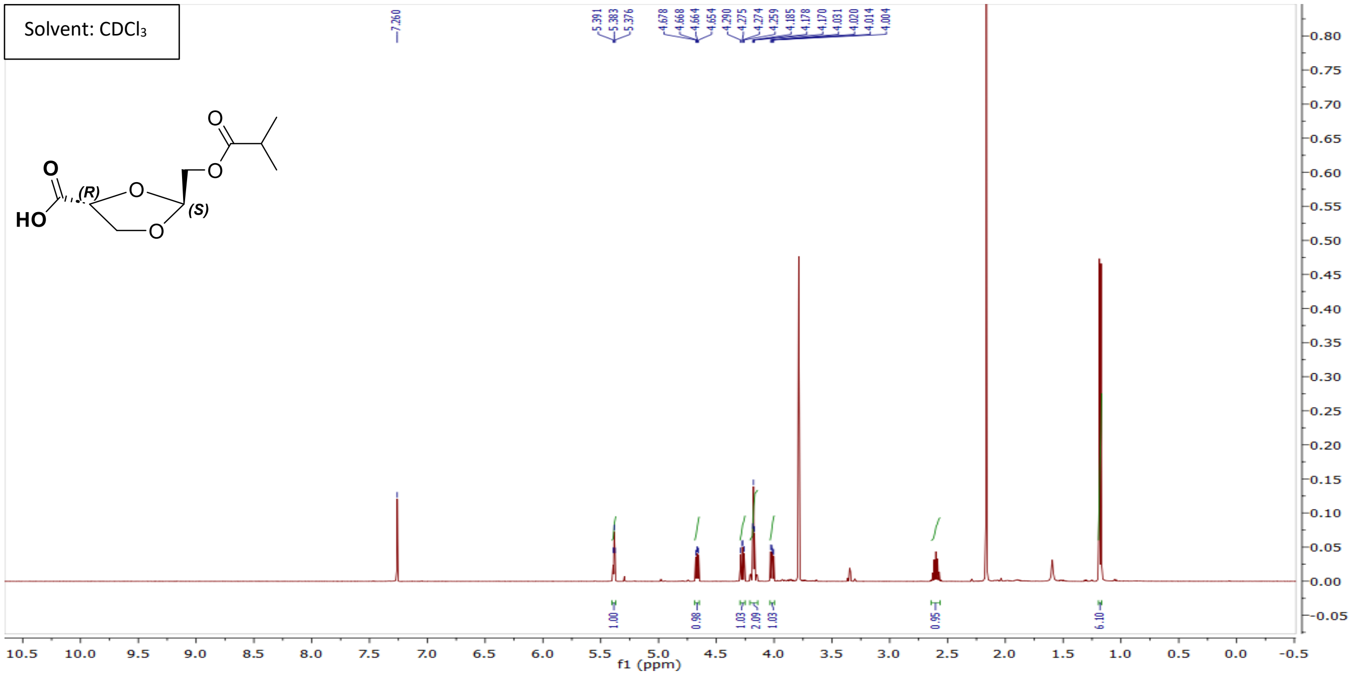

Figure S40. ^{1}H NMR (500 MHz) spectrum of compound **19**

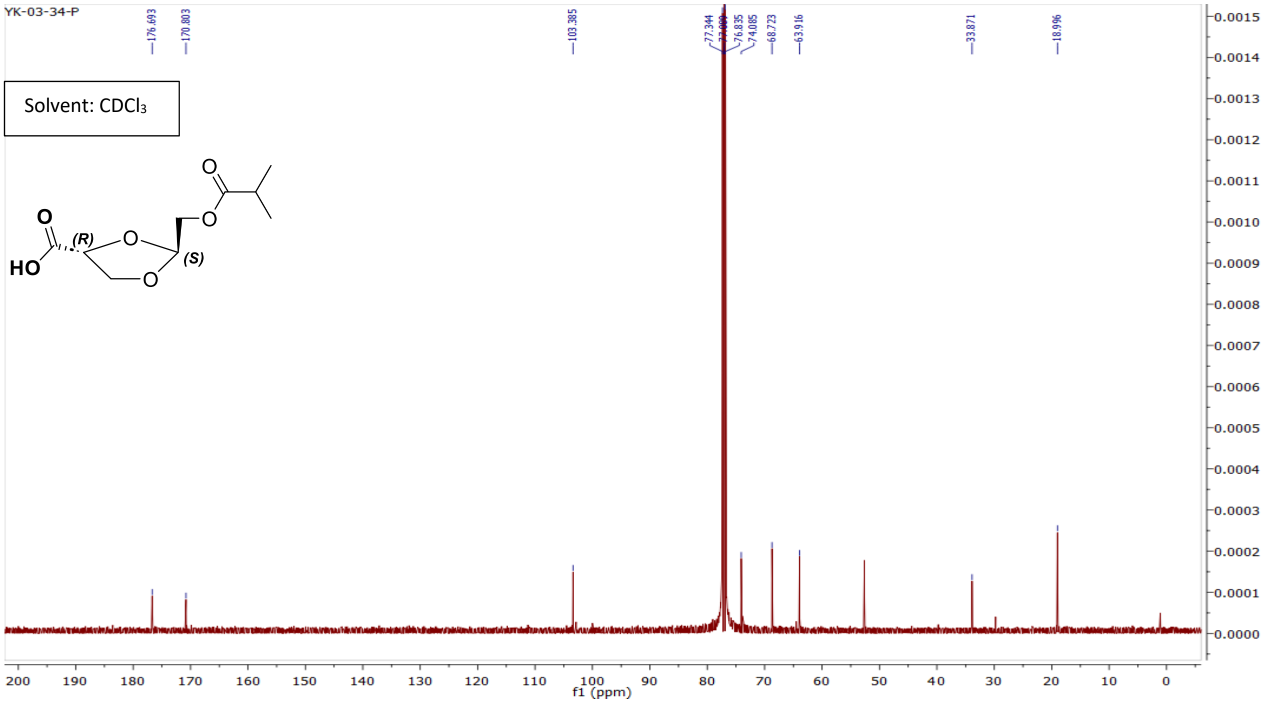

Figure S41. $^{13}C\{^{1}H\}$ (126 MHz) NMR spectrum of compound **19**

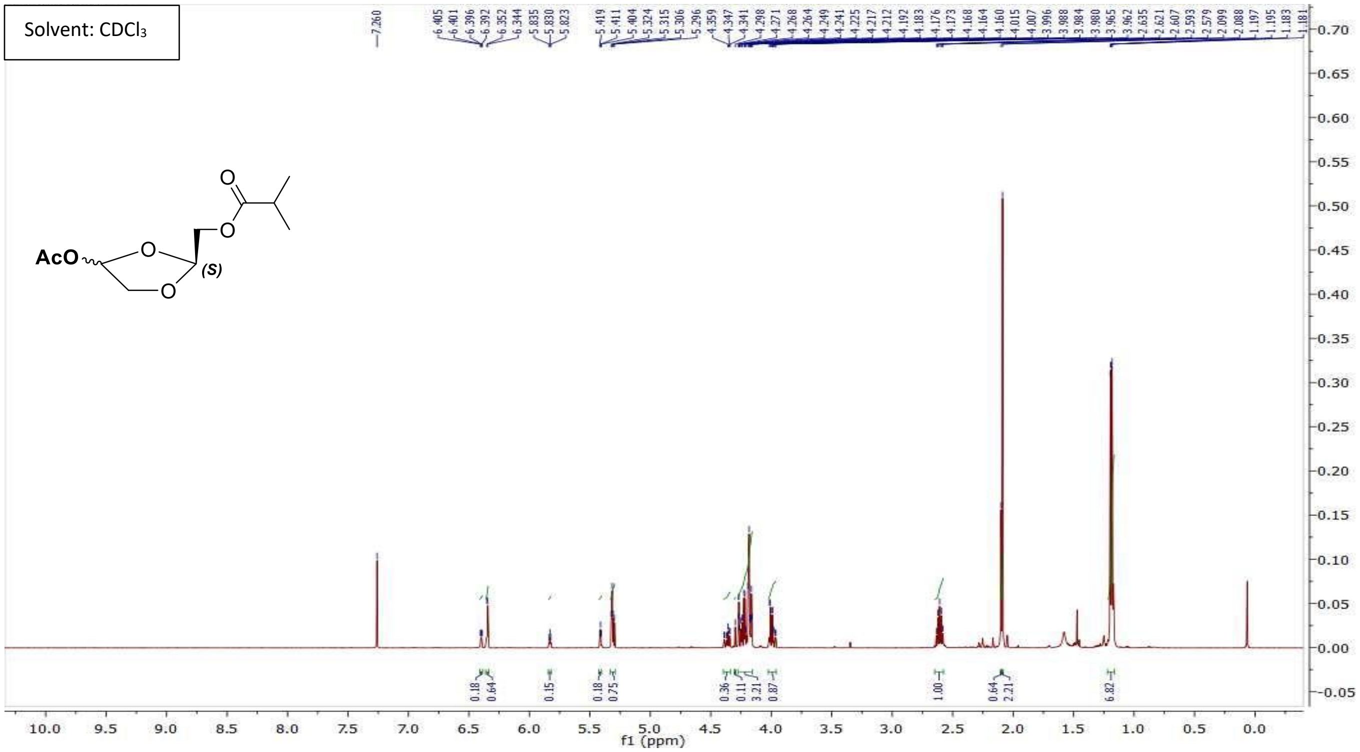

Figure S42. ^{1}H NMR (500 MHz) spectrum of compound **20**

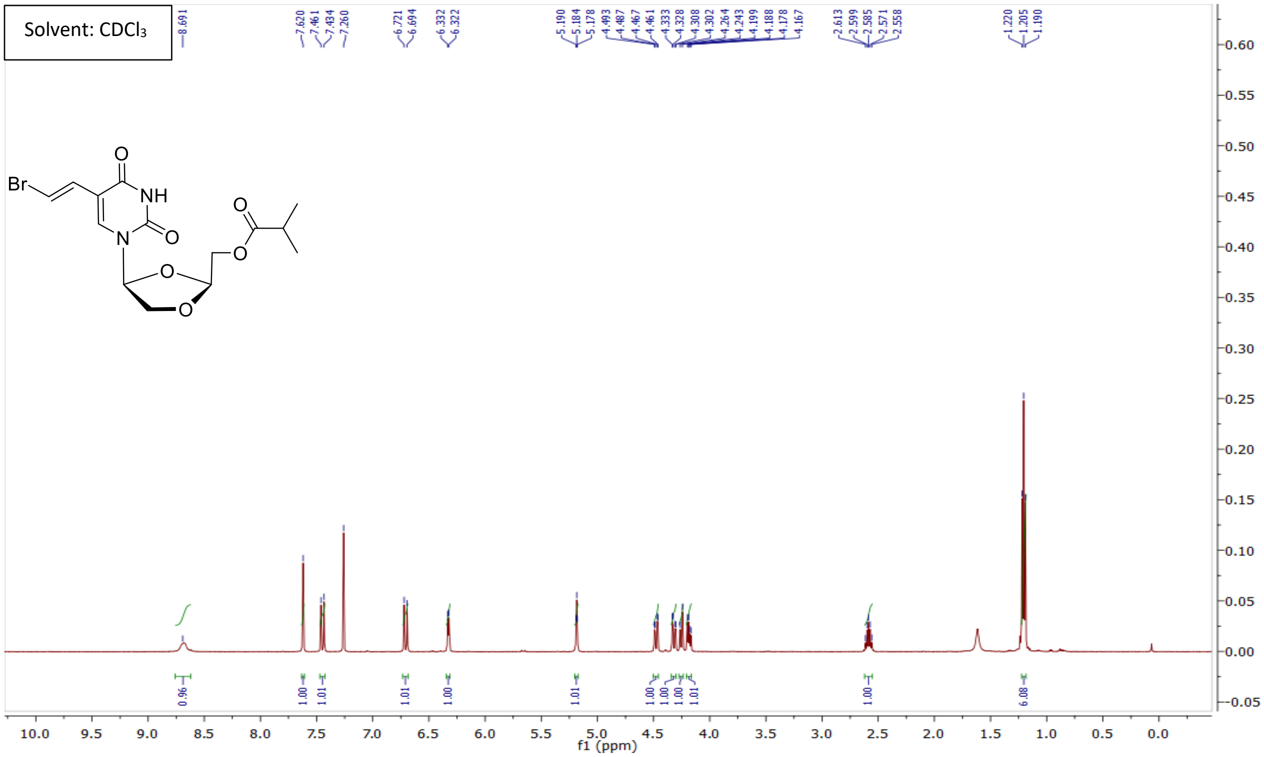

Figure S43. ^{1}H NMR (500 MHz) spectrum of compound **22**

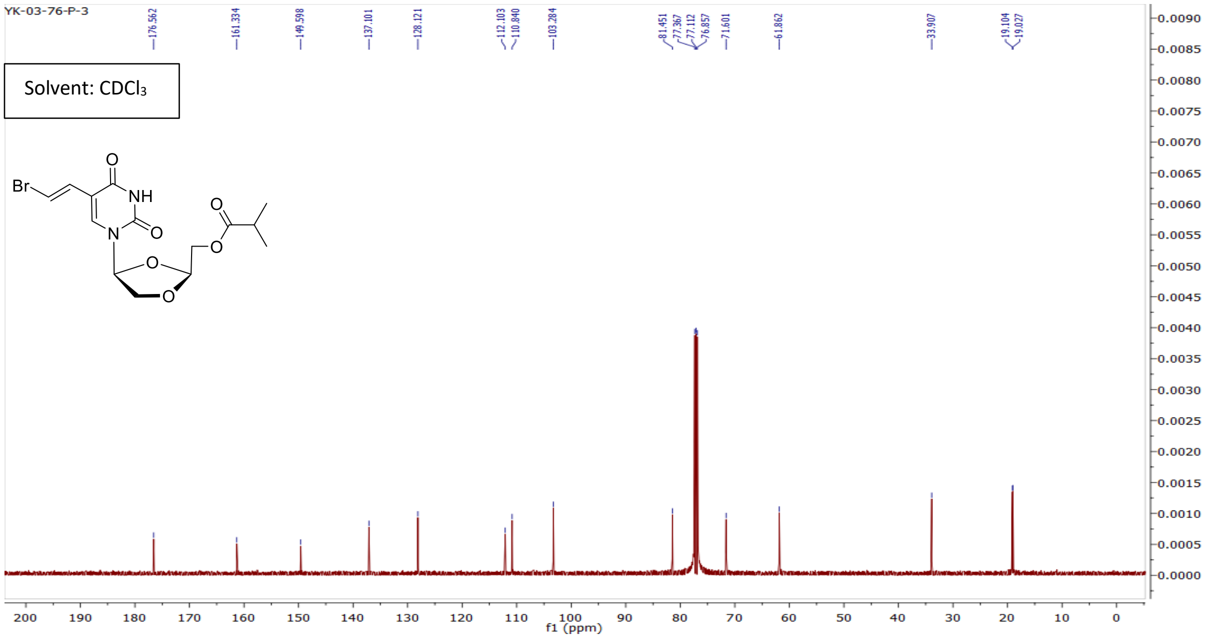

Figure S44. ^{13}C{^{1}H} (126 MHz) NMR spectrum of compound **22**

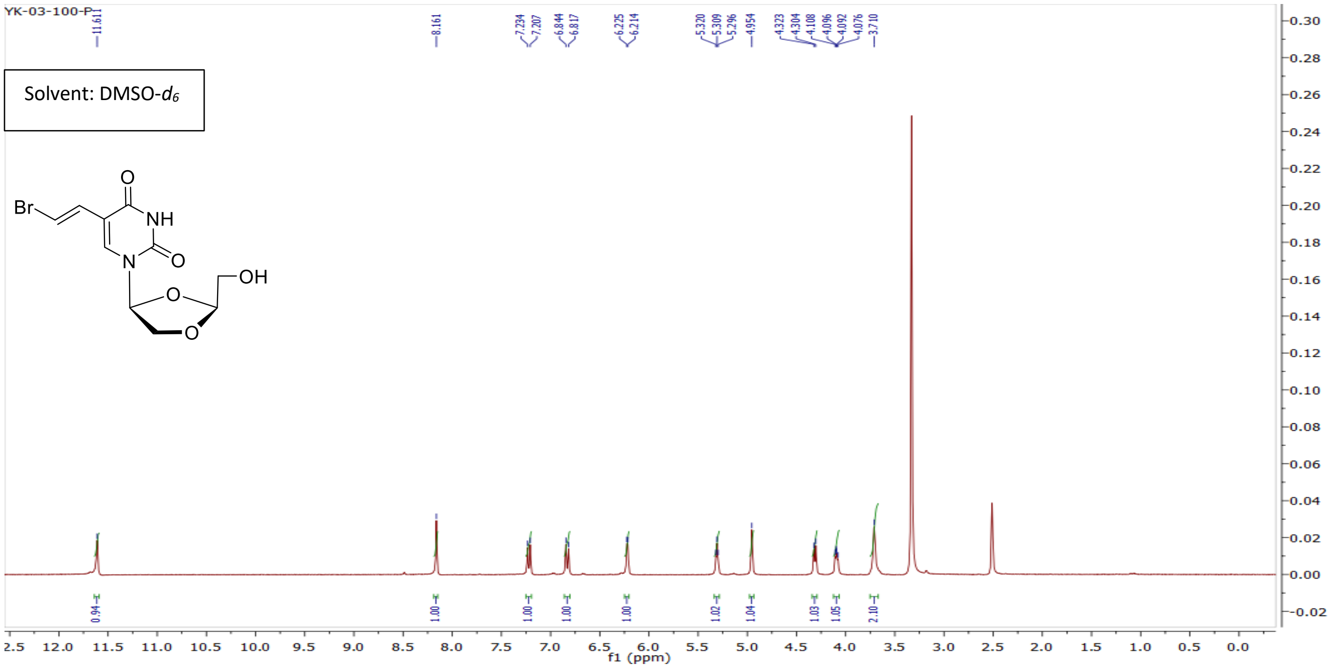

Figure S45. ^{1}H NMR (500 MHz) spectrum of compound **17 (from scheme 4)**

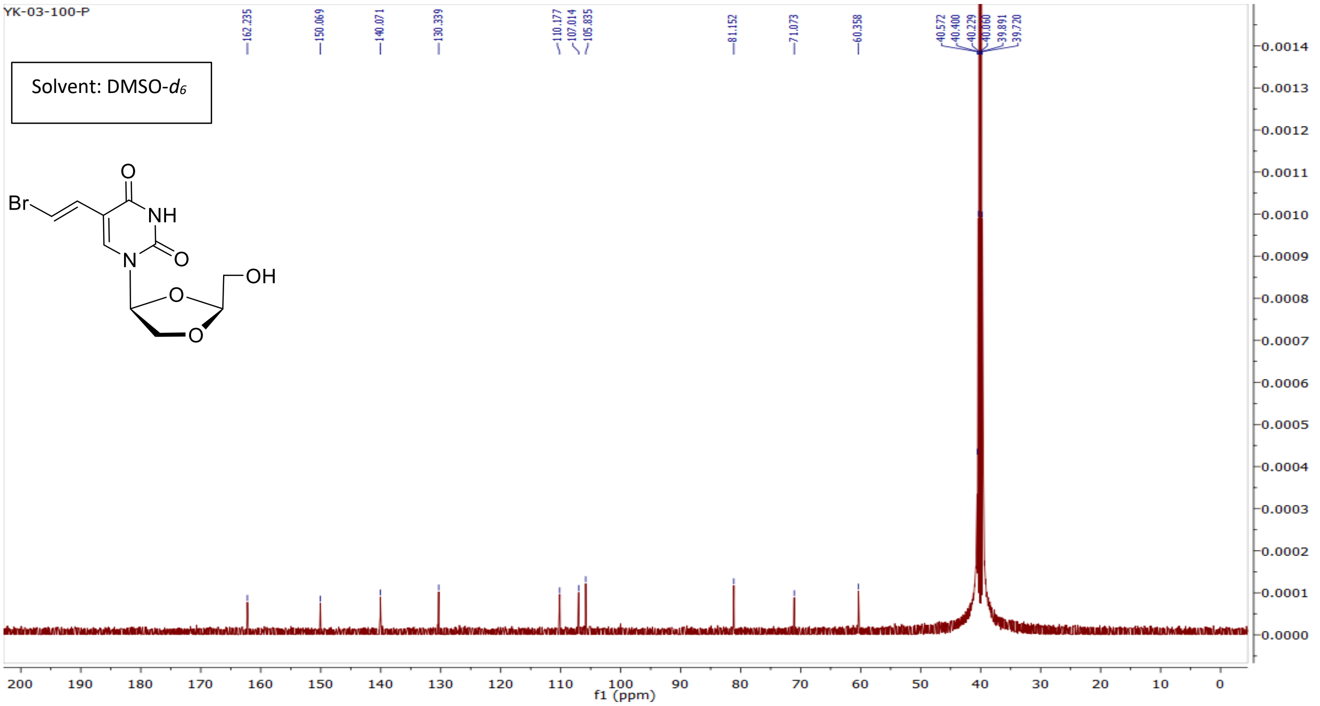

Figure S46. ^{1}H NMR (500 MHz)^{13}C{^{1}H} (126 MHz) NMR spectrum of compound **17 (from scheme 4)**

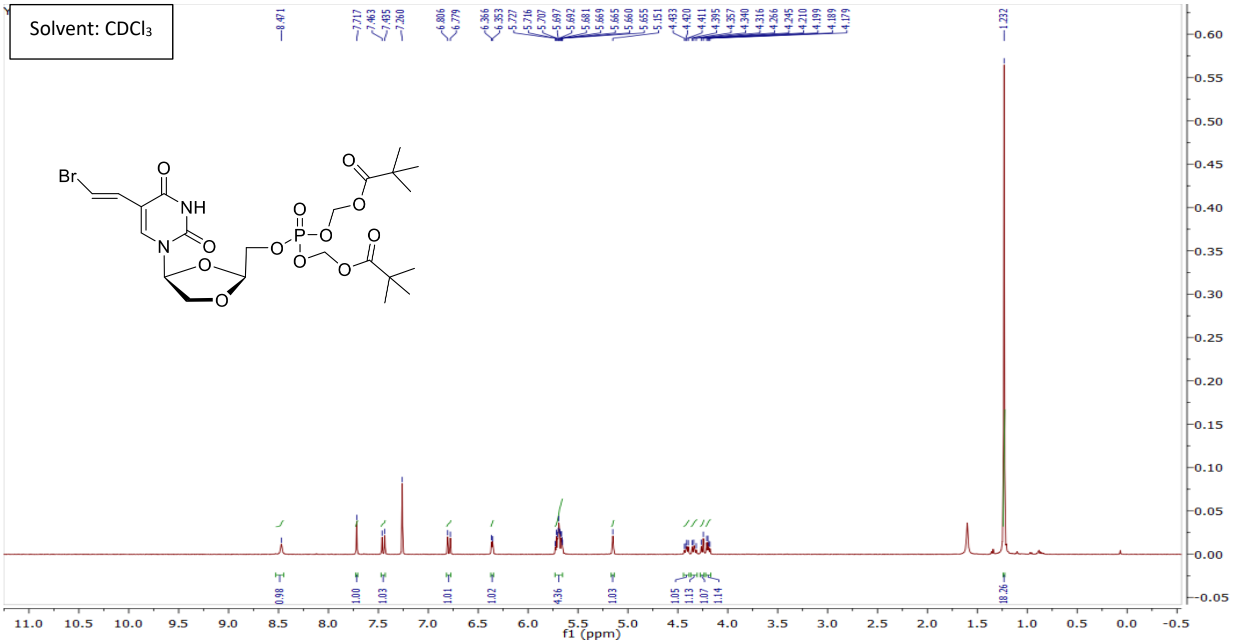

Figure S47. ^{1}H NMR (500 MHz)^{1}H NMR (500 MHz) spectrum of compound **24**

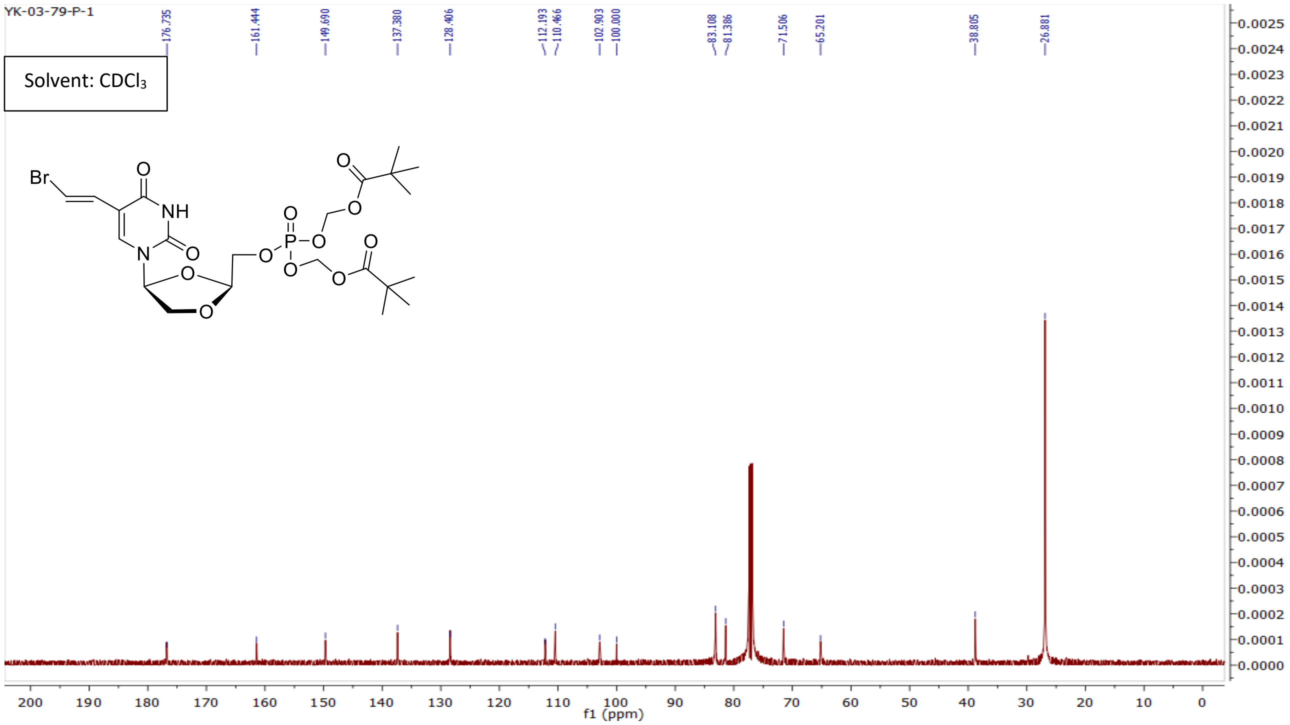

Figure S48. ^{13}C{^{1}H} (126 MHz) NMR spectrum of compound **24**

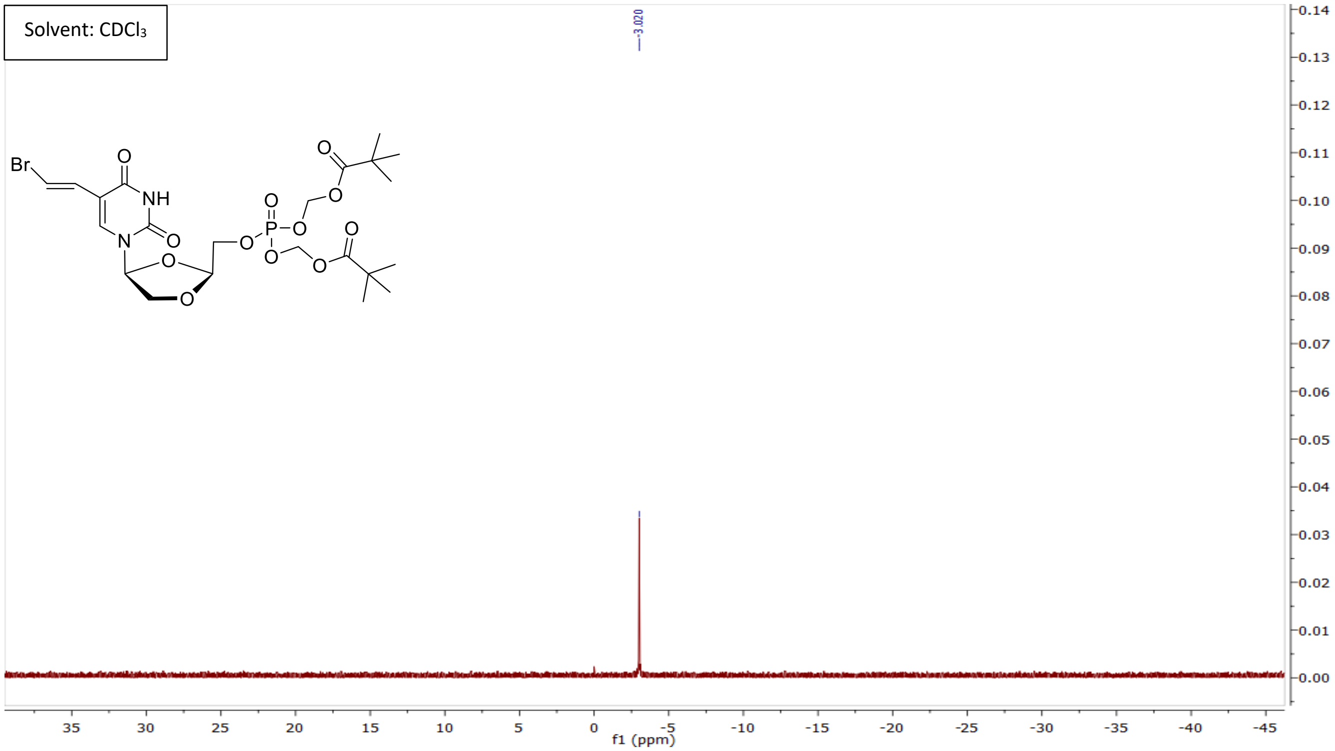

Figure S49. ^{31}P NMR (202 MHz) spectrum of compound **24**

X-Ray crystallographic information for compounds (10)

For solvent system and method for the crytal growth of compound 10, weigh 10 (15 mg) in clean 1.0 mL vial, add 0.4 mL methanol as solvent system, natural growth at room temperature.

X-Ray Crystallographic data (CCDC: 2312275)

Thermal ellipsoid probability levels = 38%

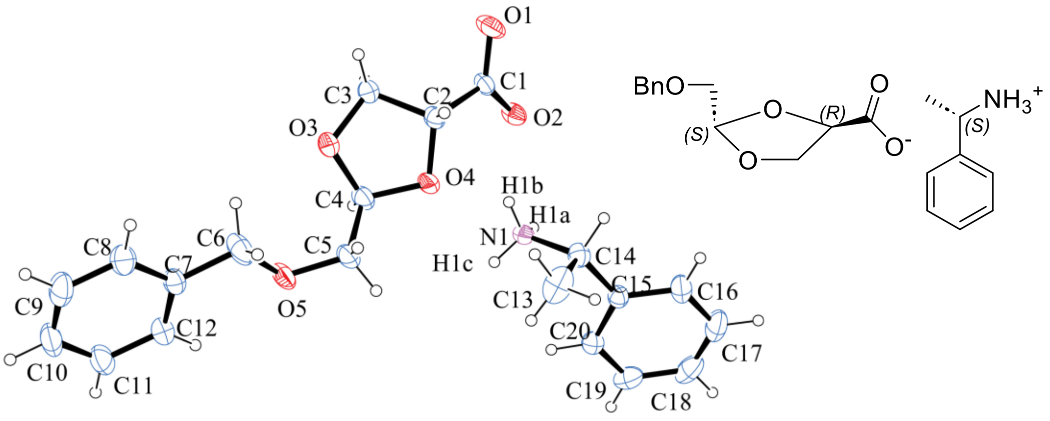

Figure S50. X-ray data of compound **10** (**CCDC: 2312275**)

Crystal Structure Report for YK-02-69-P-3

A specimen of $C_{20}H_{25}NO_5$, approximate dimensions 0.260 mm x 0.300 mm x 0.450 mm in a colorless crystal was used for the X-ray crystallographic analysis. The X-ray intensity data were measured at room temperature (297K) on a Bruker D8 Quest PHOTON 100 CMOS X-ray diffractometer system with Incoatec Microfocus Source (IμS) monochromated Mo Kα radiation (λ = 0.71073 Å, sealed tube) using phi and omega-scan technique.

The integration of the data using an orthorhombic unit cell yielded a total of 50185 reflections to a maximum θ angle of 34.34° (0.63 Å resolution), of which 8077 were independent (average redundancy 6.213, completeness = 99.8%, R_{int} = 4.42%, R_{sig} = 3.03%) and 5774 (71.49%) were greater than 2σ(F^2). The final cell constants of a = 6.6227(4) Å, b = 10.6136(7) Å, c =

27.4644(17) Å, volume = 1930.5(2) Å^3, are based upon the refinement of the XYZ-centroids of 9909 reflections above 20 σ(I). The data were integrated with the manufacturer's SAINT software and corrected for absorption effects using the Multi-Scan method (SADABS). The calculated minimum and maximum transmission coefficients (based on crystal size) are 0.6461 and 0.7469.

The structure was solved and refined using the Bruker SHELXTL Software Package,[2] using the space group $P2_12_12_1$ (No. 19), with Z = 4 for the formula unit, $C_{20}H_{25}NO_5$. Non-hydrogen atoms were located from successive difference Fourier map calculations. In the final cycles of each refinement, all the non-hydrogen atoms were refined in anisotropic displacement parameters. While three hydrogen atoms (H(1A), H(1B), H(1C)) on the atom of N(1) were located from difference Fourier map and refined with proper restraints, the rest of the hydrogen atom positions were calculated and allowed to ride on the carbon to which they are bonded assuming a C–H bond length of m Å (m = 0.930 for Ph-H groups, m = 0.980 for CH groups, m = 0.970 for CH_2 groups, m = 0.960 for CH_3 groups). Hydrogen atom temperature factors were fixed at n (n = 1.2 for Ph-H, CH, CH_2 and n = 1.5 for CH_3) times the isotropic temperature factors of the C-atoms to which they are bonded. The final anisotropic full-matrix least-squares refinement on F^2 with 247 variables converged at R1 = 5.59%, for the observed data and wR2 = 15.78% for all data. The goodness-of-fit was 1.007. The largest peak in the final difference electron density synthesis was 0.289 $e^-/Å^3$ and the largest hole was -0.179 $e^-/Å^3$ with an RMS deviation of 0.039 $e^-/Å^3$. These largest residues are of no chemical significance. On the basis of the final model, the calculated density was 1.237 g/cm^3 and F(000), 768 e^-. The asymmetric unit contains one molecule in the form of cation/anion with a formula of $C_{20}H_{25}NO_5$. There might exist some hydrogen bonding between the cation/anion as well as between the molecules in the lattice. The efforts have been made to resolve as many alerts as possible generated by CheckCIF. The current highest alerts are at level C, which might

be attributed to a little bit high thermal motions of some atoms of molecule. The absolute structure of molecule can't be determined reliably due to the weak anomalous scatterings from the molecule.

Table S1. Crystal data and structure refinement for YK-02-69-P-3 (**Compound 10**).

Identification code	YK-02-69-P-3
Empirical formula	C20 H25 N O5
Formula weight	359.41
Temperature	297(2) K
Wavelength	0.71073 A
Crystal system, space group	Orthorhombic, P2(1)2(1)2(1)
Unit cell dimensions	a = 6.6227(4) A alpha = 90 deg.<br>b = 10.6136(7) A beta = 90 deg.<br>c = 27.4644(17) A gamma = 90 deg.
Volume	1930.5(2) A^3
Z, Calculated density	4, 1.237 Mg/m^3
Absorption coefficient	0.089 mm^-1
F(000)	768
Crystal size	0.450 x 0.300 x 0.260 mm
Theta range for data collection	2.425 to 34.337 deg.
Limiting indices	-10<=h<=10, -16<=k<=16, -43<=l<=43
Reflections collected / unique	50185 / 8077 [R(int) = 0.0442]
Completeness to theta = 25.242	99.8 %
Absorption correction	Semi-empirical from equivalents
Max. and min. transmission	0.7469 and 0.6461
Refinement method	Full-matrix least-squares on F^2

Data / restraints / parameters	8077 / 3 / 247
Goodness-of-fit on F^2	1.007
Final R indices [I>2sigma(I)]	R1 = 0.0559, wR2 = 0.1425
R indices (all data)	R1 = 0.0833, wR2 = 0.1578
Absolute structure parameter	0.2(2)
Extinction coefficient	n/a
Largest diff. peak and hole	0.289 and -0.179 e.A^-3

Table S2. Atomic coordinates (x 10^4) and equivalent isotropic displacement parameters (A^2 x 10^3) for j1_a.
U(eq) is defined as one-third of the trace of the orthogonalized Uij tensor.

	x	y	z	U(eq)
O(1)	-832(2)	4665(2)	5371(1)	72(1)
O(2)	2065(2)	3898(1)	5106(1)	43(1)
O(3)	3491(3)	6602(2)	6238(1)	55(1)
O(4)	4157(2)	5865(2)	5477(1)	57(1)
O(5)	7529(2)	7675(2)	6260(1)	54(1)
N(1)	6050(2)	3661(1)	4835(1)	34(1)
C(1)	1024(3)	4702(2)	5318(1)	38(1)
C(2)	2029(3)	5844(2)	5542(1)	38(1)
C(3)	1787(3)	5875(2)	6091(1)	48(1)
C(4)	5066(3)	6214(2)	5926(1)	46(1)
C(5)	6494(3)	7283(2)	5834(1)	51(1)
C(6)	6531(4)	8627(3)	6523(1)	68(1)
C(7)	7875(3)	9150(2)	6915(1)	44(1)
C(8)	7102(5)	10105(2)	7209(1)	55(1)
C(9)	8284(6)	10635(3)	7568(1)	66(1)
C(10)	10233(6)	10244(3)	7635(1)	68(1)
C(11)	11005(5)	9305(3)	7346(1)	62(1)
C(12)	9832(4)	8755(2)	6986(1)	49(1)
C(13)	6362(7)	5555(2)	4335(1)	74(1)
C(14)	5934(3)	4150(2)	4326(1)	43(1)
C(15)	7312(3)	3425(2)	3991(1)	41(1)
C(16)	6627(4)	3054(2)	3536(1)	54(1)
C(17)	7870(6)	2391(3)	3223(1)	69(1)
C(18)	9792(6)	2089(3)	3362(1)	70(1)
C(19)	10512(4)	2459(3)	3810(1)	62(1)
C(20)	9287(3)	3138(2)	4121(1)	48(1)

Table S3. Bond lengths [A] and angles [deg] for **YK-02-69-P-3** (Compound **10**).

O(1)-C(1)	1.238(2)
O(2)-C(1)	1.241(2)
O(3)-C(4)	1.412(3)
O(3)-C(3)	1.425(3)
O(4)-C(2)	1.421(2)
O(4)-C(4)	1.421(2)
O(5)-C(6)	1.407(3)
O(5)-C(5)	1.417(3)
N(1)-C(14)	1.494(3)
N(1)-H(1A)	0.87(2)
N(1)-H(1B)	0.84(2)
N(1)-H(1C)	0.875(19)
C(1)-C(2)	1.515(2)
C(2)-C(3)	1.515(3)
C(2)-H(2A)	0.9800
C(3)-H(3A)	0.9700
C(3)-H(3B)	0.9700
C(4)-C(5)	1.499(3)
C(4)-H(4A)	0.9800
C(5)-H(5A)	0.9700
C(5)-H(5B)	0.9700
C(6)-C(7)	1.503(3)
C(6)-H(6A)	0.9700
C(6)-H(6B)	0.9700
C(7)-C(12)	1.376(3)
C(7)-C(8)	1.392(3)
C(8)-C(9)	1.379(4)
C(8)-H(8A)	0.9300
C(9)-C(10)	1.368(5)
C(9)-H(9A)	0.9300
C(10)-C(11)	1.373(4)
C(10)-H(10A)	0.9300
C(11)-C(12)	1.387(3)
C(11)-H(11A)	0.9300
C(12)-H(12A)	0.9300
C(13)-C(14)	1.518(3)
C(13)-H(13A)	0.9600
C(13)-H(13B)	0.9600
C(13)-H(13C)	0.9600
C(14)-C(15)	1.507(3)
C(14)-H(14A)	0.9800
C(15)-C(20)	1.389(3)

C(15)-C(16)	1.386(3)
C(16)-C(17)	1.383(4)
C(16)-H(16A)	0.9300
C(17)-C(18)	1.367(5)
C(17)-H(17A)	0.9300
C(18)-C(19)	1.379(4)
C(18)-H(18A)	0.9300
C(19)-C(20)	1.380(3)
C(19)-H(19A)	0.9300
C(20)-H(20A)	0.9300
C(4)-O(3)-C(3)	104.80(15)
C(2)-O(4)-C(4)	108.36(15)
C(6)-O(5)-C(5)	114.06(18)
C(14)-N(1)-H(1A)	110.0(19)
C(14)-N(1)-H(1B)	110.4(18)
H(1A)-N(1)-H(1B)	108(3)
C(14)-N(1)-H(1C)	110.6(15)
H(1A)-N(1)-H(1C)	111(2)
H(1B)-N(1)-H(1C)	106(2)
O(2)-C(1)-O(1)	125.76(18)
O(2)-C(1)-C(2)	119.79(17)
O(1)-C(1)-C(2)	114.46(18)
O(4)-C(2)-C(3)	103.30(16)
O(4)-C(2)-C(1)	113.38(15)
C(3)-C(2)-C(1)	112.03(17)
O(4)-C(2)-H(2A)	109.3
C(3)-C(2)-H(2A)	109.3
C(1)-C(2)-H(2A)	109.3
O(3)-C(3)-C(2)	102.08(17)
O(3)-C(3)-H(3A)	111.4
C(2)-C(3)-H(3A)	111.4
O(3)-C(3)-H(3B)	111.4
C(2)-C(3)-H(3B)	111.4
H(3A)-C(3)-H(3B)	109.2
O(3)-C(4)-O(4)	106.80(16)
O(3)-C(4)-C(5)	110.34(18)
O(4)-C(4)-C(5)	108.60(19)
O(3)-C(4)-H(4A)	110.3
O(4)-C(4)-H(4A)	110.3
C(5)-C(4)-H(4A)	110.3
O(5)-C(5)-C(4)	112.9(2)
O(5)-C(5)-H(5A)	109.0
C(4)-C(5)-H(5A)	109.0
O(5)-C(5)-H(5B)	109.0
C(4)-C(5)-H(5B)	109.0

H(5A)-C(5)-H(5B)	107.8
O(5)-C(6)-C(7)	110.82(19)
O(5)-C(6)-H(6A)	109.5
C(7)-C(6)-H(6A)	109.5
O(5)-C(6)-H(6B)	109.5
C(7)-C(6)-H(6B)	109.5
H(6A)-C(6)-H(6B)	108.1
C(12)-C(7)-C(8)	119.1(2)
C(12)-C(7)-C(6)	123.1(2)
C(8)-C(7)-C(6)	117.8(2)
C(9)-C(8)-C(7)	120.2(3)
C(9)-C(8)-H(8A)	119.9
C(7)-C(8)-H(8A)	119.9
C(10)-C(9)-C(8)	120.6(3)
C(10)-C(9)-H(9A)	119.7
C(8)-C(9)-H(9A)	119.7
C(9)-C(10)-C(11)	119.6(2)
C(9)-C(10)-H(10A)	120.2
C(11)-C(10)-H(10A)	120.2
C(10)-C(11)-C(12)	120.6(3)
C(10)-C(11)-H(11A)	119.7
C(12)-C(11)-H(11A)	119.7
C(7)-C(12)-C(11)	120.0(2)
C(7)-C(12)-H(12A)	120.0
C(11)-C(12)-H(12A)	120.0
C(14)-C(13)-H(13A)	109.5
C(14)-C(13)-H(13B)	109.5
H(13A)-C(13)-H(13B)	109.5
C(14)-C(13)-H(13C)	109.5
H(13A)-C(13)-H(13C)	109.5
H(13B)-C(13)-H(13C)	109.5
N(1)-C(14)-C(15)	111.28(16)
N(1)-C(14)-C(13)	108.43(18)
C(15)-C(14)-C(13)	113.5(2)
N(1)-C(14)-H(14A)	107.8
C(15)-C(14)-H(14A)	107.8
C(13)-C(14)-H(14A)	107.8
C(20)-C(15)-C(16)	118.5(2)
C(20)-C(15)-C(14)	121.71(18)
C(16)-C(15)-C(14)	119.8(2)
C(17)-C(16)-C(15)	120.6(3)
C(17)-C(16)-H(16A)	119.7
C(15)-C(16)-H(16A)	119.7
C(16)-C(17)-C(18)	120.1(2)
C(16)-C(17)-H(17A)	120.0
C(18)-C(17)-H(17A)	120.0

C(17)-C(18)-C(19)	120.3(3)
C(17)-C(18)-H(18A)	119.9
C(19)-C(18)-H(18A)	119.9
C(20)-C(19)-C(18)	119.8(3)
C(20)-C(19)-H(19A)	120.1
C(18)-C(19)-H(19A)	120.1
C(19)-C(20)-C(15)	120.7(2)
C(19)-C(20)-H(20A)	119.7
C(15)-C(20)-H(20A)	119.7

Symmetry transformations used to generate equivalent atoms:

Table S4. Anisotropic displacement parameters (A^2 x 10^3) for YK-02-69-P-3 (Compound 10). The anisotropic displacement factor exponent takes the form: -2 pi^2 [h^2 a*^2 U11 + ... + 2 h k a* b* U12]

	U11	U22	U33	U23	U13	U12
O(1)	30(1)	89(1)	96(1)	-49(1)	-3(1)	-5(1)
O(2)	36(1)	33(1)	61(1)	-15(1)	-2(1)	0(1)
O(3)	50(1)	66(1)	48(1)	-24(1)	0(1)	-17(1)
O(4)	38(1)	80(1)	55(1)	-35(1)	6(1)	-20(1)
O(5)	38(1)	55(1)	69(1)	-30(1)	-12(1)	0(1)
N(1)	31(1)	30(1)	42(1)	-6(1)	1(1)	0(1)
C(1)	31(1)	38(1)	43(1)	-12(1)	-8(1)	-1(1)
C(2)	36(1)	35(1)	43(1)	-11(1)	-3(1)	-1(1)
C(3)	43(1)	56(1)	45(1)	-17(1)	2(1)	-11(1)
C(4)	40(1)	46(1)	54(1)	-16(1)	-6(1)	-5(1)
C(5)	41(1)	55(1)	56(1)	-20(1)	-4(1)	-10(1)
C(6)	49(1)	66(2)	90(2)	-43(2)	-19(1)	11(1)
C(7)	50(1)	36(1)	46(1)	-7(1)	-4(1)	-5(1)
C(8)	66(2)	41(1)	59(1)	-10(1)	-1(1)	1(1)
C(9)	96(2)	50(1)	53(1)	-16(1)	3(1)	-8(1)
C(10)	88(2)	72(2)	45(1)	-11(1)	-16(1)	-21(2)
C(11)	64(2)	72(2)	50(1)	0(1)	-18(1)	- 10(1)
C(12)	54(1)	46(1)	47(1)	-5(1)	-8(1)	-2(1)
C(13)	120(3)	39(1)	62(2)	7(1)	12(2)	13(1)
C(14)	42(1)	44(1)	43(1)	-1(1)	-5(1)	6(1)
C(15)	46(1)	38(1)	38(1)	-2(1)	-1(1)	-4(1)
C(16)	67(1)	55(1)	41(1)	-3(1)	-10(1)	-5(1)
C(17)	103(2)	63(2)	40(1)	-11(1)	5(1)	-12(2)
C(18)	92(2)	60(1)	58(1)	-9(1)	32(2)	-1(2)
C(19)	53(1)	62(1)	71(2)	-1(1)	19(1)	1(1)
C(20)	44(1)	54(1)	46(1)	-8(1)	1(1)	-2(1)

Table S5. Hydrogen coordinates (x 10^4) and isotropic displacement parameters (A^2 x 10^3) for YK-02-69-P-3 (Compound 10).

	x	y	z	U(eq)
H(1A)	6140(40)	2850(20)	4833(10)	57(8)
H(1B)	5010(30)	3870(30)	4993(9)	49(7)
H(1C)	7080(30)	3990(20)	4989(8)	35(5)
H(2A)	1443	6611	5402	46
H(3A)	531	6278	6185	58
H(3B)	1831	5033	6228	58
H(4A)	5789	5496	6067	56
H(5A)	7475	7027	5591	61
H(5B)	5743	7991	5703	61
H(6A)	5315	8286	6669	82
H(6B)	6140	9299	6303	82
H(8A)	5786	10386	7163	66
H(9A)	7753	11263	7766	80
H(10A)	11029	10612	7875	82
H(11A)	12328	9036	7392	74
H(12A)	10368	8118	6793	59
H(13A)	6294	5884	4010	111
H(13B)	5377	5970	4535	111
H(13C)	7686	5699	4466	111
H(14A)	4546	4032	4211	52
H(16A)	5318	3254	3440	65
H(17A)	7398	2151	2918	83
H(18A)	10618	1632	3152	84
H(19A)	11820	2251	3904	74
H(20A)	9787	3405	4420	58

Table S6. Torsion angles [deg] for YK-02-69-P-3 (Compound 10).

C(4)-O(4)-C(2)-C(3)	-14.2(2)
C(4)-O(4)-C(2)-C(1)	-135.66(19)
O(2)-C(1)-C(2)-O(4)	0.4(3)
O(1)-C(1)-C(2)-O(4)	180.0(2)
O(2)-C(1)-C(2)-C(3)	-116.0(2)
O(1)-C(1)-C(2)-C(3)	63.5(3)
C(4)-O(3)-C(3)-C(2)	-38.4(2)
O(4)-C(2)-C(3)-O(3)	32.1(2)
C(1)-C(2)-C(3)-O(3)	154.51(17)
C(3)-O(3)-C(4)-O(4)	30.6(2)
C(3)-O(3)-C(4)-C(5)	148.5(2)
C(2)-O(4)-C(4)-O(3)	-9.3(3)
C(2)-O(4)-C(4)-C(5)	-128.3(2)
C(6)-O(5)-C(5)-C(4)	-90.2(3)
O(3)-C(4)-C(5)-O(5)	65.1(2)
O(4)-C(4)-C(5)-O(5)	-178.15(18)
C(5)-O(5)-C(6)-C(7)	-170.0(2)
O(5)-C(6)-C(7)-C(12)	1.9(4)
O(5)-C(6)-C(7)-C(8)	180.0(2)
C(12)-C(7)-C(8)-C(9)	-0.6(4)
C(6)-C(7)-C(8)-C(9)	-178.7(3)
C(7)-C(8)-C(9)-C(10)	1.0(4)
C(8)-C(9)-C(10)-C(11)	-0.8(4)
C(9)-C(10)-C(11)-C(12)	0.3(4)
C(8)-C(7)-C(12)-C(11)	0.0(4)
C(6)-C(7)-C(12)-C(11)	178.1(3)
C(10)-C(11)-C(12)-C(7)	0.1(4)
N(1)-C(14)-C(15)-C(20)	-45.5(3)
C(13)-C(14)-C(15)-C(20)	77.1(3)
N(1)-C(14)-C(15)-C(16)	136.0(2)
C(13)-C(14)-C(15)-C(16)	-101.4(3)
C(20)-C(15)-C(16)-C(17)	1.3(4)
C(14)-C(15)-C(16)-C(17)	179.8(2)
C(15)-C(16)-C(17)-C(18)	0.3(4)
C(16)-C(17)-C(18)-C(19)	-1.0(4)
C(17)-C(18)-C(19)-C(20)	0.0(4)
C(18)-C(19)-C(20)-C(15)	1.7(4)
C(16)-C(15)-C(20)-C(19)	-2.3(3)
C(14)-C(15)-C(20)-C(19)	179.2(2)

Symmetry transformations used to generate equivalent atoms:

Table S7. Hydrogen bonds for YK-02-69-P-3 (Compound 10). [A and deg.].

D-H...A	d(D-H)	d(H...A)	d(D...A)	<(DHA)
N(1)-H(1A)...O(2)#1	0.87(2)	1.96(2)	2.802(2)	164(3)
N(1)-H(1B)...O(2)	0.84(2)	1.97(2)	2.753(2)	153(2)
N(1)-H(1B)...O(4)	0.84(2)	2.57(3)	3.186(2)	131(2)
N(1)-H(1C)...O(1)#2	0.875(19)	1.877(19)	2.750(2)	175(2)

Symmetry transformations used to generate equivalent atoms:
#1 x+1/2,-y+1/2,-z+1   #2 x+1,y,z

Accession Codes

CCDC 2312275 contains the supplementary crystallographic data for this paper. These data can be obtained free of charge via www.ccdc.cam.ac.uk/data_request/cif, or by emailing data_request@ccdc.cam.ac.uk, or by contacting The Cambridge Crystallographic Data Centre, 12 Union Road, Cambridge CB2 1EZ, UK; fax: +44 1223 336033.

References:

1. Choi, Y.; Li, L.; Grill, S.; Gullen, E.; Lee, C. S.; Gumina, G.; Tsujii, E.; Cheng, Y. C.; Chu, C. K. Structure-activity relationships of (E)-5-(2-bromovinyl) uracil and related pyrimidine nucleosides as antiviral agents for herpes viruses. *J. Med. Chem.* **2000**, *43* (13), 2538-2546.

2. (a) Bruker AXS Inc. APEX3 Crystallography Software Suite, **2016**, 5465 East Cheryl Parkway, Madison, WI 53711, USA; (b) Sheldrick, G.M. A Short History of SHELX, Acta Cryst. **2008**, *A64*, 112-122; (c) Sheldrick, G.M. Crystal structure refinement with SHELXL, Acta Cryst. **2015**, *C71*, 3-8
